# Genome-Resolved Functional Profiling of Osteoporosis-Associated Gut Bacteria Highlights Putative Metabolic and Immunogenic Signatures of the Gut–Bone Axis

**DOI:** 10.3390/cells15171573

**Published:** 2026-08-29

**Authors:** Rogelio F. Jiménez-Ortega, Alejandra I. Ortega-Meléndez, Juan F. Montes-García, Erasmo Negrete-Abascal, Nelly Patiño, Eric G. Ramírez-Salazar, Rosalba Sevilla-Montoya, Rafael Velázquez-Cruz, Alberto Hidalgo-Bravo

**Affiliations:** 1 Servicio de Medicina Genómica, Instituto Nacional de Rehabilitación Luis Guillermo Ibarra Ibarra (INRLGII), Mexico City 14389, Mexico; rfjimenez@inr.gob.mx; 2 Unidad Académica de Ciencias de la Salud, Universidad ETAC Campus Coacalco, Coacalco de Berriozábal México 55700, Mexico; alejandra.ortega@universidadetac.edu.mx; 3 Laboratorio de Genética, Unidad de Morfofisiología y Función, Facultad de Estudios Superiores Iztacala UNAM, Tlalnepantla de Baz México 54090, Mexico; mgjf304031894@hotmail.com (J.F.M.-G.); negretee@yahoo.com (E.N.-A.); 4 Unidad de Citometría de Flujo (UCiF), Instituto Nacional de Medicina Genómica (INMEGEN), Mexico City 14610, Mexico; lnpatino@inmegen.gob.mx; 5 The Wistar Institute, Philadelphia, PA 19104, USA; eramirezsalazar@wistar.org; 6 Departmento de Genetica y Genómica Humana, Instituto Nacional de Perinatología, Mexico City 11000, Mexico; dra.rosalbasevilla@outlook.com; 7 Laboratorio de Genómica del Metabolismo Óseo, Instituto Nacional de Medicina Genómica (INMEGEN), Mexico City 14610, Mexico; rvelazquez@inmegen.gob.mx

**Keywords:** osteoporosis, gut microbiota, gut–bone axis, functional genomics, metabolic gene clusters, carbohydrate-active enzymes

## Abstract

**Highlights:**

- In silico profiling identified function-specific gut–bone axis signatures across 26 bacterial genomes.
- Metabolic and functional profiles showed marked taxon-specific variation across bacterial genomes.

**What are the main findings?**

- These findings prioritize bacterial taxa and functions for experimental validation.
- This framework may guide future mechanistic studies of the gut–bone axis.

**What are the implications of the main findings?**

- Functional profiling may help prioritize bone-relevant microbial mechanisms beyond taxonomic classification.
- The identified in silico signatures offer targets for metagenomic, metabolomic, and experimental validation.

**Abstract:**

The gut microbiota has emerged as a potential regulator of bone metabolism, but the genome-encoded functional repertoire of osteoporosis-associated gut bacteria remains insufficiently characterized. This study performed in silico functional profiling of gut bacterial taxa associated with osteoporosis, low bone mineral density, or comparator bone-related phenotypes. Twenty candidate taxa were selected from evidence in the human microbiome and represented by 26 curated bacterial reference genomes. Genome-wide annotations were used to map predicted gut–bone axis signatures, carbohydrate-active enzyme (CAZyme) repertoires, selected Kyoto Encyclopedia of Genes and Genomes pathways, and gutSMASH-predicted metabolic gene clusters. Functional burdens were normalized as hits per 1000 annotated proteins and integrated into metabolic, immunogenic, CAZyme, KEGG, and metabolic gene cluster profiles. Twelve predicted gut–bone axis signatures were identified, comprising 3337 primary candidate protein hits and a strict high-confidence subset of 2497 hits. Dominant signatures included vitamin B12/cobalamin metabolism, folate/one-carbon metabolism, peptidoglycan/cell-wall biosynthesis, and short-chain fatty acid-related functions. *Dialister invisus*, *Dialister succinatiphilus*, *Megamonas funiformis*, and *Megamonas hypermegale* showed the strongest normalized predicted gut–bone axis signal. These hypothesis-generating findings prioritize microbial metabolic and immunogenic features for future metagenomic, metabolomic, and experimental validation studies.

## 1. Introduction

Osteoporosis is a systemic skeletal disease characterized by low bone mineral density, deterioration of bone microarchitecture, and increased risk to fragility fractures. It represents one of the most important musculoskeletal conditions associated with aging, particularly affecting postmenopausal women and older adults, due to its significant impact on disability, morbidity, mortality, and health-care burden [1]. Although the pathophysiology of osteoporosis has classically been linked to endocrine changes, aging, nutritional deficiencies, inflammation, reduced mechanical loading, and genetic susceptibility, increasing evidence suggests that the gut microbiota may also contribute to the regulation of bone metabolism [2,3].

The gut–bone axis has emerged as a biologically plausible framework that links intestinal microbial communities to skeletal homeostasis. Gut bacteria influence bone remodeling through several mechanisms, including regulation of intestinal barrier integrity, mineral absorption, immune responses, systemic inflammation, and microbial metabolite production [2,3]. Under eubiotic conditions, the gut microbiota supports metabolic and immune balance. In contrast, dysbiosis can increase intestinal permeability and promote inflammatory signaling, which favors osteoclast activation and bone resorption [3,4]. These mechanisms suggest that gut microbial composition and function may be relevant not only as correlates of bone health, but also as potential contributors to osteoporosis-related phenotypes.

Multiple microbial-derived metabolites and bacterial structural components have been proposed as mediators of the gut–bone axis. Metabolically, short-chain fatty acids (SCFAs), mainly acetate, propionate, and butyrate, are generated through bacterial fermentation of dietary carbohydrates and are associated with immune regulation, intestinal barrier maintenance, and modulation of osteoclastogenesis [5]. In addition, microbial metabolic products, such as tryptophan-derived indoles, vitamin K2/menaquinone, folate, vitamin B12, bile acid derivatives, and polyamines, may influence bone turnover by affecting inflammation, osteoblast activity, osteocalcin carboxylation, energy metabolism, and host–microbiome signaling [3,6]. Furthermore, bacterial immunogenic components such as lipopolysaccharide (LPS), flagellin, and peptidoglycan can activate innate immune pathways and contribute to inflammatory bone loss when intestinal barrier function is compromised [4]. Bacterial extracellular vesicles (BEVs) represent an additional mechanism of microbiota–bone communication. Experimental studies indicate that gut microbiota-derived extracellular vesicles can reach bone tissue and modulate both osteogenic and osteoclastogenic activity. Disruption of beneficial BEV-mediated signaling has been linked to bone pathology [7,8]. Recent research has explored bioengineered BEVs as targeted carriers of osteoclastogenesis-inhibitory molecules, highlighting their potential relevance to bone remodeling and osteoporosis-focused therapeutic strategies [9].

Human studies have reported differences in gut microbial profiles between individuals with normal bone mass and those with osteopenia or osteoporosis, particularly in postmenopausal populations [10,11]. However, the specific taxa associated with low bone mineral density vary across studies, likely due to differences in age, sex, diet, ethnicity, sequencing platforms, skeletal sites evaluated, and analytical methods. This variability highlights the need to move beyond taxonomic descriptions and adopt functional genomic approaches that can capture shared metabolic capacities among osteoporosis-associated bacteria.

In silico genome mining provides a useful strategy to explore the predicted functional potential encoded in bacterial reference genomes, rather than directly measuring microbial activity in human samples. General annotation tools such as Prokka, eggNOG-mapper, and KofamKOALA enable standardized identification of coding sequences, orthologous groups, and metabolic pathways [12,13,14]. Additionally, specialized platforms such as gutSMASH, antiSMASH, and dbCAN/run_dbCAN can identify metabolic gene clusters, biosynthetic gene clusters, and carbohydrate-active enzymes, respectively, providing complementary insights into bacterial metabolic capabilities [15,16,17]. Integrating these approaches may help prioritize microbial functions related to short-chain fatty acid production, menaquinone biosynthesis, tryptophan metabolism, vitamin metabolism, bile acid-associated pathways, carbohydrate degradation, and inflammatory bacterial components.

Despite growing interest in the gut microbiota–bone relationship, limited information is available regarding the genome-encoded functional repertoire of bacteria associated with osteoporosis or low bone mineral density. This study, therefore, aimed to perform in silico functional profiling of osteoporosis-associated gut bacteria, focusing on the predicted functional potential of curated reference genomes, including metabolic and biosynthetic gene clusters, carbohydrate-active enzymes, and bone-relevant pathways linked to the gut–bone axis. This approach may help identify candidate bacterial functions and metabolites involved in intestinal barrier regulation, inflammation, and bone remodeling, thereby providing a mechanistic, hypothesis-generating framework for future experimental and translational research.

## 2. Materials and Methods

### 2.1. Study Design

A comparative in silico functional profiling analysis was conducted on gut bacterial taxa previously associated with osteoporosis, low bone mineral density (BMD), normal BMD, or context-dependent bone-related phenotypes. The analytical workflow integrated literature-informed taxon selection, retrieval of representative genomes, genome-wide functional annotation, mapping of curated gut–bone axis signatures, carbohydrate-active enzyme profiling, selected KEGG pathway analysis, gutSMASH-based prediction of metabolic gene clusters, and integrated functional scoring.

### 2.2. Selection of Candidate Gut Bacterial Taxa

Candidate bacterial taxa were selected from published human microbiome studies that reported associations with osteoporosis, low BMD, osteopenia, normal BMD, or bone turnover-related phenotypes [10,11,18,19,20,21]. Taxa were classified into four analytical roles: main osteoporosis/low-BMD candidates, secondary osteoporosis-associated candidates, normal-BMD-associated comparators, and context-dependent functional comparators. The justification for each taxon, its association category, and the principal supporting reference are summarized in Table 1. Inclusion criteria prioritized taxa with statistically supported associations in human studies and sufficient taxonomic resolution or biological plausibility for genome-level analysis. When evidence was available only at the genus level, representative species were selected for genome mining based on biological relevance, availability of high-quality genome assemblies, and suitability for comparative functional analysis. The final taxon-level selection included 20 candidate taxa, represented by 26 bacterial genomes.

### 2.3. Representative Genome Retrieval and Curation

Representative bacterial genomes were retrieved from the NCBI RefSeq database using the assembly accessions listed in Table S1. The 26 genomes, along with their RefSeq accession numbers, assembly-level information, and quality criteria, are detailed in Table S1. Complete genome assemblies were prioritized when available. Genome metadata included organism name, assembly accession, database source, assembly level, genome size, number of contigs, N50, GC content, material type when available, and inclusion status. For taxa reported at the genus level, species or strain representatives were selected based on genome availability, assembly quality, and suitability as reproducible references for comparative genome mining. These genomes serve as representative references rather than exhaustive descriptions of the functional diversity within a genus. Genomes were retained if they met the assembly quality threshold for comparative functional profiling. The final panel comprised 26 bacterial genomes, representing main osteoporosis/low-BMD candidates, secondary osteoporosis-associated candidates, normal-BMD-associated comparators, and one context-dependent comparator. Protein-coding sequences from each genome were utilized for subsequent functional annotation. Genome-level annotation summaries documented the number of annotated proteins and the diversity of functional identifiers, including Clusters of Orthologous Groups (COG) categories, KEGG orthologs, KEGG pathways, KEGG modules, EC numbers, CAZy annotations, PFAM domains, and Gene Ontology terms.

### 2.4. Genome-Wide Functional Annotation

Genome-wide functional annotation was performed using an orthology-based annotation strategy centered on eggNOG-mapper outputs. The same annotation workflow was applied to all genomes to ensure comparability across the panel. Annotated protein tables were parsed to extract KEGG orthologs, EC numbers, COG categories, KEGG pathways, KEGG modules, CAZy annotations, PFAM domains, Gene Ontology terms, preferred gene names, and functional descriptions. These annotation outputs were used to summarize genome-level functional repertoires and identify proteins that match curated gut–bone axis functional signatures. The number of annotated proteins per genome was used as the denominator in normalized functional comparisons. All normalized burdens were expressed as hits per 1000 annotated proteins to minimize bias related to genome size or differences in the total number of predicted proteins.

### 2.5. Curated Gut–Bone Axis Functional Signature Framework

A curated functional signature framework was developed to capture microbial functions potentially relevant to the gut–bone axis. Twelve functional signatures were evaluated: butyrate-related metabolism, acetate-related metabolism, propionate-related metabolism, vitamin K/menaquinone biosynthesis, vitamin B12/cobalamin metabolism, folate/one-carbon metabolism, tryptophan/indole metabolism, bile acid-related functions, polyamine metabolism, LPS/lipid A biosynthesis, flagellar machinery/Toll-like receptor 5 (TLR5)-related features, and peptidoglycan/cell-wall biosynthesis. Functional signatures were defined using curated combinations of KEGG orthologs, EC numbers, preferred gene names, and functional descriptions derived from KEGG-based pathway information, previous gut–bone axis literature, and manual biological review. The final set of 12 signatures was limited to functions with plausible mechanistic links to microbial metabolism, intestinal barrier regulation, immune signaling, or bone remodeling. Candidate protein hits were assigned to a given signature when their annotation matched one or more curated identifiers for that functional category. Feature-level evidence was organized by functional signature, feature identifier, preferred name, KEGG ortholog, EC number, evidence tier, number of genomes detected, number of proteins detected, and representative functional description.

To avoid overinterpretation of broad annotation matches, two distinct levels of curation were implemented. The primary candidate-hit universe comprised all proteins that matched the curated gut–bone axis feature framework and served as the basis for the main genome-by-signature matrices, normalized burden calculations, primary figures, and integrated analyses. A more stringent high-confidence subset was generated to assess annotation robustness, applying more conservative feature-retention criteria. This strict subset was utilized for curation-impact assessment and for a secondary sensitivity analysis of genome-level normalized burden and metabolic–immunogenic profile classification. However, it did not replace the primary candidate-hit matrix used in the main analyses.

### 2.6. Genome-by-Signature Matrices

For each bacterial genome, candidate protein hits were counted across the 12 curated gut–bone axis signatures. These counts were used to generate a genome-by-signature count matrix. A corresponding presence/absence matrix was also generated by converting each functional signature to a binary status based on detection of at least one candidate hit in a given genome. Global signature summaries included the number of genomes in which each signature was detected, the number of candidate proteins assigned to each signature, and the number of unique KEGG orthologs and EC numbers contributing to each signature. These outputs were used to generate the main global functional signature table and the primary gut–bone axis heatmap.

### 2.7. Curation-Impact and Strict-Subset Sensitivity Analysis

The impact of curation was evaluated by comparing the primary candidate-hit universe with the strict high-confidence subset for each functional signature. For each signature, the following metrics were calculated: number of primary candidate hits, strict high-confidence hits, non-retained hits, retention percentage, primary genomes detected, and strict-subset genomes detected. The overall retention percentage was defined as the ratio of the total number of strict high-confidence hits to the total number of primary candidate hits. To determine the sensitivity of genome-level conclusions to curation stringency, the 2497 strict high-confidence hits were aggregated by genome and functional signature. Total, metabolic, and immunogenic burdens were recalculated per 1000 annotated proteins, using the same denominators and classification framework as in the primary analysis. Functional-profile thresholds were recalculated within the strict subset. Ranking stability between the primary and strict analyses was evaluated using Spearman rank correlation and overlap among the four highest-ranked genomes. Profile stability was summarized as the proportion of genomes retaining the same metabolic–immunogenic classification.

### 2.8. Normalized Gut–Bone Axis Burden and Functional Profile Classification

To enable comparison of bacterial genomes with different annotation depths, total curated gut–bone axis hits were normalized by the number of annotated proteins and expressed as hits per 1000 annotated proteins. Functional hits were further grouped into metabolic and immunogenic components. Metabolic hits included SCFA-related metabolism, vitamin/cofactor metabolism, tryptophan/indole metabolism, bile acid-related functions, and polyamine metabolism. Immunogenic or structurally immune-relevant hits included LPS/lipid A biosynthesis, flagellar machinery/TLR5-related features, and peptidoglycan/cell-wall biosynthesis. Metabolic hits per 1000 annotated proteins, immunogenic hits per 1000 annotated proteins, and the metabolic-to-immunogenic ratio were calculated for each genome. Genomes were then categorized into functional profile groups according to their normalized burden and the relative distribution of metabolic and immunogenic signatures. Genomes exceeding the third-quartile threshold for both metabolic and immunogenic burden were designated as mixed high-burden profiles. Those exceeding the third-quartile threshold for only one axis were classified as either metabolic-enriched or immunogenic-enriched. Genomes at or above the median for both axes, but below the third-quartile thresholds, were assigned to the balanced moderate-high profiles group. The remaining genomes were categorized as having a lower/intermediate functional burden.

### 2.9. CAZyme Repertoire Analysis

Carbohydrate-active enzyme (CAZyme) repertoires were analyzed using CAZy annotations extracted from genome-wide functional annotation outputs. CAZyme hits were summarized by genome and by major CAZyme class, including glycoside hydrolases, glycosyltransferases, carbohydrate esterases, carbohydrate-binding modules, polysaccharide lyases, and auxiliary activities. For each genome, total CAZy hits, unique CAZy families, and class-specific hit counts were calculated. To account for differences in genome annotation depth, CAZyme class burdens were normalized per 1,000 annotated proteins. CAZyme diversity and normalized CAZyme burden were compared across genomes to identify bacteria with high carbohydrate degradation potential, high CAZyme family diversity, or class-specific enrichment patterns.

### 2.10. Selected KEGG Pathway Analysis

A focused KEGG pathway analysis was conducted using a curated set of gut–bone axis-relevant or bone-related pathways. The 31 selected pathways were prioritized because they represented central microbial metabolism, carbohydrate utilization, amino-acid and vitamin/cofactor metabolism, transport systems, microbial signaling, cell-envelope-related processes, and SCFA-related routes plausibly relevant to host–microbe interactions or skeletal homeostasis. For each selected KEGG pathway, raw pathway hits were counted across genomes. Global pathway summaries included the total raw hits, the number of genomes in which each pathway was detected, and the mean, median, and maximum normalized burden. Genome-level KEGG pathway burden was calculated as the total number of selected KEGG pathway hits normalized per 1000 annotated proteins. These values were used to compare pathway-level functional potential across the bacterial panel.

### 2.11. gutSMASH Metabolic Gene Cluster Analysis

Metabolic gene clusters (MGCs) were predicted using gutSMASH. For each genome, predicted protoclusters were cataloged by bacterial species, analysis role, genome accession, record identifier, protocluster number, predicted gutSMASH product, product category, genomic location, coding sequence count, and defining domains. Predicted products were manually grouped into broader categories, including fermentation/energy metabolism, amino acid metabolism, redox/nitrogen metabolism, aromatic/phenolic metabolism, lipid/fatty acid metabolism, polyamine metabolism, sulfur metabolism, and other or unassigned products. Exact gutSMASH product labels containing underscores were retained when they represented direct software output strings.

Genome-level gutSMASH summaries included the total number of predicted metabolic gene clusters, the number of unique predicted products, normalized metabolic gene cluster burden per 1000 annotated proteins, mean coding sequences per cluster, top predicted gutSMASH products, and top product categories. Additional summaries were generated by product type, analysis role, and product-category matrix to evaluate differences in metabolic gene cluster repertoires between osteoporosis-associated and comparator genomes.

### 2.12. Integrated Functional Scoring and Sensitivity Analysis

An integrated functional score was developed to summarize the multidimensional functional potential across bacterial genomes. This integrated framework incorporated normalized gut–bone axis burden, metabolic burden, immunogenic burden, CAZyme burden, selected KEGG pathway burden, gutSMASH MGC burden, number of unique gutSMASH products, and selected MGC product-category features. Component-level metrics were standardized prior to aggregation to enable comparison across functional dimensions and to prevent metrics with larger numerical ranges from dominating the score. An equal-weight prioritization strategy was implemented a priori, as no experimentally validated coefficients or biological effect-size estimates are currently available to justify preferential weighting of individual functional dimensions. Equal weighting therefore provided a transparent and minimally assumption-dependent approach for comparative prioritization. Importantly, the resulting score was intended as a relative ranking of predicted functional potential within the analyzed genome panel rather than as a quantitative estimate of biological effect magnitude.

Two complementary integrated scores were calculated: an integrated metabolic/MGC score and an integrated immunogenic score. These scores were combined to generate a global integrated functional score. Genomes were classified into integrated profile classes based on these values and the relative enrichment of specific functional axes. The classes included low integrated functional potential, intermediate integrated profile, metabolic/MGC-enriched integrated profile, immunogenic-pattern-enriched integrated profile, mixed high metabolic–immunogenic potential, and high global integrated potential. Sensitivity analyses were conducted to assess the stability of genome rankings and to determine the extent to which the global score depended on individual components or weighting assumptions. In addition to rankings based on individual functional metrics, an explicit equal-weight reference was calculated as the arithmetic mean of the z-standardized normalized gut–bone burden, metabolic burden, immunogenic burden, CAZyme burden, KEGG burden, MGC burden, unique MGC products, fermentation/energy MGCs, amino acid MGCs, and redox/nitrogen MGCs. Leave-one-component-out analyses were performed by sequentially excluding each component and recalculating the integrated score using equal weights for the remaining nine components. Alternative weighting scenarios were evaluated by assigning 20% of the total weight to each component individually and 8.89% to each of the remaining nine components. Ranking stability relative to the equal-weight reference was evaluated using Spearman rank correlations and top-five overlap. Correlations between the global integrated score and individual component metrics were also calculated to characterize the contribution of each functional dimension.

### 2.13. Data Visualization

Figures were generated to summarize global and genome-level patterns across the bacterial panel. Heatmaps visualized genome-by-signature matrices, CAZyme-class distributions, selected KEGG pathway representations, gutSMASH product-category composition, and integrated multidimensional scores. Bar plots displayed global signature counts, normalized gut–bone axis signal, top CAZyme and KEGG pathway categories, and metabolic gene cluster counts. Scatter plots compared normalized metabolic and immunogenic gut–bone axis signals and evaluated the relationship between CAZyme diversity and normalized CAZyme density. Bacterial names and functional categories were harmonized across figures and tables. The main figures present the curated gut–bone axis signature landscape and the normalized genome-level classification, whereas the supplementary figures display CAZyme repertoires, selected KEGG pathway patterns, gutSMASH metabolic gene clusters, and integrated multidimensional analyses.

### 2.14. Software, Databases, and Reproducibility

Genome assemblies were retrieved from the NCBI RefSeq database using the assembly accessions listed in Supplementary Table S1. RefSeq genomes corresponded to RefSeq Release 235 and were accessed/downloaded on 17 June 2026. Orthology-based functional annotation was performed using eggNOG-mapper v2.1.14 against the current eggNOG database release available at the time of analysis. Metabolic gene clusters were predicted with gutSMASH v2.0.1 using Prokka-derived GenBank files as input. Carbohydrate-active enzyme annotation, when applicable, was performed using dbCAN/run_dbCAN v5.2.9 with dbCAN HMMdb v14, based on CAZyDB 10 July 2025; the CAZy database was additionally consulted using its June 2026 update for manual family-level interpretation. Downstream parsing, matrix construction, normalization, statistical summaries, and visualization were performed with custom scripts in R v4.6.0 and Python v3.14.6.

## 3. Results

### 3.1. Selection of Osteoporosis-Associated and Comparator Gut Bacterial Taxa

A total of 20 gut bacterial taxa were selected for comparative in silico functional profiling, based on published human evidence associating them to osteoporosis, low bone mineral density (BMD), normal BMD, or context-dependent bone-related phenotypes (Table 1). The selection comprised ten main osteoporosis/low-BMD candidates, three secondary osteoporosis-associated candidates, six normal-BMD-associated comparators, and one context-dependent functional comparator. The main osteoporosis/low-BMD candidate group included *Bacteroides* spp., *Parabacteroides* spp., *Barnesiella* spp., *Odoribacter* spp., *Sutterella* spp., *Butyricimonas* spp., *Coprobacter* spp., *Oscillibacter* spp., *Dialister* spp., and *Eggerthella* spp. Secondary candidates were *Actinomyces* spp., *Turicibacter* spp., and *Catenibacterium* spp., whereas normal-BMD-associated comparators included *Agathobacter* spp., *Coprococcus* spp., *Dorea* spp., the *Ruminococcus torques* group, *Megamonas* spp., and *Roseburia intestinalis*. *Faecalibacterium* spp. was used as a context-dependent functional comparator.

To address the reporting of several taxa were described at the genus level in the human microbiome literature, the candidate list was converted into 26 representative bacterial genomes for genome mining (Table S1). The final genome set comprised 12 main osteoporosis/low-BMD candidate genomes, 4 secondary osteoporosis-associated genomes, 9 normal-BMD-associated comparator genomes, and 1 context-dependent functional comparator genome. Most assemblies represented complete genomes, with only one scaffold-level assembly. Genome sizes ranged from 1.90 to 5.73 Mb (median size of 3.32 Mb), and the number of contigs ranged from 1 to 3, supporting that these genomes are suitable for comparative functional annotation.

### 3.2. Curated Gut–Bone Axis Functional Signatures Across Candidate Genomes

Analysis of the 26 representative genomes identified 12 predicted genome-encoded gut–bone axis functional signatures, comprising 3337 primary candidate protein hits, 524 unique KEGG orthologs, and 279 unique EC numbers (Table 2). The most abundant signatures included vitamin B12/cobalamin metabolism (690 candidate proteins across all genomes), folate/one-carbon metabolism (553 candidate proteins), peptidoglycan/cell-wall biosynthesis (520 candidate proteins), and butyrate-related metabolism (382 candidate proteins). Additional widely distributed metabolic signatures encompassed propionate-related metabolism, tryptophan/indole metabolism, polyamine metabolism, acetate-related metabolism, bile acid-related functions, and vitamin K/menaquinone biosynthesis. Structural or immune-relevant microbial signatures, such as LPS/lipid A biosynthesis and flagellar machinery/TLR5-related features, were also detected. The universal presence of vitamin B12/cobalamin and folate/one-carbon metabolism was interpreted as indicative of both bone-relevant cofactor activity and a broadly conserved bacterial metabolic function.

**Figure 1:**
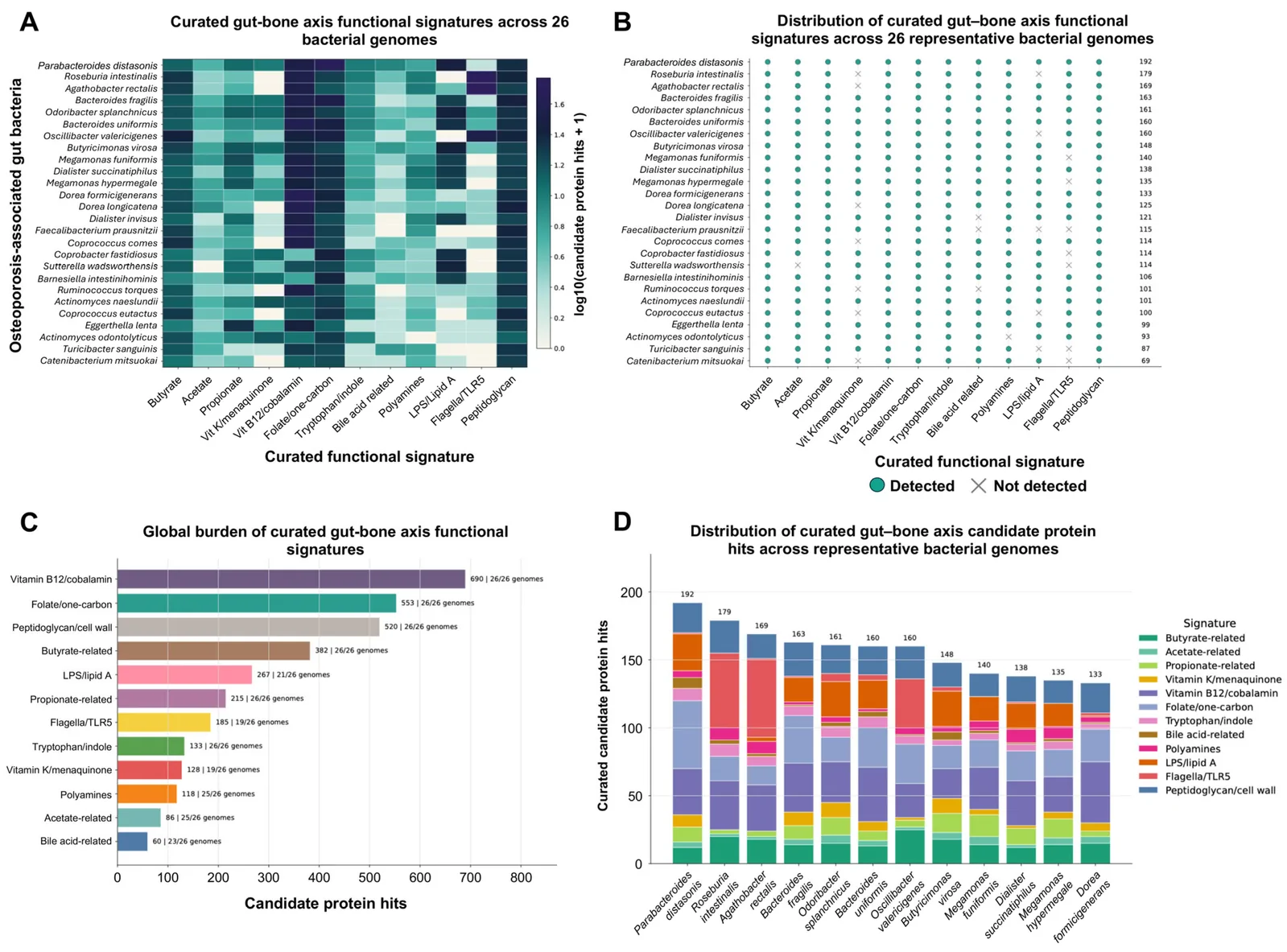
Curated gut–bone axis functional signatures across 26 representative bacterial genomes. (**A**) Heatmap showing the distribution of curated gut–bone axis candidate protein hits across the 26 bacterial genomes. Values are presented as log10 (candidate protein hits + 1) for each genome and functional signature. (**B**) Presence/absence matrix of the 12 curated functional signatures across genomes; filled circles indicate detected signatures, while crosses denote non-detected signatures. Numbers on the right indicate the total number of candidate protein hits per genome. (**C**) Global burden of curated gut–bone axis functional signatures across the full genome panel, expressed as total candidate protein hits and the number of genomes in which each signature was detected. (**D**) Stacked bar plot depicting the bacterial genomes with the highest total curated gut–bone axis burden, stratified by functional signature. Dominant signatures comprised vitamin B12/cobalamin metabolism, folate/one-carbon metabolism, peptidoglycan/cell-wall biosynthesis, and butyrate-related metabolism.

Analysis of the genome-by-signature landscape showed that most bacterial genomes encoded multiple functions relevant to the gut–bone axis. However, the magnitude and composition of these signatures varied considerably among species (Figure 1A). The presence/absence summary demonstrated that butyrate, propionate, vitamin B12/cobalamin, folate/one-carbon metabolism, tryptophan/indole metabolism, and peptidoglycan/cell-wall biosynthesis were present in all 26 genomes. In contrast, vitamin K/menaquinone biosynthesis and flagellar machinery/TLR5-related features showed more limited distributions (Figure 1B). The global burden plot identified vitamin B12/cobalamin metabolism, folate/one-carbon metabolism, peptidoglycan/cell-wall biosynthesis, and butyrate-related metabolism as the predominant functional categories (Figure 1C). At the species level, *Parabacteroides distasonis*, *Roseburia intestinalis*, *Agathobacter rectalis*, *Bacteroides fragilis*, *Odoribacter splanchnicus*, *Bacteroides uniformis*, and *Oscillibacter valericigenes* exhibited the highest absolute gut–bone axis burdens (Figure 1D).

Feature-level curation resulted in 120 curated feature entries supporting the selected gut–bone axis signatures (Table S2). Folate/one-carbon metabolism and flagellar/TLR5-related features represented the largest proportion of curated entries, followed by LPS/lipid A biosynthesis and bile acid-related functions. The majority of curated entries were classified as Tier 1 evidence, indicating reliance on high-confidence functional markers within the annotation framework. Frequently identified curated features included folC, glyA, folD, thyA, folP, lpxA, cbh, purN, and ygfA.

The corrected curation-impact analysis distinguished the primary candidate annotation universe from a more conservative, strict high-confidence subset (Table S3). Of the 3337 primary candidate hits used in the main genome-by-signature analyses, 2497 were retained in the strict high-confidence subset, representing an overall retention rate of 74.83%. Complete retention was observed for propionate-related metabolism, tryptophan/indole metabolism, vitamin K/menaquinone biosynthesis, and polyamine metabolism. High retention rates were also noted for peptidoglycan/cell-wall biosynthesis and LPS/lipid A biosynthesis. In contrast, retention rates were lower for acetate-related metabolism, bile acid-related functions, and particularly butyrate-related metabolism, which reflects the implementation of more stringent evidence requirements for specific functional markers. This strict subset was used to evaluate annotation robustness and, as a secondary sensitivity analysis, to determine whether genome-level normalized rankings and metabolic–immunogenic profile classifications remained stable under more conservative feature retention criteria.

The genome-by-signature count matrix showed substantial variation in absolute functional burdens among genomes (Table S4). *Parabacteroides distasonis*, *Roseburia intestinalis*, *Agathobacter rectalis*, *Bacteroides fragilis*, *Odoribacter splanchnicus*, *Bacteroides uniformis*, and *Oscillibacter valericigenes* exhibited the highest total curated hit counts. The corresponding presence/absence matrix indicated that six functional signatures were present in all 26 genomes. Polyamine and acetate-related signatures were identified in 25 genomes, bile acid-related functions in 23 genomes, LPS/lipid A in 21 genomes, and vitamin K/menaquinone and flagellar/TLR5-related features in 19 genomes.

### 3.3. Genome-Wide Annotation and Normalized Gut–Bone Axis Burden

Genome-wide eggNOG annotation identified between 1613 and 4623 annotated proteins per genome (Table S5). Parabacteroides distasonis, Bacteroides fragilis, Bacteroides uniformis, *Oscillibacter valericigenes*, and *Roseburia intestinalis* showed the highest numbers of annotated proteins. Across all genomes, the number of unique KEGG orthologs ranged from 1196 to 1810, unique EC numbers from 554 to 885, unique CAZy annotations from 13 to 47, and unique Gene Ontology terms from 2235 to 3821.

The genome-wide annotation outputs served as the basis for normalizing functional burdens according to annotated protein content.

**Figure 2:**
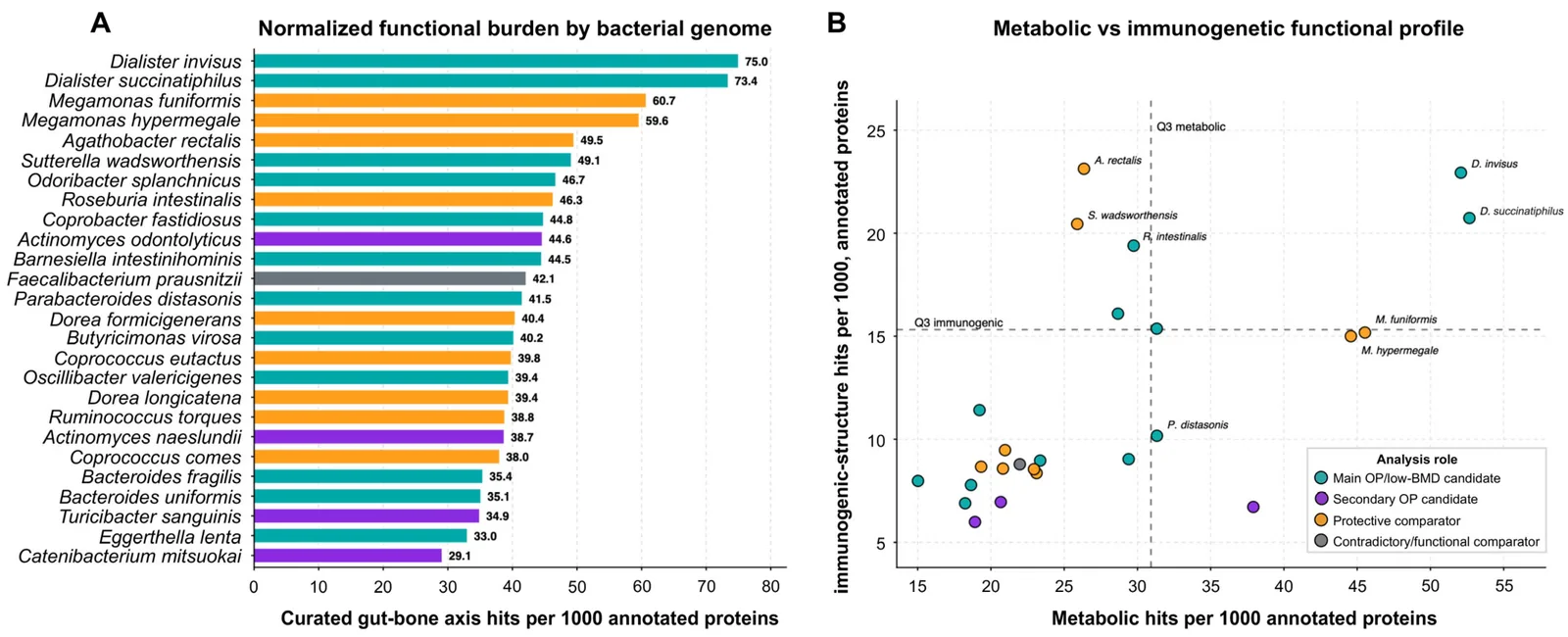
Normalized gut–bone axis functional burden and classification of metabolic–immunogenic profiles across bacterial genomes. (**A**) Bar plot showing the normalized burden of curated gut–bone axis candidate protein hits across the 26 representative bacterial genomes, expressed as hits per 1000 annotated proteins. Colors indicate the analytical role assigned to each genome: main osteoporosis/low-BMD candidate, secondary osteoporosis-associated candidate, normal-BMD-associated comparator, or contradictory/functional comparator. (**B**) Scatter plot comparing normalized metabolic and immunogenic or structural gut–bone axis burdens. Dashed lines represent third-quartile thresholds used to identify genomes with elevated metabolic or immunogenic burdens. *Dialister invisus* and *Dialister succinatiphilus* exhibited mixed high-burden profiles, while *Megamonas funiformis* and *Megamonas hypermegale* demonstrated greater metabolic enrichment. Several genomes, including *Agathobacter rectalis*, *Roseburia intestinalis*, and *Sutterella wadsworthensis*, displayed relatively higher immunogenic or structural burdens.

Following normalization, *Dialister invisus* and *Dialister succinatiphilus* showed the highest gut–bone axis burdens, with 75.02 and 73.40 curated hits per 1000 annotated proteins, respectively (Table S6; Figure 2A). The normal-BMD-associated comparators *Megamonas funiformis* and *Megamonas hypermegale*, ranked next with 60.71 and 59.58 hits per 1000 annotated proteins, respectively. Additional genomes with relatively high normalized burdens included *Agathobacter rectalis*, *Sutterella wadsworthensis*, *Odoribacter splanchnicus*, *Roseburia intestinalis*, *Coprobacter fastidiosus*, and *Actinomyces odontolyticus*. Normalization process changed the interpretation of genome rankings compared to absolute hit counts, highlighting taxa such as *Dialister* spp., whose predicted gut–bone axis signal remained proportionally elevated when expressed relative to annotated protein content.

The metabolic-versus-immunogenic profile analysis distinguished genomes based on their normalized metabolic and structural immunogenic burdens (Figure 2B; Table S6). *Dialister invisus* and *Dialister succinatiphilus* showed high normalized burdens in both axes and were classified as mixed high-burden profiles. *Megamonas funiformis*, *Megamonas hypermegale*, and the context-dependent comparator *Faecalibacterium prausnitzii* were categorized as metabolic-enriched profiles. In contrast, *Agathobacter rectalis*, *Sutterella wadsworthensis*, *Odoribacter splanchnicus*, *Roseburia intestinalis*, and *Coprobacter fastidiosus* were identified as immunogenic-enriched profiles. The largest category identified was the lower or intermediate functional burden group, followed by immunogenic-enriched, metabolic-enriched, mixed high-burden, and balanced moderate–high profiles. Reanalysis restricted to the 2497 strict high-confidence hits showed strong concordance with the primary normalized burden ranking (Spearman = 0.958, *p* < 0.001; Figure S6; Table S6C). The four highest-ranked genomes remain unchanged: *Dialister invisus* and *Dialister succinatiphilus* retained the first and second positions, respectively, while *Megamonas hypermegale* and *Megamonas funiformis*, exchanged their relative order compared to the primary analysis. Their strict normalized burdens were 62.00, 59.57, 46.34, and 45.97 hits per 1000 annotated proteins, respectively. Metabolic–immunogenic profile classifications were consistent for 22 of 26 genomes (84.6%). Notably, both *Dialister species* remained classified as mixed high-burden profiles, and both *Megamonas species* remained metabolic-enriched. Only four genomes changed profile classification under strict curation, supporting the robustness of the central taxon prioritization and the main conclusions of Figure 2 to more conservative feature selection.

### 3.4. CAZyme Repertoire of Osteoporosis-Associated and Comparator Genomes

A total of 1896 CAZy hits were identified across the 26 genomes analyzed (Table S7). Glycoside hydrolases represented the most abundant CAZyme class, accounting for 1018 hits, followed by glycosyltransferases with 760 hits. Carbohydrate-binding modules, carbohydrate esterases, polysaccharide lyases, and auxiliary activities were present at lower frequencies. The greatest absolute CAZyme burdens were found in *Bacteroides uniformis* (183 CAZy hits, and 47 unique CAZy *families*), *Roseburia intestinalis* (145 hits), *Bacteroides fragilis* (143 hits), and *Parabacteroides distasonis* (131 hits).

Normalization by annotated protein content revealed the highest CAZyme burdens in *Barnesiella intestinihominis*, *Coprobacter fastidiosus*, *Bacteroides uniformis*, and *Roseburia intestinalis* (Figure S1A; Table S7). At the class-level, glycoside hydrolases and glycosyltransferases accounted for the majority CAZyme annotations among top-ranked genomes (Figure S1B). Analysis of CAZyme diversity and normalized burden showed that certain genomes exhibited both high family diversity and high normalized CAZyme density, whereas others displayed moderate diversity with lower normalized burdens (Figure S1C). The most frequently observed CAZy families across genomes were GT2, GH2, GH13, GH31, GH3, GH5, CBM48, and GT51 (Figure S1D). The normalized CAZyme-class heatmap corroborated the predominance of GH and GT profiles. It highlighted *Barnesiella intestinihominis*, *Coprobacter fastidiosus*, *Bacteroides uniformis*, *Roseburia intestinalis*, and *Catenibacterium mitsuokai* as genomes with relatively high carbohydrate-active enzyme burdens (Figure S2).

### 3.5. Selected KEGG Pathway Burden

The KEGG pathway analysis focused on 31 pathways relevant to the gut–bone axis or bone-related metabolic, transport, signaling, and structural functions (Table S8). Collectively, these pathways accounted for 24,156 raw pathway hits across the 26 genomes. The highest global burdens were associated with biosynthesis of amino acids, ABC transporters, carbon metabolism, two-component systems, quorum sensing, and amino sugar/nucleotide sugar metabolism. Additional broadly represented pathways included glycolysis/gluconeogenesis, pyruvate metabolism, cysteine and methionine metabolism, galactose metabolism, glycine/serine/threonine metabolism, fructose and mannose metabolism, peptidoglycan biosynthesis, and the pentose phosphate pathway.

Most of the selected KEGG pathways were widely distributed, with 25 out of 31 pathways detected in all 26 genomes (Table S8). Normalized KEGG pathway burdens varied, with the highest values observed in *Megamonas hypermegale*, *Sutterella wadsworthensis*, *Megamonas funiformis*, *Dialister succinatiphilus*, and *Dialister invisus*. The genome-by-pathway heatmap showed broad representation of central metabolic, transport, signaling, carbohydrate-related, and cell-envelope-associated pathways across the bacterial panel (Figure S3A). The global KEGG pathway summary further confirmed the predominance of amino acid biosynthesis, ABC transporters, carbon metabolism, two-component systems, and quorum sensing among the selected pathways (Figure S3B).

### 3.6. gutSMASH-Predicted Metabolic Gene Clusters

The gutSMASH analysis identified 291 predicted metabolic gene clusters or protoclusters across the 26 genomes (Table S9). These clusters were categorized into eight product types. Fermentation/energy metabolism represented the dominant category, comprising 136 clusters, followed by amino acid metabolism, redox/nitrogen metabolism, aromatic/phenolic metabolism, lipid/fatty acid metabolism, other or unassigned products, polyamine metabolism, and sulfur metabolism. The most frequently predicted products included Others_HGD_unassigned, Rnf_complex, succinate2propionate, Fumarate2succinate, PFOR_II_pathway, Pyruvate2acetate-formate, TPP_AA_metabolism, Phenylpyruvate_ferredoxin_oxidoreductase, xanthine_dehydrogenase, and Molybdopterin_dependent_oxidoreductase. These labels were retained as direct gutSMASH output terms to ensure traceability to the software results.

Genome-level gutSMASH summaries showed that *Eggerthella lenta* had the highest metabolic gene cluster burden, with 24 predicted MGCs, followed by *Oscillibacter valericigenes* with 23 MGCs (Table S10). Additional genomes with high MGC counts included *Coprococcus comes*, *Dorea longicatena*, and *Dorea formicigenerans*, each with 16 MGCs, and *Odoribacter splanchnicus* with 15 MGCs. After normalization by annotated proteins count, *Eggerthella lenta* remained the highest-ranking genome, followed by *Oscillibacter valericigenes*, *Coprococcus comes*, *Megamonas hypermegale*, *Dorea longicatena*, and *Dorea formicigenerans*. The role-level summary showed similar mean MGC counts between primary osteoporosis/low-BMD candidates and normal-BMD-associated comparators, whereas secondary osteoporosis-associated candidates displayed lower mean MGC counts. The product-category matrix confirmed that fermentation/energy metabolism was the most common MGC category across most genomes, with additional contributions from amino acid, redox/nitrogen, aromatic/phenolic, lipid/fatty acid, polyamine, and sulfur metabolism categories.

The genome-level MGC plot highlighted *Eggerthella lenta* and *Oscillibacter valericigenes* as the genomes with highest-burden of gutSMASH-predicted metabolic gene clusters (Figure S4A). The product-category heatmap confirmed the predominance of fermentation/energy metabolism across the bacterial panel and revealed a marked enrichment of redox/nitrogen metabolism in *Eggerthella lenta* and lipid/fatty-acid metabolism in *Oscillibacter valericigenes* (Figure S4B).

### 3.7. Sensitivity Analysis and Integrated Functional Classification

Sensitivity analysis showed that the global integrated score was most strongly associated with MGC burden (Spearman rho = 0.768, Table S11). Correlations with the number of detected signatures, metabolic burden, normalized gut–bone axis burden, KEGG burden, absolute gut–bone hits, and immunogenic burden were comparatively weaker. In contrast, CAZyme burden displayed a negative correlation with the global integrated score. Analysis of the top-five overlap showed that MGC burden shared three of the top five genomes with the global integrated ranking. Normalized gut–bone burden and KEGG burden each shared two, CAZyme burden shared one, and absolute gut–bone hits shared none. To further evaluate the robustness of the weighting strategy, an explicit equal-weight reconstruction showed high concordance with the reported global integrated ranking (Spearman = 0.973, *p* < 0.001; top-five overlap = 4/5). Leave-one-component-out analyses also indicated consistently high agreement with the equal-weight reference (Spearman = 0.905–0.971, all *p* < 0.001), with top-five overlaps ranging from 3/5 to 5/5. Similarly, alternative weighting scenarios in which each component was individually increased to 20% yielded correlations of Spearman = 0.904–0.992 (*p* < 0.001) and top-five overlaps of 3/5 to 5/5. Together, these analyses indicate that the overall comparative ranking was robust to omission or moderate reweighting of individual functional components, although the ordering of some closely ranked genomes varied across scenarios.

The integrated functional classification identified six distinct profile classes among the 26 genomes analyzed (Table S12). The intermediate integrated profile was the most common, followed by profiles characterized by low integrated functional potential, metabolic/MGC-enrichment, high global integrated potential, immunogenic-pattern enrichment, and a mixed high metabolic–immunogenic potential. The highest global integrated scores were observed in the normal-BMD-associated comparators *Megamonas hypermegale* and *Megamonas funiformis*, both categorized as metabolic/MGC-enriched integrated profiles. Among primary osteoporosis/low-BMD candidates, *Oscillibacter valericigenes* and *Eggerthella lenta* showed high global integrated potential, whereas *Coprobacter fastidiosus* and *Odoribacter splanchnicus* displayed mixed high metabolic–immunogenic potential. Dialister succinatiphilus and Dialister invisus were categorized as immunogenic-pattern-enriched profiles, consistent with their elevated normalized gut–bone axis burden and pronounced structural immunogenic signatures.

The integrated heatmap confirmed that bacterial genomes varied across multiple functional axes, including normalized gut–bone axis burden, metabolic burden, immunogenic burden, CAZyme burden, selected KEGG pathway burden, MGC burden, MGC product diversity, fermentation/energy MGCs, amino acid MGCs, and redox/nitrogen MGCs (Figure S5). *Megamonas hypermegale* and *Megamonas funiformis* showed the highest global integrated scores and broad enrichment across metabolic, KEGG, and MGC-related axes. *Oscillibacter valericigenes* and *Eggerthella lenta* displayed high MGC-driven integrated potential, with *Eggerthella lenta* particularly enriched in redox/nitrogen-related MGCs. In contrast, *Catenibacterium mitsuokai* and *Turicibacter sanguinis* had the lowest global integrated scores, although they retained selected CAZyme and KEGG pathway features. Overall, these findings suggest that osteoporosis-associated and comparator gut bacteria differ not only in the presence of specific gut–bone axis signatures, but also in the relative distribution of metabolic potential, structural immunogenic features, carbohydrate-active enzyme repertoires, KEGG pathway burden, and gutSMASH-predicted metabolic gene clusters.

## 4. Discussion

This study provides a genome-centered view of bacterial functions that may be involved in the gut–bone axis across taxa previously associated with osteoporosis, low BMD, or comparatively preserved bone phenotypes. The main finding is that osteoporosis-associated and comparator bacteria did not differ solely by taxonomic classification; rather, they showed distinct combinations of metabolic, structural immunogenic, carbohydrate-active, KEGG pathway, and gutSMASH-predicted metabolic gene cluster profiles. This functional perspective aligns with experimental evidence indicating that gut microbial communities can regulate bone mass through immune and endocrine mechanisms, as well as with models suggesting that sex steroid deficiency and postmenopausal osteoporosis are partly linked to microbiota-dependent inflammation and barrier dysfunction [22,23,24].

The widespread presence of SCFA-related signatures across the genome panel supports the relevance of microbial fermentation capacity as a shared functional trait rather than one limited to a small subset of taxa. This is significant because acetate, propionate, and butyrate are not solely energy metabolites; they also influence bone remodeling through effects on osteoclast metabolism, immune regulation, and osteoblast-supportive pathways. Experimental studies have demonstrated that SCFAs increase bone mass and protect against pathological bone loss, while butyrate can stimulate bone formation via regulatory T cell-mediated induction of WNT10B expression [25,26]. The finding that several genomes retained butyrate, propionate, and acetate-related signatures also aligns with evidence that the gut microbiota can promote bone formation, in part through IGF-1-dependent mechanisms [27]. Therefore, the SCFA-related results should be interpreted as indicative of potential metabolic capacity that may contribute to bone-relevant host signaling, but not as direct evidence of metabolite production in vivo.

Tryptophan/indole-related signatures were also widely represented, suggesting that candidate taxa may contribute to additional metabolite axis relevant to intestinal barrier regulation, immunemodulation, and osteoclast biology. This is particularly significant because aryl hydrocarbon receptor (AhR) signaling exerts context-dependent effects on osteoclast differentiation and function [28]. Recent experimental evidence further indicates that the gut-derived tryptophan metabolite indoleacrylic acid can suppress osteoclast formation through AhR-mediated NF-kappaB signaling and protect against ovariectomy-induced bone loss in mice [29]. In this context, the presence of tryptophan/indole-related annotations in this analysis supports their inclusion as bone-relevant microbial features. However, the functional impact of these signatures likely depends on the specific metabolite produced, its concentration, and the host inflammatory environment.

A high burden of vitamin B12/cobalamin and folate/one-carbon metabolism signatures across all genomes emerged as one of the most prominent global patterns. These signatures may reflect conserved bacterial requirements for cofactors and nucleotide metabolism. Still, they are also relevant to host bone health because one-carbon metabolism is linked to homocysteine regulation, methylation capacity, and skeletal outcomes. Similarly, the detection of vitamin K/menaquinone biosynthesis in a subset of genomes is notable, as vitamin K-dependent proteins, particularly osteocalcin, require gamma-carboxylation for normal bone matrix function [30]. Systematic reviews further support associations among vitamin B12, folate, homocysteine status, fracture risk, and BMD, although distinguishing causality and the relative contributions of microbial versus dietary sources remain challenging [31]. Therefore, these findings suggest plausible microbial cofactor-related capacities but should be regarded as hypothesis-generating rather than definitive evidence of host vitamin availability.

The immunogenic aspect of the analysis is also significant. Several genomes contained LPS/lipid A, peptidoglycan/cell-wall, and flagellar/TLR5-related features, with some taxa showed relatively high normalized immunogenic burdens. These signatures alone do not imply pathogenicity on their own, because bacterial structural components are common among commensal organisms. However, under conditions of impaired gut barrier integrity, estrogen deficiency, or systemic inflammation, translocation or immune sensing of microbial-associated molecular patterns may contribute to osteoclast activation and bone loss. This interpretation is supported by evidence linking postmenopausal osteoporosis with altered gut microbiota, peripheral immune-cell migration, and increased plasma LPS [24], as well as by experimental data displaying that TLR5 activation by flagellin can promote RANKL expression, osteoclastogenesis, and bone loss [32]. Thus, the balance between metabolic and immunogenic signatures may provide more informative insights than the presence of any single bacterial feature.

The CAZyme and selected KEGG pathway analyses further extend this interpretation by showing that several genomes with high CAZyme burdens also possessed central metabolic and transport pathways that could facilitate fiber utilization and metabolite production. CAZymes are essential for the ability of gut bacteria to degrade complex dietary polysaccharides that the host cannot digest, thereby influencing substrate availability for fermentation and cross-feeding within microbial communities [33]. Human dietary intervention studies further support the idea that fiber intake can alter SCFA production and gut microbiota composition, although responses vary depending on the substrate and individual microbiome configuration [34]. In this study, a high CAZyme burden should be interpreted as an ecological and metabolic potential: it may enhance the capacity to access carbohydrate substrates and support downstream metabolite production, but the resulting metabolite profile would depend on diet, community context, and host physiology. The negative association between CAZyme burden and the global integrated score in the sensitivity analysis may reflect that the integrated score emphasized multiple metabolic and MGC-related dimensions rather than polysaccharide-degrading capacity alone. Therefore, high CAZyme density should be interpreted as a complementary ecological feature, not as a proxy for overall gut–bone axis potential.

The gutSMASH-predicted metabolic gene cluster analysis added further functional resolution. The prominence of fermentation/energy metabolism clusters, along with amino-acid, redox/nitrogen, aromatic/phenolic, lipid/fatty-acid, polyamine, and sulfur-related categories, suggests that osteoporosis-associated and comparator bacteria differ in broader metabolic strategies rather than in isolated pathways. This is particularly notable for species such as *Eggerthella lenta* and *Oscillibacter valericigenes*, which exhibited high MGC burdens, and for *Megamonas genomes*, which ranked highly in the integrated functional score. Within this genome panel, these taxa represent candidates for future targeted evaluation of specialized metabolic capacity, bioactive metabolite production, and host–microbiota interactions relevant to the gut–bone axis. The integrated score provides a comparative prioritization framework, rather than serving as a biological effect-size measure or a direct ranking of taxa as beneficial or harmful. The high concordance observed across leave-one-component-out and alternative-weighting analyses indicates that the overall ranking is robust to the omission or moderate reweighting of individual functional dimensions. However, small differences between closely ranked genomes should be interpreted with caution and in the context of the underlying functional components, as some variation in rank order was observed across sensitivity analyses. These findings indicate that taxonomic classification alone is insufficient to infer bone-relevant microbial behavior. For example, normal-BMD-associated comparator taxa such as *Megamonas funiformis* and *Megamonas hypermegale* exhibited high integrated metabolic/MGC profiles, whereas several osteoporosis-associated candidates displayed mixed or immunogenic-enriched patterns. Notably, a high integrated score does not indicate an adverse or osteoporosis-promoting effect. Instead, it reflects greater and more multidimensional predicted functional potential across the features incorporated into the score. Conversely, some taxa associated with low BMD retained metabolic features that may be beneficial under specific ecological conditions. These results support a model in which osteoporosis-associated dysbiosis reflects an altered balance among metabolic support, immune activation, carbohydrate processing, and specialized primary metabolism, rather than a simple depletion of beneficial taxa or expansion of harmful taxa. This distinction is critical for future microbiome-based interventions, as restoring functional balance may be more effective than targeting individual taxa in isolation.

This study has several strengths. First, it employed a curated, and biologically informed functional framework focused on microbial functions plausibly linked to bone metabolism. Second, it integrated multiple complementary layers, including gut–bone axis signatures, CAZyme repertoires, selected KEGG pathways, and gutSMASH-predicted metabolic gene clusters. Third, the analysis distinguishes the primary candidate-hit universe from a more stringent high-confidence subset, thereby reducing the risk of overinterpreting broad annotation matches. Reanalysis using the strict high-confidence subset retained the four highest-ranked genomes and 84.6% of metabolic–immunogenic profile classifications, indicating that principal taxon prioritization is robust to conservative feature selection. However, several limitations should be acknowledged. Genome mining estimates functional potential but does not measure gene expression, metabolite production, microbial abundance, or host response. While normalization per 1,000 annotated proteins mitigates differences due to genome size and annotation depth, it does not fully separate genuine functional enrichment from effects related to genome architecture or the proportional representation of functional genes. This issue is particularly relevant for smaller genomes, such as those of *Dialister* spp., where a high normalized burden may reflect either enrichment of the curated functional repertoire or concentration of these functions within a compact proteome. Therefore, these elevated normalized values should be interpreted as relative functional density rather than direct evidence of increased pathway activity or metabolic output. Multi-omics approaches integrating metagenomics, metatranscriptomics, and metabolomics are needed to determine whether predicted functions are active and biologically relevant in vivo [35]. Furthermore, strain-level gene copy-number variation and broader functional heterogeneity can significantly alter the metabolic capacity and potential output of individual bacterial strains. Consequently, a single representative genome may not capture the full functional diversity of a taxon, and genome-level predictions should not be directly extrapolated to community-level metabolic function without considering strain composition, relative abundance, gene dosage, and ecological interactions [36,37]. Finally, RefSeq accessions and assembly status may change over time as reference genome databases are periodically updated; thus, genome identifiers should be interpreted according to the database release and download date reported in the Methods.

Finally, the present study should be regarded as a prioritization and hypothesis-generating framework. The findings highlight candidate bacterial functions that may connect osteoporosis-associated gut microbiota to metabolic, immunogenic, and ecological mechanisms involved in bone remodeling. Three functional axes emerge as particularly promising for future validation: specialized metabolic/MGC capacity, particularly fermentation/energy and redox/nitrogen-related functions; SCFA-related metabolic potential; and structural immunogenic potential involving LPS/lipid A, peptidoglycan/cell-wall, and flagellar/TLR5-related features. These axes warrant investigation through fecal metagenomics, targeted metabolomics of SCFAs and other microbial metabolites, and experimental models assessing intestinal barrier integrity, immune activation, osteoclastogenesis, and osteoblast function. Validation of these predictions using fecal metagenomics, targeted metabolomics of SCFAs, indoles, menaquinones, bile acids, and polyamines, as well as experimental models, will be essential to determine whether the identified functional patterns contribute causally to bone loss or represent adaptive signatures of an altered gut ecosystem in osteoporosis.

## 5. Conclusions

In conclusion, this in silico analysis reveals that osteoporosis-associated and comparator gut bacteria possess heterogeneous yet biologically plausible functional repertoires relevant to the gut–bone axis. The primary functional signatures include vitamin/cofactor metabolism, SCFA-related functions, peptidoglycan/cell-wall biosynthesis, carbohydrate-active enzyme repertoires, selected KEGG metabolic pathways, and metabolic gene clusters predicted by gutSMASH. Notably, integrating primary candidate hits with a stringent high-confidence subset and multidimensional scoring reveals that the predicted functional capacity of these bacteria cannot be inferred solely by taxonomic association. Instead, it depends on the interplay among metabolic potential, carbohydrate-processing capacity, specialized primary metabolism, and structural immunogenic features. These findings provide a prioritized framework for future metagenomic, metabolomic, and experimental studies to validate microbial functions that may influence intestinal barrier integrity, immune signaling, osteoclast activity, osteoblast function, and bone remodeling in osteoporosis.

## Figures and Tables

**Table 1:** Candidate gut bacterial taxa selected for in silico functional profiling in the context of osteoporosis and low bone mineral density.

Candidate Taxon	Proposed Representative Species for Genome Mining	Association Category	Evidence from Human Studies	Functional Rationale for Inclusion	Proposed Role in the Analysis	Reference
*Bacteroides* spp.	*Bacteroides fragilis*/*Bacteroides uniformis*	OP/low BMD-associated	Increased abundance in low-BMD or osteoporosis groups; negative association with BMD and positive association with β-CTX in postmenopausal women.	Bile acid metabolism, carbohydrate degradation, amino acid metabolism, and LPS-related inflammatory potential.	Main OP/low-BMD candidate	Becerra-Cervera et al., 2025; Wang et al., 2025 [18,20]
*Parabacteroides* spp.	*Parabacteroides distasonis*	OP/low BMD-associated	Enriched in postmenopausal women with low BMD and reported in osteopenia/osteoporosis cohorts.	Bile acid metabolism, succinate production, carbohydrate utilization, and host metabolic modulation.	Main OP/low-BMD candidate	Becerra-Cervera et al., 2025 [18]
*Barnesiella* spp.	*Barnesiella intestinihominis*	OP/low BMD-associated	Increased abundance in women with low BMD and implicated in osteoporosis-associated microbial shifts.	Carbohydrate metabolism, ecological remodeling, and interaction with inflammatory pathways.	Main OP/low-BMD candidate	Becerra-Cervera et al., 2025 [18]
*Odoribacter* spp.	*Odoribacter splanchnicus*	OP/low BMD-associated	Enriched in low-BMD groups and negatively associated with BMD.	SCFA-related metabolism, immune modulation, and potential involvement in osteoclastogenic signaling.	Main OP/low-BMD candidate	Becerra-Cervera et al., 2025 [18]
*Sutterella* spp.	*Sutterella wadsworthensis*	OP/low BMD-associated	Increased abundance in women with low BMD.	Inflammatory potential, LPS-associated signaling, mucosal immune interaction, and IgA degradation.	Main OP/low-BMD candidate	Becerra-Cervera et al., 2025 [18]
*Butyricimonas* spp.	*Butyricimonas virosa*	OP/low BMD-associated	Enriched in low-BMD groups and negatively associated with BMD/T-score.	Butyrate-related metabolism and carbohydrate fermentation.	Main OP/low-BMD candidate	Becerra-Cervera et al., 2025 [18]
*Coprobacter* spp.	*Coprobacter fastidiosus*	OP/low BMD-associated	Increased abundance in women with low BMD and negatively correlated with bone parameters.	Anaerobic carbohydrate metabolism and contribution to altered microbial metabolic networks.	Main OP/low-BMD candidate	Becerra-Cervera et al., 2025 [18]
*Oscillibacter* spp.	*Oscillibacter valericigenes*	OP/low BMD-associated	Enriched in low-BMD groups and reported in osteoporosis-related cohorts.	Valerate production, SCFA metabolism, and immune-metabolic interactions.	Main OP/low-BMD candidate	Becerra-Cervera et al., 2025 [18]
*Dialister* spp.	*Dialister invisus*/*Dialister succinatiphilus*	OP-associated	Enriched in primary osteoporosis and proposed as a discriminatory microbial biomarker.	Potential links with IL-6-mediated inflammation and bone loss.	Main OP/low-BMD candidate	Xu et al., 2020 [21]
*Eggerthella* spp.	*Eggerthella lenta*	OP-associated	Increased abundance in older adults with osteoporosis compared with controls.	Steroid, bile acid, and xenobiotic metabolism; possible interaction with vitamin D pathways.	Main OP/low-BMD candidate	Das et al., 2019 [19]
*Actinomyces* spp.	*Actinomyces naeslundii*/*Actinomyces odontolyticus*	OP-associated	More abundant in osteoporosis among older adults.	Potential association with inflammation and oral–gut microbial translocation.	Secondary OP candidate	Das et al., 2019 [19]
*Turicibacter* spp.	*Turicibacter sanguinis*	OP-associated	Reported as enriched in postmenopausal osteoporosis.	Interaction with lipid metabolism, immune signaling, and inflammatory status.	Secondary OP candidate	Wang et al., 2025 [20]
*Catenibacterium* spp.	*Catenibacterium mitsuokai*	OP/marker-associated	Differentially associated with osteoporosis and bone turnover markers.	Fermentation-related metabolism and potential association with osteocalcin profiles.	Secondary OP candidate	Wang et al., 2025 [20]
*Agathobacter* spp.	*Agathobacter rectalis*	Normal BMD/potentially protective	Positively associated with BMD in postmenopausal women.	Butyrate production, CAZyme repertoire, and anti-inflammatory potential.	Normal-BMD-associated comparator	Becerra-Cervera et al., 2025 [18]
*Coprococcus* spp.	*Coprococcus comes*/*Coprococcus eutactus*	Normal BMD/potentially protective	Positively associated with BMD and T-score.	SCFA production and anti-inflammatory metabolic activity.	Normal-BMD-associated comparator	Becerra-Cervera et al., 2025 [18]
*Dorea* spp.	*Dorea longicatena*/*Dorea formicigenerans*	Normal BMD/potentially protective	Higher abundance associated with normal BMD.	Carbohydrate fermentation and maintenance of microbial metabolic stability.	Normal-BMD-associated comparator	Becerra-Cervera et al., 2025 [18]
*Ruminococcus torques* group	*Ruminococcus torques*	Normal BMD/potentially protective	Positively associated with BMD in postmenopausal women.	Mucin degradation, CAZyme diversity, and gut barrier-related functions.	Normal-BMD-associated comparator	Becerra-Cervera et al., 2025 [18]
*Megamonas* spp.	*Megamonas funiformis*/*Megamonas hypermegale*	Normal BMD/potentially protective	Positively associated with BMD and osteocalcin; enriched in healthy controls.	SCFA production and amino acid/carbohydrate metabolism.	Normal-BMD-associated comparator	Wang et al., 2025 [20]
*Roseburia intestinalis*	*Roseburia intestinalis*	Normal BMD/potentially protective	Positively associated with BMD in postmenopausal women.	Butyrate production, immune regulation, intestinal barrier maintenance, and osteoclast modulation.	Normal-BMD-associated comparator	Wang et al., 2025 [20]
*Faecalibacterium* spp.	*Faecalibacterium prausnitzii*	Context-dependent/contradictory	Enriched in primary osteoporosis in one study but generally considered a beneficial anti-inflammatory taxon.	Butyrate production, gut barrier support, and anti-inflammatory activity.	Functional comparator/contradictory candidate	Xu et al., 2020; Rettedal et al., 2021 [10,21]

BMD, bone mineral density; OP, osteoporosis; β-CTX, beta C-terminal telopeptide of type I collagen; SCFA, short-chain fatty acid; LPS, lipopolysaccharide; CAZyme, carbohydrate-active enzyme. When the original evidence was reported at the genus level, the representative species listed here should be interpreted as genome-mining candidates rather than species-specific clinical associations. Final inclusion in comparative genomic analyses will depend on genome availability, assembly quality, completeness, contamination metrics, and taxonomic consistency according to RefSeq, GenBank, and GTDB databases.

**Table 2:** Global distribution of curated gut–bone axis functional signatures across osteoporosis-associated bacterial genomes.

Functional Signature	Genomes Detected	Candidate Proteins	Unique KEGG Orthologs	Unique EC Numbers	Gut–Bone Axis Interpretation
Vitamin B12/cobalamin	26	690	98	51	Cofactor-related microbial metabolism; potential contribution to nutrient-linked host–microbe interactions.
Folate/one-carbon metabolism	26	553	69	47	One-carbon and nucleotide metabolism; potentially relevant to microbial growth and immunometabolic crosstalk.
Peptidoglycan/cell-wall biosynthesis	26	520	70	39	Cell-wall MAMP repertoire; plausible innate immune and osteoimmune signaling interface.
Butyrate-related metabolism	26	382	43	24	SCFA-related anti-inflammatory and epithelial-barrier signaling potential; key gut–bone axis candidate.
Propionate-related metabolism	26	215	34	23	SCFA-related metabolic and immune signaling potential; may complement acetate/butyrate cross-feeding.
Tryptophan/indole metabolism	26	133	25	19	Indole/AhR-linked signaling potential; relevant to barrier integrity and immune regulation.
Polyamine metabolism	25	118	24	16	Microbial polyamine turnover; potentially linked to epithelial renewal and inflammatory tone.
Acetate-related metabolism	25	86	16	8	SCFA production/cross-feeding potential; foundational microbial metabolic output.
Bile acid-related functions	23	60	9	7	Potential bile acid transformation functions; relevant to FXR/TGR5-linked metabolic and inflammatory pathways.
LPS/lipid A biosynthesis	21	267	56	23	Endotoxin-related MAMP potential; plausible TLR4-mediated pro-inflammatory/osteoclastogenic interface.
Flagellar machinery/TLR5-related	19	185	50	4	Flagellin/motility-associated immune signaling potential through TLR5-related pathways.
Vitamin K/menaquinone biosynthesis	19	128	30	18	Menaquinone biosynthesis potential; biologically relevant to bone biology, but host-level contribution requires validation.

Abbreviations: EC, Enzyme Commission number; KEGG, Kyoto Encyclopedia of Genes and Genomes. Values represent curated candidate annotations and should be interpreted as genomic potential rather than experimentally validated metabolite production.

## Data Availability

The bacterial reference genomes analyzed in this study are publicly available from the NCBI RefSeq database using the assembly accessions listed in Supplementary Table S1. The processed genome-level functional annotation summaries, curated gut–bone axis signature matrices, CAZyme profiles, KEGG pathway summaries, gutSMASH-predicted metabolic gene cluster outputs, and integrated scoring results are provided in the Supplementary Materials. The custom scripts used for downstream parsing, matrix construction, normalization, integrated scoring, sensitivity analysis, and visualization are available in Zenodo at https://doi.org/10.5281/zenodo.21264785 (accessed on 8 July 2026). No new human participant-level data were generated in this study.

## Supplementary Materials

The following supporting information can be downloaded at: https://www.mdpi.com/article/10.3390/cells15171573/s1, Figure S1: Genome-normalized CAZyme burden, class composition, diversity, and recurrent CAZy families across 26 osteoporosis-associated gut bacterial genomes; Figure S2: Normalized CAZyme class burden across 26 osteoporosis-associated gut bacterial genomes; Figure S3: Normalized KEGG pathway burden across 26 osteoporosis-associated gut bacterial genomes; Figure S4: gutSMASH-predicted metabolic gene cluster burden and product-category landscape across 26 osteoporosis-associated gut bacterial genomes; Figure S5: Integrated functional landscape across 26 gut bacterial genomes; Figure S6: Robustness of normalized gut–bone axis burden to strict high-confidence curation; Table S1: Representative bacterial genomes, RefSeq accessions, genome metadata, and assembly quality information; Table S2: Feature-level evidence supporting curated gut–bone axis functional signatures; Table S3: Curation-impact analysis comparing primary candidate hits with the strict high-confidence subset; Table S4: Genome-by-signature count and presence/absence matrices for curated gut–bone axis signatures; Table S5: Genome-wide annotation summary across the 26 bacterial genomes; Table S6: Normalized gut–bone axis burden, metabolic–immunogenic profile classification, and primary-versus-strict sensitivity analysis; Table S7: CAZyme repertoire summary by genome and enzyme class; Table S8: Selected KEGG pathway burden across the genome panel; Table S9: gutSMASH-predicted metabolic gene clusters/protoclusters across bacterial genomes; Table S10: Genome-level gutSMASH metabolic gene cluster summaries; Table S11: Sensitivity analysis of integrated functional rankings; Table S12: Integrated functional classification of osteoporosis-associated and comparator gut bacterial genomes.

## Abbreviations

The following abbreviations are used in this manuscript:

AA	Auxiliary activities
AhR	Aryl hydrocarbon receptor
antiSMASH	Antibiotics and Secondary Metabolite Analysis Shell
BMD	Bone mineral density
CAZy	Carbohydrate-Active enZYmes database
CAZyme	Carbohydrate-active enzyme
CBM	Carbohydrate-binding module
CE	Carbohydrate esterase
COG	Clusters of Orthologous Groups
dbCAN	Database for automated Carbohydrate-active enzyme Annotation
EC	Enzyme Commission
GH	Glycoside hydrolase
GT	Glycosyltransferase
IGF-1	Insulin-like growth factor 1
KEGG	Kyoto Encyclopedia of Genes and Genomes
LPS	Lipopolysaccharide
MGC	Metabolic gene cluster
NCBI	National Center for Biotechnology Information
OP	Osteoporosis
PFAM	Protein family database
PL	Polysaccharide lyase
RANKL	Receptor activator of nuclear factor kappa-B ligand
RefSeq	Reference Sequence Database
SCFA	Short-chain fatty acid
TLR5	Toll-like receptor 5
WNT10B	Wingless-type MMTV integration site family member 10B
