# Supplementary material for: Genome-Resolved Functional Profiling of Osteoporosis-Associated Gut Bacteria Highlights Putative Metabolic and Immunogenic Signatures of the Gut–Bone Axis
Source: Cells. 2026 Aug 29;15(17):1573. doi: 10.3390/cells15171573 (PMC13565464; doi:10.3390/cells15171573)
Supplement: Supplementary file 1 [file cells-15-01573-s001.zip › Supplementary_Figures.pdf]

Genome-resolved functional profiling of osteoporosis-associated gut bacteria highlights putative metabolic and immunogenic signatures of the gut–bone axis

Rogelio F. Jiménez-Ortega [1], Alejandra I. Ortega-Meléndez [2], Juan F. Montes-García [3], Erasmo Negrete-Abascal [3], Nelly Patiño[4], Eric Gustavo Ramírez-Salazar [5], Rosalba Sevilla-Montoya [6] Rafael Velázquez-Cruz [7], and Alberto Hidalgo-Bravo [1,] *

Alberto Hidalgo-Bravo, PhD
Servicio de Medicina Genómica
Instituto Nacional de Rehabilitación Luis Guillermo Ibarra Ibarra (INRLGII)
Mexico City 14389, Mexico
E-mail: dr_genetica@yahoo.com
Tel.: +52 55 5999 1000, Ext. 10030 y 10031

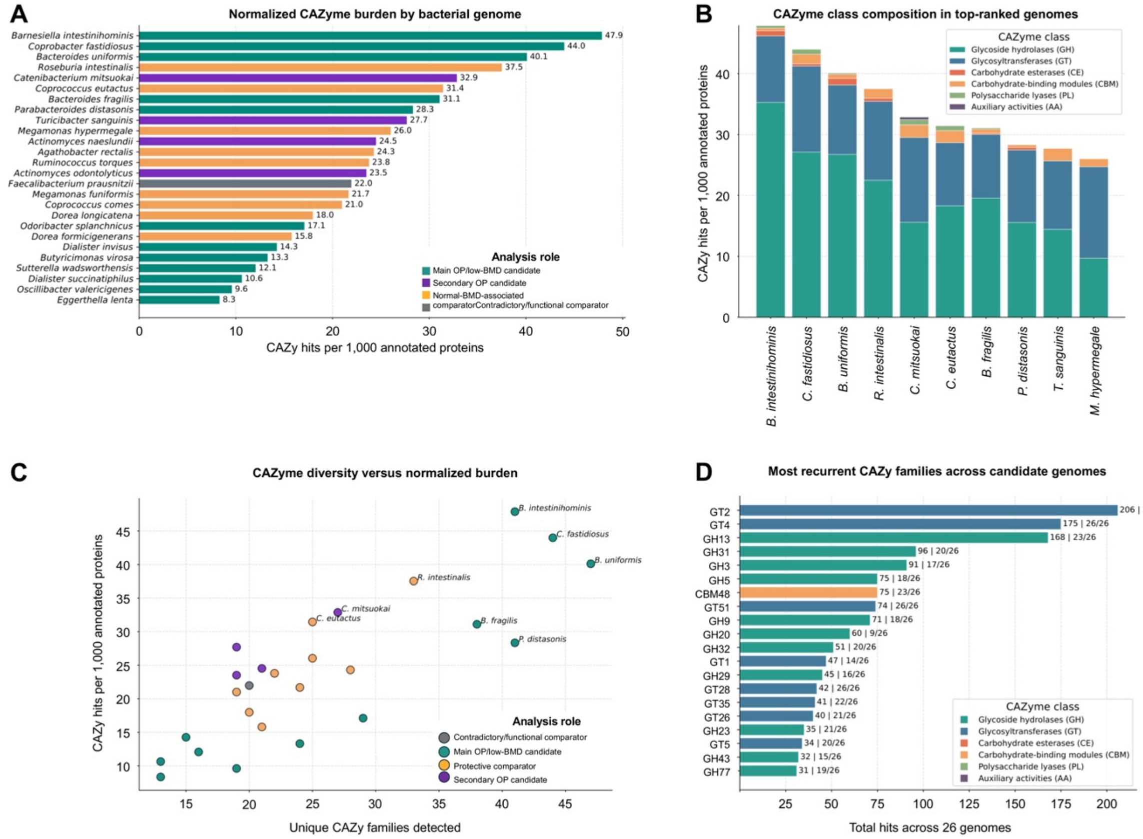

Figure S1. CAZyme repertoire and normalized carbohydrate-active enzyme burden across the 26 bacterial genomes. (A) Genome-level CAZyme burden expressed as CAZy hits per 1,000 annotated proteins and ranked from highest to lowest. Bar colors indicate the analytical role assigned to each genome. (B) CAZyme class composition among the ten genomes with the highest normalized burden, showing the relative contribution of glycoside hydrolases (GH), glycosyltransferases (GT), carbohydrate esterases (CE), carbohydrate-binding modules (CBM), polysaccharide lyases (PL), and auxiliary activities (AA). (C) Relationship between the number of unique CAZy families detected and normalized CAZyme burden, illustrating differences between CAZyme repertoire diversity and functional density across genomes. Selected high-burden genomes are

labeled. (D) Most recurrent CAZy families across the 26 genomes, ranked by total number of detected hits; labels indicate the total hit count and the number of genomes in which each family was detected.

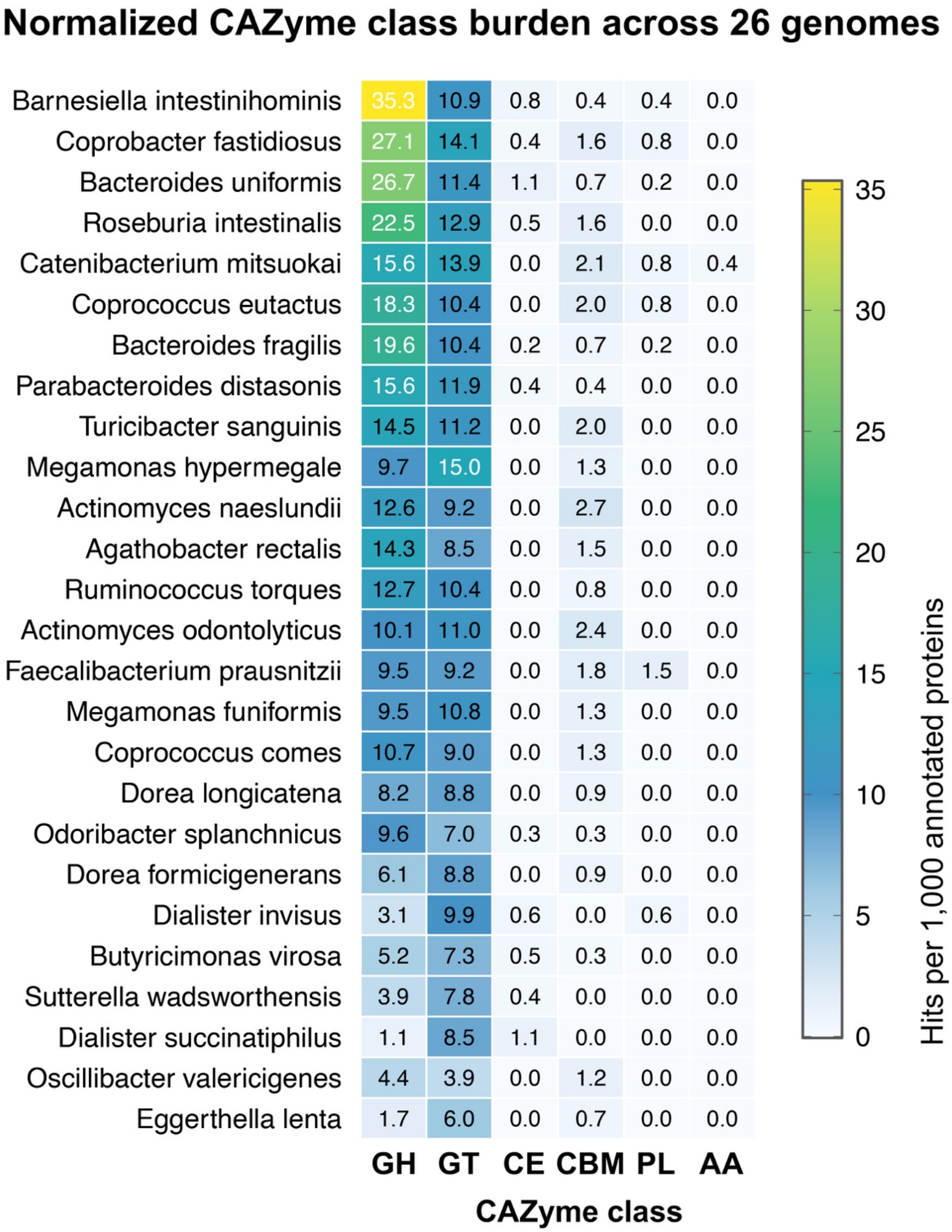

Figure S2. Normalized CAZyme class burden across 26 bacterial genomes. Heatmap showing the genome-normalized distribution of the six major carbohydrate-active enzyme classes across the bacterial panel. Values represent CAZyme hits per 1,000 annotated proteins. Rows correspond to individual bacterial genomes and

columns to glycoside hydrolases (GH), glycosyltransferases (GT), carbohydrate esterases (CE), carbohydrate-binding modules (CBM), polysaccharide lyases (PL), and auxiliary activities (AA). Higher color intensity indicates greater normalized CAZyme burden. GH and GT represented the predominant CAZyme classes, although their relative contribution varied substantially among genomes.

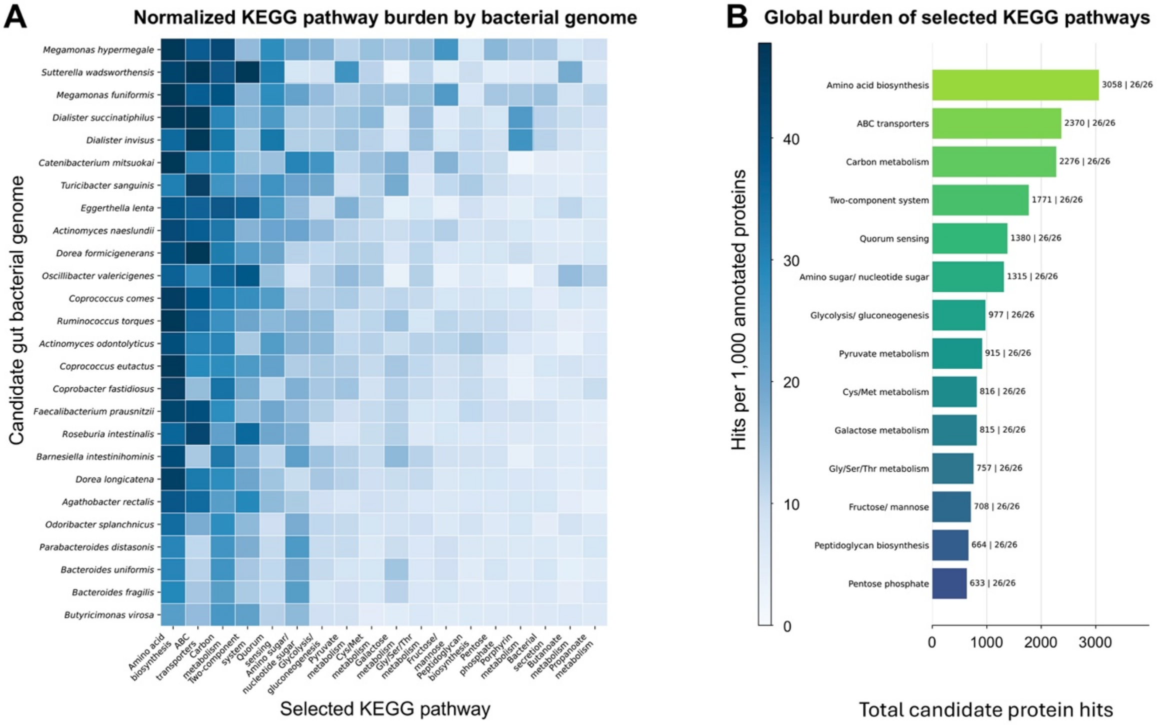

Figure S3. Normalized KEGG pathway burden across 26 bacterial genomes. (A) Heatmap showing the normalized burden of selected gut–bone axis-relevant KEGG pathways across the 26 representative bacterial genomes, expressed as hits per 1,000 annotated proteins. Rows represent bacterial genomes and columns represent selected metabolic, transport, signaling, carbohydrate-processing, and cell-envelope-associated pathways. Greater color intensity indicates higher normalized pathway burden. (B) Global burden of selected KEGG pathways across the complete genome panel, ranked by total candidate protein hits. Labels indicate the total number of hits and the number of genomes in which each pathway was detected. Amino acid biosynthesis, ABC transporters, carbon metabolism, two-component systems, and quorum sensing were among the most broadly represented functional categories.

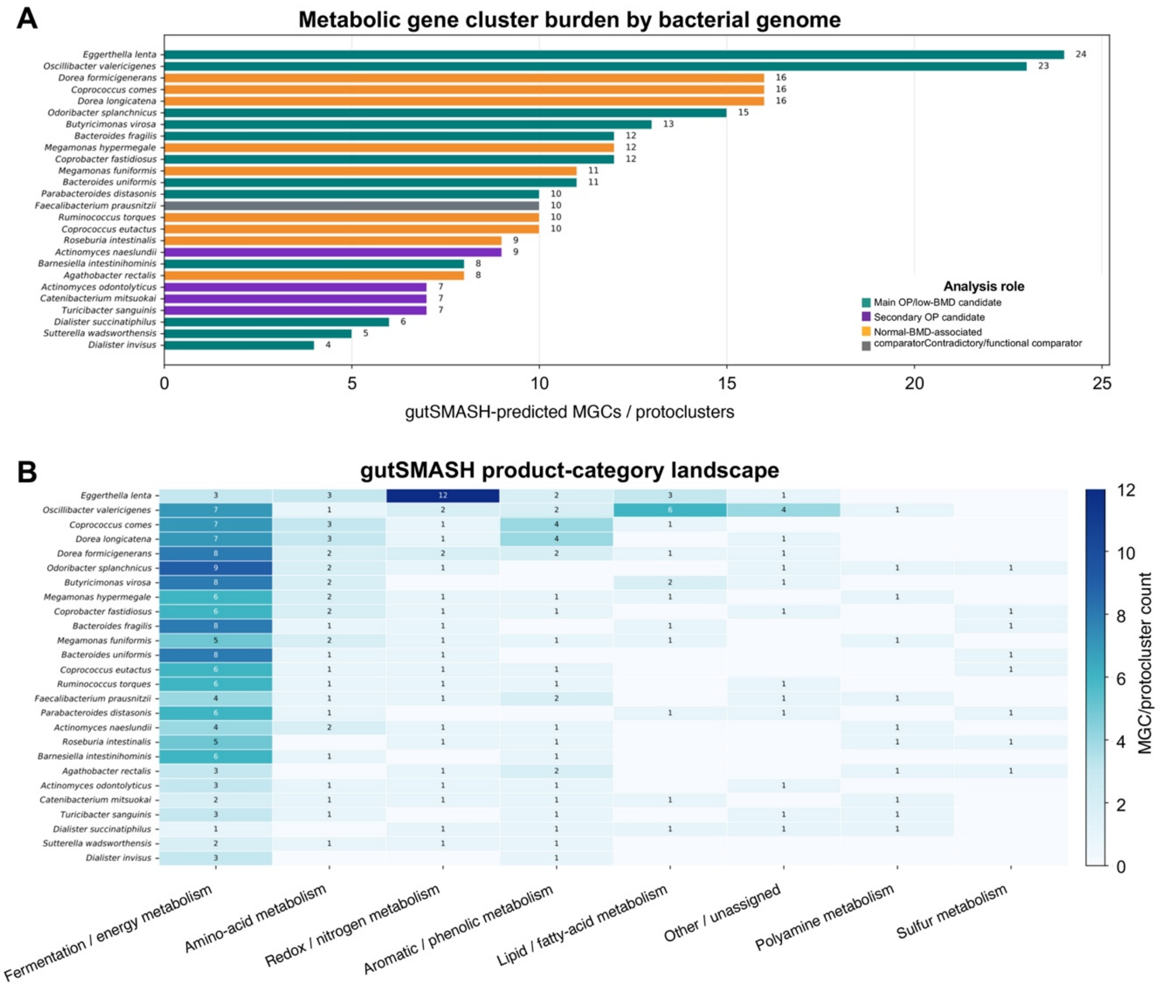

Figure S4. gutSMASH-predicted metabolic gene cluster burden and product-category landscape across 26 bacterial genomes. (A) Total number of gutSMASH-predicted metabolic gene clusters (MGCs)/protoclusters detected in each genome, ranked from highest to lowest and colored according to analytical role. *Eggerthella lenta* and *Oscillibacter valericigenes* displayed the greatest overall MGC burden, followed by several genomes with intermediate cluster counts. (B) Heatmap showing the distribution of predicted MGCs across eight functional product categories: fermentation/energy metabolism, amino-acid metabolism, redox/nitrogen metabolism, aromatic/phenolic metabolism, lipid/fatty-acid metabolism, other/unassigned functions, polyamine metabolism, and sulfur metabolism. Cell values represent the number of MGCs/protoclusters assigned to each category within each genome.

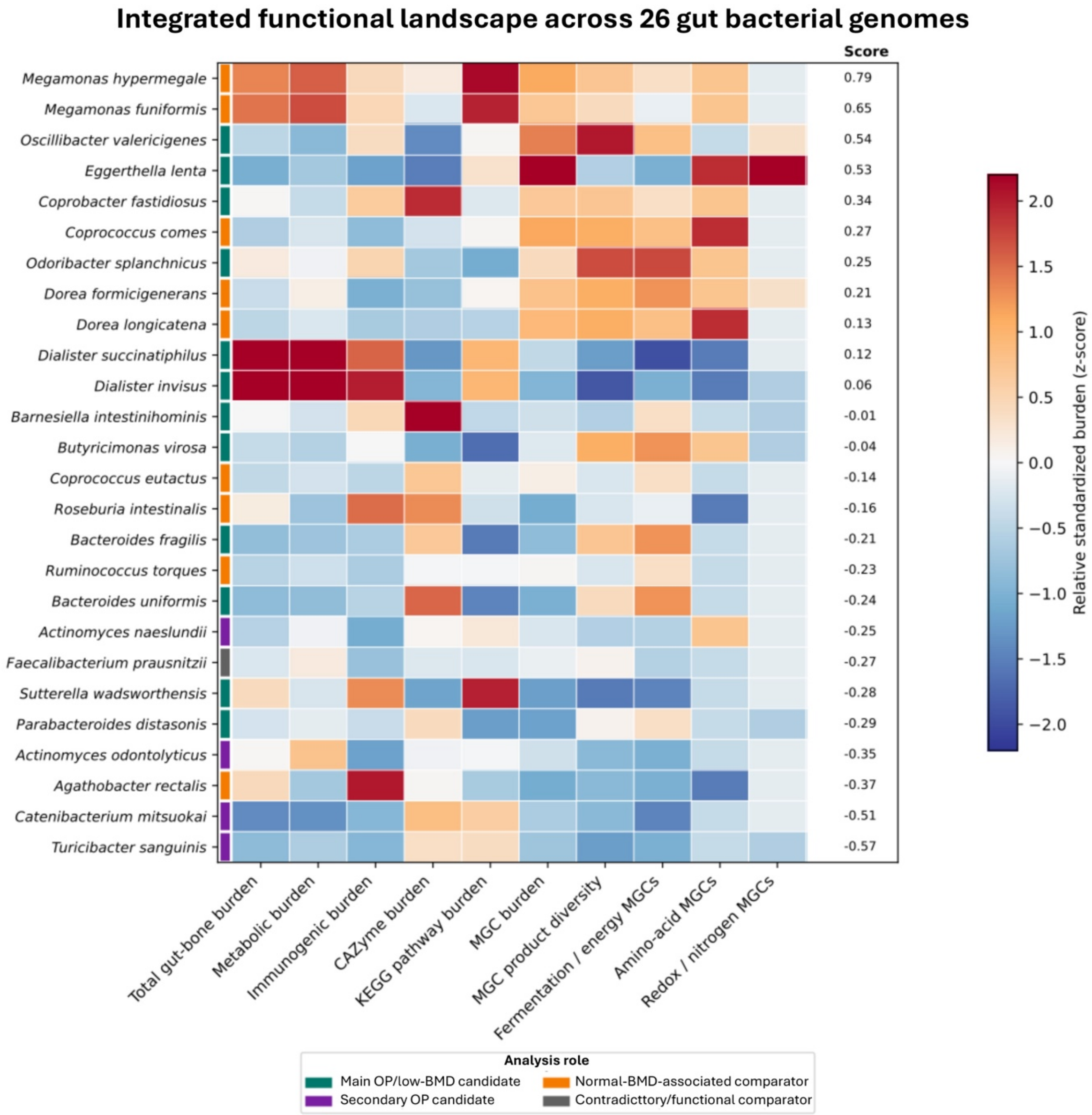

Figure S5. Integrated functional landscape across 26 gut bacterial genomes. Heatmap summarizing standardized genome-level functional burdens across ten dimensions: total gut–bone axis burden, metabolic burden, immunogenic burden, CAZyme burden, selected KEGG pathway burden, MGC burden, MGC product diversity, fermentation/energy MGCs, amino-acid MGCs, and redox/nitrogen MGCs. Values are displayed as relative standardized burdens (z-scores), with warmer colors indicating higher and cooler colors indicating lower values relative to the analyzed genome panel. Genomes are ordered according to the global integrated functional score shown at the right, while the colored strip at the left indicates analytical role. The integrated score represents comparative multidimensional functional potential and should not be interpreted as a biological effect-size or as evidence of a beneficial or adverse effect.

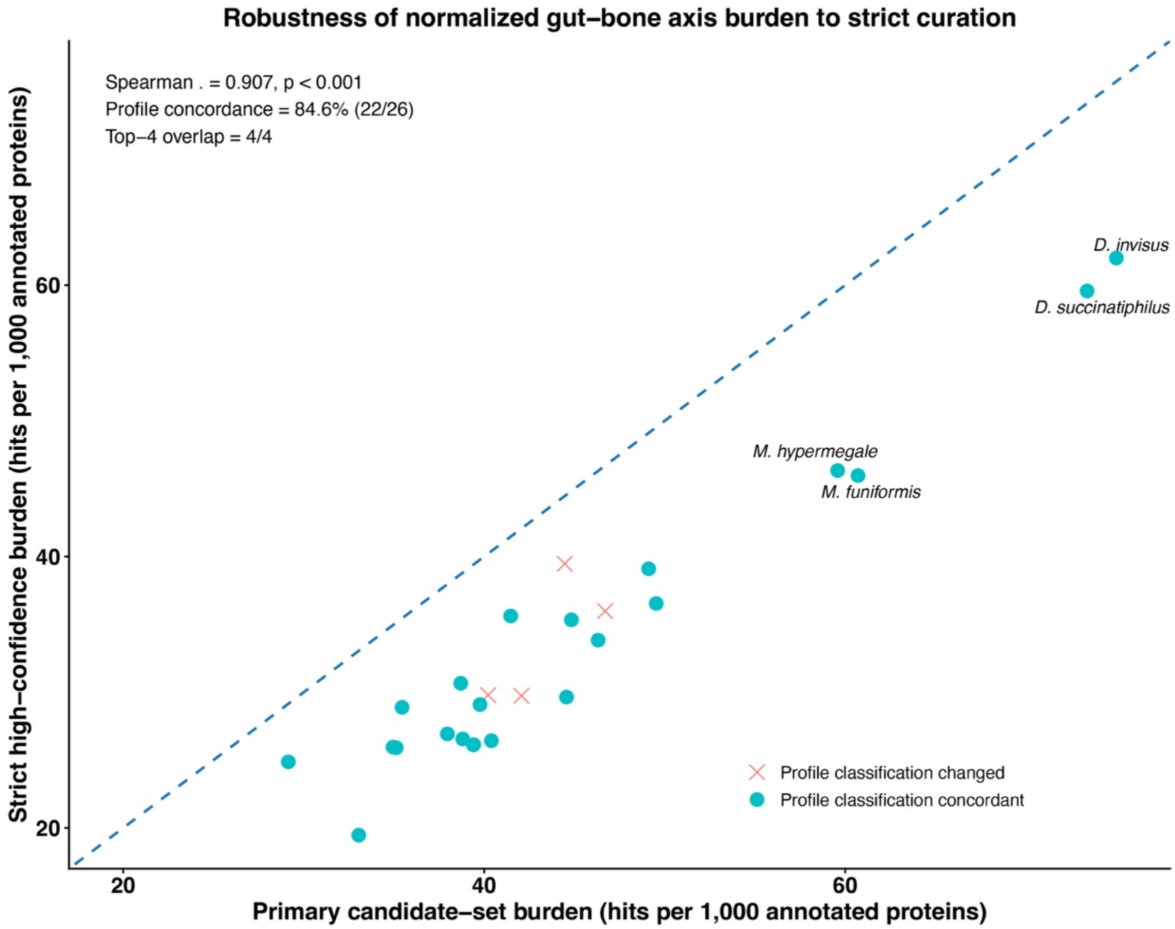

Figure S6. Robustness of normalized gut–bone axis burden to strict high-confidence curation. Scatter plot comparing genome-level normalized gut–bone axis burden calculated from the primary candidate set with that obtained using exclusively the strict high-confidence subset across the 26 bacterial genomes. Burdens are expressed as hits per 1,000 annotated proteins. The dashed diagonal represents the identity line. Filled circles indicate genomes retaining the same metabolic–immunogenic profile classification under strict curation, whereas crosses indicate genomes whose classification changed. The primary and strict analyses showed strong rank concordance (Spearman $\rho = 0.907$, $p < 0.001$), with 22 of 26 genomes (84.6%) retaining the same profile classification and complete overlap among the four highest-ranked genomes. *Dialister invisus* and *Dialister succinatiphilus* remained among the highest-burden genomes, while *Megamonas hypermegale* and *Megamonas funiformis* remained highly prioritized under the strict analysis.
