# Supplementary material for: Genome-Resolved Functional Profiling of Osteoporosis-Associated Gut Bacteria Highlights Putative Metabolic and Immunogenic Signatures of the Gut–Bone Axis
Source: Cells. 2026 Aug 29;15(17):1573. doi: 10.3390/cells15171573 (PMC13565464; doi:10.3390/cells15171573)
Supplement: Supplementary file 1 [file cells-15-01573-s001.zip › Supplementary_Tables/Table S1.pdf]

Table S1. Genome retrieval and quality curation of the 26 representative gut bacterial species selected for in silico functional profiling

No.	Candidate species	Analysis role	Selected organism	Assembly accession	Source	Assembly level	Genome size (Mb)	Contigs	N50 (Mb)	GC (%)	Type material / note	Inclusion
1	*Bacteroides fragilis*	OP / low BMD candidate	*Bacteroides fragilis*	GCF_040687795.1	Refseq	Complete Genome	5.73	1	5.73	43.5	Not specified	Included
2	*Bacteroides uniformis*	OP / low BMD candidate	*Bacteroides uniformis*	GCF_018291785.1	Refseq	Complete Genome	5.47	3	5.36	46.5	Not specified	Included
3	*Parabacteroides distasonis*	OP / low BMD candidate	*Parabacteroides distasonis*	GCF_024758965.1	Refseq	Complete Genome	5.53	1	5.53	45.5	Not specified	Included
4	*Barnesiella intestinihominis*	OP / low BMD candidate	*Barnesiella intestinihominis*	GCF_046603765.1	Refseq	Complete Genome	3.26	1	3.26	43.5	Not specified	Included
5	*Odoribacter splanchnicus*	OP / low BMD candidate	*Odoribacter splanchnicus* DSM 20712	GCF_000190535.1	Refseq	Complete Genome	4.39	1	4.39	43.5	assembly from type material	Included
6	*Sutterella wadsworthensis*	OP / low BMD candidate	*Sutterella wadsworthensis*	GCF_016724805.1	Refseq	Complete Genome	3.03	1	3.03	55.0	assembly from type material	Included
7	*Butyricimonas virosa*	OP / low BMD candidate	*Butyricimonas virosa*	GCF_025148635.1	Refseq	Complete Genome	4.81	1	4.81	42.5	assembly from type material	Included

8	*Coprobacter fastidiosus*	OP / low BMD candidate	*Coprobacter fastidiosus*	GCF_030296935.1	Refseq	Complete Genome	3.44	1	3.44	38.5	assembly from type material	Included
9	*Oscillibacter valericigenes*	OP / low BMD candidate	*Oscillibacter valericigenes* Sjm18-20	GCF_000283575.1	Refseq	Complete Genome	4.47	2	4.41	53.0	Not specified	Included
10	*Dialister invisus*	OP-associated candidate	*Dialister invisus* DSM 15470	GCF_000160055.1	Refseq	Scaffold	1.90	2	1.90	45.5	assembly from type material	Included
11	*Dialister succinatiphilus*	OP-associated candidate	*Dialister succinatiphilus*	GCF_928721695.1	Refseq	Complete Genome	2.49	1	2.49	51.0	Not specified	Included
12	*Eggerthella lenta*	OP-associated candidate	*Eggerthella lenta*	GCF_021378705.1	Refseq	Complete Genome	3.80	1	3.80	64.0	Not specified	Included
13	*Actinomyces naeslundii*	Secondary OP candidate	*Actinomyces naeslundii*	GCF_023170065.1	Refseq	Complete Genome	3.20	1	3.20	67.5	Not specified	Included
14	*Actinomyces odontolyticus*	Secondary OP candidate	*Schaalia odontolytica*	GCF_055396835.1	Refseq	Complete Genome	2.68	1	2.68	65.5	Not specified	Included
15	*Turicibacter sanguinis*	Secondary OP candidate	*Turicibacter sanguinis*	GCF_013046825.1	Refseq	Complete Genome	3.00	1	3.00	34.5	assembly from type material	Included

16	*Catenibacterium mitsuokai*	Secondary OP candidate	*Catenibacterium mitsuokai*	GCF_025148785.1	Refseq	Complete Genome	2.65	1	2.65	34.5	assembly from type material	Included
17	*Agathobacter rectalis*	Normal-BMD-associated comparator	*Agathobacter rectalis*	GCF_036419255.1	Refseq	Complete Genome	4.01	2	3.76	40.0	Not specified	Included
18	*Coprococcus comes*	Normal-BMD-associated comparator	*Allocoprococcus comes*	GCF_016904155.1	Refseq	Complete Genome	3.37	1	3.37	42.5	Not specified	Included
19	*Coprococcus eutactus*	Normal-BMD-associated comparator	*Coprococcus eutactus* ATCC 27759	GCF_020735705.1	Refseq	Complete Genome	3.11	1	3.11	43.0	Not specified	Included
20	*Dorea longicatena*	Normal-BMD-associated comparator	*Dorea longicatena*	GCF_023008345.1	Refseq	Complete Genome	3.44	1	3.44	41.0	Not specified	Included
21	*Dorea formicigenerans*	Normal-BMD-associated comparator	*Dorea formicigenerans*	GCF_051626395.1	Refseq	Complete Genome	3.59	1	3.59	40.5	Not specified	Included
22	*Ruminococcus torques*	Normal-BMD-	*Mediterraneibacter torques*	GCF_044591705.1	Refseq	Complete Genome	2.98	1	2.98	42.0	Not specified	Included

		associated comparator										
23	*Megamonas funiformis*	Normal-BMD-associated comparator	*Megamonas funiformis*	GCF_010669225.1	Refseq	Complete Genome	2.57	2	2.52	31.5	assembly from type material	Included
24	*Megamonas hypermegale*	Normal-BMD-associated comparator	*Megamonas hypermegale*	GCF_963931125.1	Refseq	Complete Genome	2.62	1	2.62	42.5	Not specified	Included
25	*Roseburia intestinalis*	Normal-BMD-associated comparator	*Roseburia intestinalis* L1-82	GCF_900537995.1	Refseq	Complete Genome	4.49	1	4.49	42.5	assembly from type material	Included
26	*Faecalibacterium prausnitzii*	Context-dependent comparator	*Faecalibacterium prausnitzii*	GCF_049532955.1	Refseq	Complete Genome	3.14	1	3.14	57.0	Not specified	Included

Abbreviations and notes: OP, osteoporosis; BMD, bone mineral density; N50, scaffold N50; GC, guanine-cytosine content. Genome size and N50 are shown in megabases. Type-material information was coded as “Not specified” when absent in NCBI metadata. Current NCBI taxonomy is preserved in the selected organism column; therefore, some candidates are represented by updated names, including Actinomyces odontolyticus as Schaalia odontolytica, Coprococcus comes as Allocoprococcus comes, and Ruminococcus torques as Mediterraneibacter torques. All listed accessions were retained for downstream functional annotation and genome mining.
