# Supplementary material for: Genome-Resolved Functional Profiling of Osteoporosis-Associated Gut Bacteria Highlights Putative Metabolic and Immunogenic Signatures of the Gut–Bone Axis
Source: Cells. 2026 Aug 29;15(17):1573. doi: 10.3390/cells15171573 (PMC13565464; doi:10.3390/cells15171573)
Supplement: Supplementary file 1 [file cells-15-01573-s001.zip › Supplementary_Tables/Table S10.pdf]

Table S10 A. Genome-level summary of gutSMASH-predicted metabolic gene cluster burden.

Bacterium	Analysis role	Genome accession	proteins_annotated	MGC_count	Unique_MGC_products	MGCs_per_1000_proteins	Mean_CDS_per_MGC	Top gutSMASH products	Top product categories
Eggerthella lenta	Main OP/low-BMD candidate	GCF_021378705.1	2996	24	8	8.01068090787717	11.541666666666666	Molybdopterin_dependent_oxidoreductase (12); Flavoenzyme_lipids_catabolism (3); Arginine2_Hcarbonate (2); Others_HGD_unassigned (2); Phenylpyruvate_ferredoxin_oxidoreductase (2)	Redox / nitrogen metabolism (12); Amino-acid metabolism (3); Fermentation / energy metabolism (3)
Oscillibacter valericigenes	Main OP/low-BMD candidate	GCF_000283575.1	4058	23	16	5.667816658452439	8.91304347826087	OD_fatty_acids (4); PFOR_II_pathway (4); xanthine_dehydrogenase (2); Arginine2putrescine (1); Flavoenzyme_lipids_catabolism (1)	Fermentation / energy metabolism (7); Lipid / fatty-acid metabolism (6); Other / unassigned (4)

Coprococcus comes	Normal-BMD-associated comparator	GCF_016904155.1	3000	16	13	5.333333333333333	7.875	Others_HGD_unassigned (4); Fumarate2succinate (1); GR_fatty_acids (1); Glycine_cleavage (1); Glycine_reductase (1)	Fermentation / energy metabolism (7); Aromatic / phenolic metabolism (4); Amino-acid metabolism (3)
Dorea longicatena	Normal-BMD-associated comparator	GCF_023008345.1	3169	16	13	5.0489113284947935	8.625	Others_HGD_unassigned (3); Phenylpyruvate_ferredoxin_oxidoreductase (2); Flavoenzyme_AA_peptides_catabolism (1); Glycine_cleavage (1); Glycine_reductase (1)	Fermentation / energy metabolism (7); Aromatic / phenolic metabolism (4); Amino-acid metabolism (3)
Dorea formicigenerans	Normal-BMD-associated comparator	GCF_051626395.1	3292	16	13	4.860267314702308	10.4375	Others_HGD_unassigned (2); Phenylpyruvate_ferredoxin_oxidoreductase (2); xanthine_dehydrogenase (2);	Fermentation / energy metabolism (8); Amino-acid metabolism (2); Aromatic / phenolic metabolism (2)

Odoribacter splanchnicus	Main OP/low-BMD candidate	GCF_000190535.1	3448	15	15	4.3503480278422275	8.266666666666667	Fumarate2succinate (1); Glycine_cleavage (1) Fumarate2succinate (1); Lysine_degradation (1); NADH_dehydrogenase_I (1); PFOR_II_pathway (1); Phenylpyruvate_ferredoxin_oxidoreductase (1)	Fermentation / energy metabolism (9); Amino-acid metabolism (2); Other / unassigned (1)
Butyricimonas virosa	Main OP/low-BMD candidate	GCF_025148635.1	3681	13	13	3.5316490084216245	7.384615384615385	Arginine2putrescine (1); Fumarate2succinate (1); Lysine_degradation (1); NADH_dehydrogenase_I (1); OD_fatty_acids (1)	Fermentation / energy metabolism (8); Amino-acid metabolism (2); Lipid / fatty-acid metabolism (2)

Megamonas hypermegale	Normal-BMD-associated comparator	GCF_963931125.1	2266	12	12	5.29567519858782	8.416666666666666	Arginine2putrescine (1); Fumarate2succinate (1); Glycine_cleavage (1); Others_HGD_unassigned (1); PFOR_II_pathway (1)	Fermentation / energy metabolism (6); Amino-acid metabolism (2); Aromatic / phenolic metabolism (1)
Coprobacter fastidiosus	Main OP/low-BMD candidate	GCF_030296935.1	2546	12	12	4.713275726630008	6.0	Fumarate2succinate (1); OD_AA_metabolism (1); OD_lactate_related (1); Others_HGD_unassigned (1); PFOR_II_pathway (1)	Fermentation / energy metabolism (6); Amino-acid metabolism (2); Aromatic / phenolic metabolism (1)
Bacteroides fragilis	Main OP/low-BMD candidate	GCF_040687795.1	4599	12	12	2.609262883235486	6.916666666666667	Fumarate2succinate (1); NADH_dehydrogenase_I (1); PFOR_II_pathway (1); Phenylpyruvate_ferred	Fermentation / energy metabolism (8); Amino-acid metabolism (1); Lipid / fatty-acid metabolism (1)

								oxin_oxidoreductase (1); Pyruvate2acetate-formate (1)	
Megamonas funiformis	Normal-BMD-associated comparator	GCF_010669225.1	2306	11	11	4.770164787510842	9.545454545454545	Arginine2putrescine (1); Fumarate2succinate (1); GR_fatty_acids (1); Glycine_cleavage (1); Others_HGD_unassigned (1)	Fermentation / energy metabolism (5); Amino-acid metabolism (2); Aromatic / phenolic metabolism (1)
Bacteroides uniformis	Main OP/low-BMD candidate	GCF_018291785.1	4562	11	11	2.411223147742218	6.454545454545454	Fumarate2succinate (1); NADH_dehydrogenase_I (1); PFOR_II_pathway (1); Phenylpyruvate_ferredoxin_oxidoreductase (1); Pyruvate2acetate-formate (1)	Fermentation / energy metabolism (8); Amino-acid metabolism (1); Redox / nitrogen metabolism (1)
Coprococcus eutactus	Normal-BMD-associated comparator	GCF_020735705.1	2512	10	9	3.9808917197452227	7.6	Phenylpyruvate_ferredoxin_oxidoreductase (2);	Fermentation / energy metabolism (6); Amino-acid

								Adenylylsulfate_reductase (1); OD_AA_metabolism (1); Others_HGD_unassigned (1); Pyruvate2acetate-formate (1)	metabolism (1); Aromatic / phenolic metabolism (1)
Ruminococcus torques	Normal-BMD-associated comparator	GCF_044591705.1	2606	10	9	3.837298541826554	8.1	Rnf_complex (2); Flavoenzyme_sugar_catabolism (1); Others_HGD_unassigned (1); PFOR_II_pathway (1); Phenylpyruvate_ferredoxin_oxidoreductase (1)	Fermentation / energy metabolism (6); Amino-acid metabolism (1); Aromatic / phenolic metabolism (1)
Faecalibacterium prausnitzii	Contradictory/functional comparator	GCF_049532955.1	2731	10	10	3.6616623947272062	8.0	Bilirubin_reductase (1); Fumarate2succinate (1); OD_AA_metabolism (1);	Fermentation / energy metabolism (4); Aromatic / phenolic metabolism (2); Amino-acid metabolism (1)

Parabacteroides distasonis	Main OP/low-BMD candidate	GCF_024758965.1	4623	10	10	2.163097555699762	6.2	Others_HGD_unassigned (1); Putrescine2spermidine (1) Fumarate2succinate (1); OD_unknown (1); Phenylpyruvate_ferredoxin_oxidoreductase (1); Pyruvate2acetate-formate (1); Rnf_complex (1)	Fermentation / energy metabolism (6); Amino-acid metabolism (1); Lipid / fatty-acid metabolism (1)
Actinomyces naeslundii	Secondary OP candidate	GCF_023170065.1	2610	9	8	3.4482758620689653	9.555555555555555	Fumarate2succinate (2); Arginine2_Hcarbonate (1); NADH_dehydrogenase_I (1); Nitrate_reductase (1); Others_HGD_unassigned (1)	Fermentation / energy metabolism (4); Amino-acid metabolism (2); Aromatic / phenolic metabolism (1)
Roseburia intestinalis	Normal-BMD-associated comparator	GCF_900537995.1	3863	9	9	2.3297954957287086	7.888888888888889	Adenylylsulfate_reductase (1); Others_HGD_unassig	Fermentation / energy metabolism (5); Aromatic /

								ned (1); Putrescine2spermidine (1); Pyruvate2acetate-formate (1); Rnf_complex (1)	phenolic metabolism (1); Polyamine metabolism (1)
Barnesiella intestinihominis	Main OP/low-BMD candidate	GCF_046603765.1	2381	8	8	3.359932801343973	7.875	Fumarate2succinate (1); Glycine_cleavage (1); Others_HGD_unassigned (1); PFOR_II_pathway (1); Rnf_complex (1)	Fermentation / energy metabolism (6); Amino-acid metabolism (1); Aromatic / phenolic metabolism (1)
Agathobacter rectalis	Normal-BMD-associated comparator	GCF_036419255.1	3416	8	7	2.34192037470726	9.75	Others_HGD_unassigned (2); Adenylylsulfate_reductase (1); Putrescine2spermidine (1); Rnf_complex (1); acetate2butyrate (1)	Fermentation / energy metabolism (3); Aromatic / phenolic metabolism (2); Polyamine metabolism (1)
Actinomyces odontolyticus	Secondary OP candidate	GCF_055396835.1	2084	7	7	3.3589251439539347	8.857142857142858	Fumarate2succinate (1); NADH_dehydrogenase_I (1);	Fermentation / energy metabolism (3); Amino-acid metabolism (1);

							Nitrate_reductase (1); Others_HGD_unassigned (1); Pyruvate2acetate-formate (1)	Aromatic / phenolic metabolism (1)
Catenibacterium mitsuokai	Secondary OP candidate	GCF_025148785.1	2373	7	7	2.949852507374631	9.428571428571429	GR_fatty_acids (1); Others_HGD_unassigned (1); Putrescine2spermidine (1); Pyruvate2acetate-formate (1); Rnf_complex (1)	Fermentation / energy metabolism (2); Amino-acid metabolism (1); Aromatic / phenolic metabolism (1)
Turicibacter sanguinis	Secondary OP candidate	GCF_013046825.1	2491	7	6	2.8101164191087915	6.571428571428571	Rnf_complex (2); Anaerobic_sulfite_reductase (1); Arginine2putrescine (1); Others_HGD_unassigned (1); Putrescine2spermidine (1)	Fermentation / energy metabolism (3); Amino-acid metabolism (1); Aromatic / phenolic metabolism (1)

Dialister succinatiphilus	Main OP/low-BMD candidate	GCF_928721695.1	1880	6	6	3.1914893617021276	6.0	Ech_complex (1); OD_fatty_acids (1); Others_HGD_unassigned (1); Putrescine2spermidine (1); putative_2-oxoglutarate_ferredoxin_oxidoreductase (1)	Aromatic / phenolic metabolism (1); Fermentation / energy metabolism (1); Lipid / fatty-acid metabolism (1)
Sutterella wadsworthensis	Main OP/low-BMD candidate	GCF_016724805.1	2320	5	5	2.155172413793103	8.0	Arginine2putrescine (1); Fumarate2succinate (1); Molybdopterin_dependent_oxidoreductase (1); Others_HGD_unassigned (1); Pyruvate2acetate-formate (1)	Fermentation / energy metabolism (2); Amino-acid metabolism (1); Aromatic / phenolic metabolism (1)
Dialister invisus	Main OP/low-BMD candidate	GCF_000160055.1	1613	4	4	2.4798512089274642	5.75	Fumarate2succinate (1); Others_HGD_unassigned (1);	Fermentation / energy metabolism (3); Aromatic /

	PFOR_II_pathway (1); succinate2propionate (1)	phenolic metabolism (1)

Abbreviations: MGC, metabolic gene cluster; CDS, coding sequence; OP, osteoporosis; BMD, bone mineral density; gutSMASH, gut-specific Specialized Metabolite Analysis Shell; PFOR, pyruvate:ferredoxin oxidoreductase; Rnf, Rhodobacter nitrogen fixation complex; HGD, 2-hydroxyglutarate dehydrogenase-related/unassigned category.

Table S10 B. Global summary of recurrent gutSMASH products across candidate genomes.

gutSMASH product	Product category	Total_MGCs	Genomes_detected	Genomes_detected_percent	Mean_CDS	Max_CDS
Others_HGD_unassigned	Aromatic / phenolic metabolism	29	21	80.76923076923077	5.9655172413793105	13
Rnf_complex	Fermentation / energy metabolism	21	19	73.07692307692307	10.380952380952381	14
succinate2propionate	Fermentation / energy metabolism	18	18	69.23076923076923	11.333333333333334	16
Fumarate2succinate	Fermentation / energy metabolism	17	16	61.53846153846154	5.705882352941177	11
PFOR_II_pathway	Fermentation / energy metabolism	17	14	53.84615384615385	5.294117647058823	12
Pyruvate2acetate-formate	Fermentation / energy metabolism	16	16	61.53846153846154	6.1875	9

TPP_AA_metabolism	Amino-acid metabolism	16	16	61.53846153846154	7.625	14
Phenylpyruvate_ferredoxin_oxidoreductase	Fermentation / energy metabolism	15	11	42.30769230769231	6.333333333333333	13
xanthine_dehydrogenase	Redox / nitrogen metabolism	14	12	46.15384615384615	7.357142857142857	12
Molybdopterin_dependent_oxidoreductase	Redox / nitrogen metabolism	13	2	7.6923076923076925	12.923076923076923	38
Putrescine2spermidine	Polyamine metabolism	11	11	42.30769230769231	8.636363636363637	14
porA	Fermentation / energy metabolism	11	11	42.30769230769231	7.545454545454546	13
acetate2butyrate	Fermentation / energy metabolism	7	7	26.923076923076923	11.142857142857142	17
Arginine2putrescine	Amino-acid metabolism	6	6	23.076923076923077	8.0	13
Glycine_cleavage	Fermentation / energy metabolism	6	6	23.076923076923077	9.166666666666666	11

NADH_dehydrogenase_I	Fermentation / energy metabolism	6	6	23.076923076923077	15.5	22
OD_fatty_acids	Lipid / fatty-acid metabolism	6	3	11.538461538461538	7.333333333333333	11
Sulfate2PAPS	Sulfur metabolism	5	5	19.230769230769234	4.8	6
putative_2-oxoglutarate_ferredoxin_oxidoreductase	Redox / nitrogen metabolism	5	5	19.230769230769234	3.4	7
GR_fatty_acids	Lipid / fatty-acid metabolism	4	4	15.384615384615385	11.75	14
Flavoenzyme_lipids_catabolism	Lipid / fatty-acid metabolism	4	2	7.6923076923076925	13.25	16
Adenylylsulfate_reductase	Sulfur metabolism	3	3	11.538461538461538	11.0	12
Glycine_reductase	Amino-acid metabolism	3	3	11.538461538461538	11.333333333333334	12
OD_AA_metabolism	Amino-acid metabolism	3	3	11.538461538461538	9.0	12
Respiratory_glycerol	Other / unassigned	3	3	11.538461538461538	8.0	10
TPP_fatty_acids	Lipid / fatty-acid metabolism	3	3	11.538461538461538	9.0	11

gallic_acid_met	Aromatic / phenolic metabolism	3	3	11.538461538461538	6.0	8
Arginine2_Hcarbonate	Amino-acid metabolism	3	2	7.6923076923076925	7.0	8
Ech_complex	Other / unassigned	2	2	7.6923076923076925	9.0	12
Lysine_degradation	Amino-acid metabolism	2	2	7.6923076923076925	13.5	15
Nitrate_reductase	Redox / nitrogen metabolism	2	2	7.6923076923076925	9.5	10
aminobutyrate2Butyrate	Fermentation / energy metabolism	2	2	7.6923076923076925	8.0	10
fatty_acids-unassigned	Lipid / fatty-acid metabolism	2	2	7.6923076923076925	13.5	17
proline2aminovalerate	Amino-acid metabolism	2	2	7.6923076923076925	9.5	12
Anaerobic_sulfite_redutase	Other / unassigned	1	1	3.8461538461538463	10.0	10
Bilirubin_reductase	Other / unassigned	1	1	3.8461538461538463	7.0	7
Flavoenzyme_AA_peptides_catabolism	Other / unassigned	1	1	3.8461538461538463	8.0	8
Flavoenzyme_sugar_catabolism	Other / unassigned	1	1	3.8461538461538463	6.0	6

GR_eut-pdu-related	Other / unassigned	1	1	3.8461538461538463	17.0	17
Leucine_reduction	Other / unassigned	1	1	3.8461538461538463	11.0	11
OD_lactate_related	Other / unassigned	1	1	3.8461538461538463	5.0	5
OD_unknown	Other / unassigned	1	1	3.8461538461538463	8.0	8
caffeate_respiration	Other / unassigned	1	1	3.8461538461538463	11.0	11
glutamate2butyric	Other / unassigned	1	1	3.8461538461538463	13.0	13
uric_acid2SCFA	Other / unassigned	1	1	3.8461538461538463	23.0	23

Abbreviations: MGC, metabolic gene cluster; CDS, coding sequence; gutSMASH, gut-specific Specialized Metabolite Analysis Shell; HGD, 2-hydroxyglutarate dehydrogenase-related/unassigned category; Rnf, Rhodobacter nitrogen fixation complex; PFOR, pyruvate:ferredoxin oxidoreductase; TPP, thiamine pyrophosphate-dependent enzyme-related pathway; PAPS, 3′-phosphoadenosine-5′-phosphosulfate; AA, amino acid; OD, oxidoreductase; GR, glycyl radical enzyme-related; SCFA, short-chain fatty acid.

Table S10 C. gutSMASH MGC summary by analysis role.

Analysis role	Bacteria_n	MGC_count_mean	MGC_count_median	MGCs_per_1000_mean	MGCs_per_1000_median	Unique_products_mean	Unique_categories_mean
Contradictory/functional comparator	1	10.0	10.0	3.66	3.66	10.0	6.0
Main OP/low-BMD candidate	12	11.91	11.5	3.72	3.27	10.0	4.83
Normal-BMD-associated comparator	9	12.0	11.0	4.19	4.77	10.66	5.33
Secondary OP candidate	4	7.5	7.0	3.14	3.15	7.0	5.25

Abbreviations: MGC, metabolic gene cluster; OP, osteoporosis; BMD, bone mineral density; gutSMASH, gut-specific Specialized Metabolite Analysis Shell.

Table S10 D. gutSMASH MGC category matrix.

Bacterium	Fermentation / energy metabolism	Amino-acid metabolism	Redox / nitrogen metabolism	Aromatic / phenolic metabolism	Lipid / fatty-acid metabolism	Other / unassigned	Polyamine metabolism	Sulfur metabolism
Eggerthella lenta	3	3	12	2	3	1	0	0
Oscillibacter valericigenes	7	1	2	2	6	4	1	0
Coprococcus comes	7	3	1	4	1	0	0	0
Dorea longicatena	7	3	1	4	0	1	0	0
Dorea formicigenerans	8	2	2	2	1	1	0	0
Odoribacter splanchnicus	9	2	1	0	0	1	1	1
Butyricimonas virosa	8	2	0	0	2	1	0	0
Megamonas hypermegale	6	2	1	1	1	0	1	0
Coprobacter fastidiosus	6	2	1	1	0	1	0	1

Bacteroides fragilis	8	1	1	0	1	0	0	1
Megamonas funiformis	5	2	1	1	1	0	1	0
Bacteroides uniformis	8	1	1	0	0	0	0	1
Coprococcus eutactus	6	1	1	1	0	0	0	1
Ruminococcus torques	6	1	1	1	0	1	0	0
Faecalibacterium prausnitzii	4	1	1	2	0	1	1	0
Parabacteroides distasonis	6	1	0	0	1	1	0	1
Actinomyces naeslundii	4	2	1	1	0	0	1	0
Roseburia intestinalis	5	0	1	1	0	0	1	1
Barnesiella intestinihominis	6	1	0	1	0	0	0	0
Agathobacter rectalis	3	0	1	2	0	0	1	1

Actinomyces odontolyticus	3	1	1	1	0	1	0	0
Catenibacterium mitsuokai	2	1	1	1	1	0	1	0
Turicibacter sanguinis	3	1	0	1	0	1	1	0
Dialister succinatiphilus	1	0	1	1	1	1	1	0
Sutterella wadsworthensis	2	1	1	1	0	0	0	0
Dialister invisus	3	0	0	1	0	0	0	0

Abbreviations: MGC, metabolic gene cluster. Categories were assigned from gutSMASH product annotations into broad functional groups for manuscript-level summarization.
