# Supplementary material for: Genome-Resolved Functional Profiling of Osteoporosis-Associated Gut Bacteria Highlights Putative Metabolic and Immunogenic Signatures of the Gut–Bone Axis
Source: Cells. 2026 Aug 29;15(17):1573. doi: 10.3390/cells15171573 (PMC13565464; doi:10.3390/cells15171573)
Supplement: Supplementary file 1 [file cells-15-01573-s001.zip › Supplementary_Tables/Table S11.pdf]

Table S11 A. Sensitivity analysis of functional rankings

Bacterium	Analysis role	Absolute gut-bone hits	Detected signatures, n	Normalized gut-bone burden	CAZyme burden	KEGG burden	MGC burden	Global integrated score	Rank: Absolute gut-bone hits	Rank: Normalized gut-bone burden	Rank: CAZyme burden	Rank: KEGG burden	Rank: MGC burden	Rank: Global integrated score
Megamonas hypermegale	Normal-BMD-associated comparator	135	11	59.580	26.040	443.954	5.296	0.791	11	4	10	1	4	1
Megamonas funiformis	Normal-BMD-associated comparator	140	11	60.710	21.680	434.085	4.770	0.654	9	3	16	3	7	2
Oscillibacter valericigenes	Main OP/low-BMD candidate	160	11	39.430	9.610	325.283	5.668	0.535	6	18	25	11	2	3
Eggerthella lenta	Main OP/low-BMD candidate	99	12	33.040	8.340	340.120	8.011	0.533	23	25	26	8	1	4
Coprobacter fastidiosus	Main OP/low-BMD candidate	114	11	44.780	43.990	311.076	4.713	0.342	16	9	2	16	8	5
Coprococcus comes	Normal-BMD-associated comparator	114	11	38.000	21.000	324.667	5.333	0.266	16	21	17	12	3	6
Odoribacter splanchnicus	Main OP/low-BMD candidate	161	12	46.690	17.110	263.341	4.350	0.251	5	7	19	22	9	7
Dorea formicigenerans	Normal-BMD-associated comparator	133	12	40.400	15.800	326.245	4.860	0.213	12	14	20	10	6	8
Dorea longicatena	Normal-BMD-associated comparator	125	11	39.440	17.990	292.521	5.049	0.134	13	17	18	20	5	9

Dialister succinatiphilus	Main OP/low-BMD candidate	138	12	73.400	10.640	377.660	3.191	0.123	10	2	24	4	17	10
Dialister invisus	Main OP/low-BMD candidate	121	11	75.020	14.260	376.937	2.480	0.064	14	1	21	5	21	11
Barnesiella intestinihominis	Main OP/low-BMD candidate	106	12	44.520	47.880	297.774	3.360	-0.011	19	11	1	19	15	12
Butyricimonas virosa	Main OP/low-BMD candidate	148	12	40.210	13.310	229.014	3.532	-0.037	8	15	22	26	13	13
Coprococcus eutactus	Normal-BMD-associated comparator	100	10	39.810	31.450	315.685	3.981	-0.137	22	16	6	15	10	14
Roseburia intestinalis	Normal-BMD-associated comparator	179	10	46.340	37.540	305.462	2.330	-0.162	2	8	4	18	24	15
Bacteroides fragilis	Main OP/low-BMD candidate	163	12	35.440	31.090	235.703	2.609	-0.209	4	22	7	25	20	16
Ruminococcus torques	Normal-BMD-associated comparator	101	10	38.760	23.790	322.333	3.837	-0.229	20	19	13	13	11	17
Bacteroides uniformis	Main OP/low-BMD candidate	160	12	35.070	40.110	241.122	2.411	-0.238	6	23	3	24	22	18
Actinomyces naeslundii	Secondary OP candidate	101	12	38.700	24.520	335.249	3.448	-0.252	20	20	11	9	14	19
Faecalibacterium prausnitzii	Contradictory/functional comparator	115	9	42.110	21.970	309.777	3.662	-0.270	15	12	15	17	12	20

Sutterella wadsworthensis	Main OP/low-BMD candidate	114	10	49.140	12.070	434.914	2.155	-0.280	16	6	23	2	26	21
Parabacteroides distasonis	Main OP/low-BMD candidate	192	12	41.530	28.340	254.380	2.163	-0.289	1	13	8	23	25	22
Actinomyces odontolyticus	Secondary OP candidate	93	11	44.630	23.510	321.977	3.359	-0.351	24	10	14	14	16	23
Agathobacter rectalis	Normal-BMD-associated comparator	169	11	49.470	24.300	287.471	2.342	-0.370	3	5	12	21	23	24
Catenibacterium mitsuokai	Secondary OP candidate	69	10	29.080	32.870	357.775	2.950	-0.506	26	26	5	6	18	25
Turicibacter sanguinis	Secondary OP candidate	87	10	34.930	27.700	345.243	2.810	-0.566	25	24	9	7	19	26

Abbreviations: CAZyme, carbohydrate-active enzyme; KEGG, Kyoto Encyclopedia of Genes and Genomes; MGC, metabolic gene cluster; OP, osteoporosis; BMD, bone mineral density; gutSMASH, gut-specific Specialized Metabolite Analysis Shell.

Table S11 B. Correlation and top-five overlap summary

**Metric 1**	**Metric 2**	**Spearman rho**
Global integrated score	MGC burden	0.768
Detected signatures, n	Global integrated score	0.294
Global integrated score	Metabolic burden	0.245
Global integrated score	Normalized gut-bone burden	0.244
Global integrated score	KEGG burden	0.212
Absolute gut-bone hits	Global integrated score	0.194
Global integrated score	Immunogenic burden	0.190
CAZyme burden	Global integrated score	-0.351

Abbreviations: CAZyme, carbohydrate-active enzyme; KEGG, Kyoto Encyclopedia of Genes and Genomes; MGC, metabolic gene cluster; rho, Spearman's rank correlation coefficient.

Table S11 C. Top-5 overlap between individual functional metrics and the global integrated functional score

**Metric compared with global integrated score**	**Top-5 overlap, n**	**Top-5 overlap bacteria**
Absolute gut-bone hits	0	
Normalized gut-bone burden	2	Megamonas funiformis; Megamonas hypermegale
CAZyme burden	1	Coprobacter fastidiosus
KEGG burden	2	Megamonas funiformis; Megamonas hypermegale
MGC burden	3	Eggerthella lenta; Megamonas hypermegale; Oscillibacter valericigenes

Abbreviations: CAZyme, carbohydrate-active enzyme; KEGG, Kyoto Encyclopedia of Genes and Genomes; MGC, metabolic gene cluster.

**Table S11 D.** Leave-one-component-out sensitivity of the equal-weight integrated ranking					
**Omitted component**	**Components retained, n**	**Spearman ρ vs equal-weight reference**	**p value**	**Top-5 overlap**	**Top-5 overlap bacteria**
Fermentation/energy MGCs	9	0.905	<0.001	4/5	*Coprobacter fastidiosus; Eggerthella lenta; Megamonas funiformis; Megamonas hypermegale*
Amino-acid MGCs	9	0.910	<0.001	3/5	*Coprobacter fastidiosus; Megamonas funiformis; Megamonas hypermegale*
CAZyme burden	9	0.923	<0.001	4/5	*Eggerthella lenta; Megamonas funiformis; Megamonas hypermegale; Odoribacter splanchnicus*
Unique MGC products	9	0.931	<0.001	4/5	*Coprobacter fastidiosus; Eggerthella lenta; Megamonas funiformis; Megamonas hypermegale*
MGC burden	9	0.945	<0.001	4/5	*Coprobacter fastidiosus; Megamonas funiformis; Megamonas hypermegale; Odoribacter splanchnicus*
Normalized gut–bone burden	9	0.960	<0.001	4/5	*Coprobacter fastidiosus; Eggerthella lenta; Megamonas funiformis; Megamonas hypermegale*
Metabolic burden	9	0.963	<0.001	5/5	*Coprobacter fastidiosus; Eggerthella lenta; Megamonas funiformis; Megamonas hypermegale; Odoribacter splanchnicus*
Immunogenic burden	9	0.964	<0.001	4/5	*Coprobacter fastidiosus; Eggerthella lenta; Megamonas funiformis; Megamonas hypermegale*
Redox/nitrogen MGCs	9	0.971	<0.001	4/5	*Coprobacter fastidiosus; Megamonas funiformis; Megamonas hypermegale; Odoribacter splanchnicus*

KEGG burden	9	0.971	<0.001	5/5	*Coprobacter fastidiosus; Eggerthella lenta; Megamonas funiformis; Megamonas hypermegale; Odoribacter splanchnicus*

**Table S11 E.** Alternative-weight sensitivity of the integrated ranking						
**Emphasized component**	**Focal weight**	**Weight per remaining component**	**Spearman ρ vs equal-weight reference**	**p value**	**Top-5 overlap**	**Top-5 overlap bacteria**
Immunogenic burden	20.00%	8.89%	0.904	<0.001	4/5	*Coprobacter fastidiosus; Megamonas funiformis; Megamonas hypermegale; Odoribacter splanchnicus*
KEGG burden	20.00%	8.89%	0.925	<0.001	4/5	*Coprobacter fastidiosus; Eggerthella lenta; Megamonas funiformis; Megamonas hypermegale*
Normalized gut–bone burden	20.00%	8.89%	0.932	<0.001	3/5	*Coprobacter fastidiosus; Megamonas funiformis; Megamonas hypermegale*
CAZyme burden	20.00%	8.89%	0.936	<0.001	4/5	*Coprobacter fastidiosus; Megamonas funiformis; Megamonas hypermegale; Odoribacter splanchnicus*
Metabolic burden	20.00%	8.89%	0.937	<0.001	3/5	*Coprobacter fastidiosus; Megamonas funiformis; Megamonas hypermegale*
Fermentation/energy MGCs	20.00%	8.89%	0.951	<0.001	4/5	*Coprobacter fastidiosus; Megamonas funiformis; Megamonas hypermegale; Odoribacter splanchnicus*
Amino-acid MGCs	20.00%	8.89%	0.962	<0.001	4/5	*Coprobacter fastidiosus; Eggerthella lenta; Megamonas funiformis; Megamonas hypermegale*
Unique MGC products	20.00%	8.89%	0.964	<0.001	4/5	*Coprobacter fastidiosus; Megamonas funiformis; Megamonas hypermegale; Odoribacter splanchnicus*
MGC burden	20.00%	8.89%	0.981	<0.001	4/5	*Coprobacter fastidiosus; Eggerthella lenta; Megamonas funiformis; Megamonas hypermegale*

Redox/nitrogen MGCs	20.00%	8.89%	0.992	<0.001	5/5	*Coprobacter fastidiosus; Eggerthella lenta; Megamonas funiformis; Megamonas hypermegale; Odoribacter splanchnicus*

Sensitivity-analysis note: The equal-weight reference was calculated as the arithmetic mean of z-standardized normalized gut–bone burden, metabolic burden, immunogenic burden, CAZyme burden, KEGG burden, MGC burden, unique MGC products, fermentation/energy MGCs, amino-acid MGCs, and redox/nitrogen MGCs. The explicit equal-weight reconstruction was highly concordant with the global integrated ranking reported in Panels A–C (Spearman $\rho = 0.973$, $p < 0.001$; top-five overlap = 4/5: Coprobacter fastidiosus; Eggerthella lenta; Megamonas funiformis; Megamonas hypermegale). In Panel D, each component was omitted sequentially and the remaining nine components received equal weight. In Panel E, the emphasized component received 20% of the total weight and each of the remaining nine components received 8.89%. Spearman correlations and top-five overlap were calculated relative to the explicit equal-weight reference. These scores are comparative rankings and should not be interpreted as biological effect-size estimates. Abbreviations: CAZyme, carbohydrate-active enzyme; KEGG, Kyoto Encyclopedia of Genes and Genomes; MGC, metabolic gene cluster; ρ, Spearman's rank correlation coefficient.
