# Supplementary material for: Genome-Resolved Functional Profiling of Osteoporosis-Associated Gut Bacteria Highlights Putative Metabolic and Immunogenic Signatures of the Gut–Bone Axis
Source: Cells. 2026 Aug 29;15(17):1573. doi: 10.3390/cells15171573 (PMC13565464; doi:10.3390/cells15171573)
Supplement: Supplementary file 1 [file cells-15-01573-s001.zip › Supplementary_Tables/Table S12.pdf]

Table S12. Integrated functional profile classification

Bacterium	Analysis role	Gut-bone hits per 1,000 proteins	Metabolic hits per 1,000 proteins	Immunogenic hits per 1,000 proteins	CAZyme hits per 1,000 proteins	Selected KEGG pathway hits per 1,000 proteins	MGCs, n	MGCs per 1,000 proteins	Unique MGC products, n	Integrated metabolic/MGC score	Integrated immunogenic score	Global integrated score	Integrated profile class	Interpretation flag
Megamonas hypermegale	Normal-BMD-associated comparator	59.580	44.570	15.000	26.040	443.954	12	5.296	12	0.978	0.436	0.791	Metabolic/MGC-enriched integrated profile	High relative metabolic, CAZyme, KEGG and/or MGC burden
Megamonas funiformis	Normal-BMD-associated comparator	60.710	45.530	15.180	21.680	434.085	11	4.770	11	0.743	0.471	0.654	Metabolic/MGC-enriched integrated profile	High relative metabolic, CAZyme, KEGG and/or MGC burden
Oscillibacter valericigenes	Main OP/low-BMD candidate	39.430	24.640	14.790	9.610	325.283	23	5.668	16	0.222	0.394	0.535	High global integrated potential	Intermediate relative integrated functional potential
Eggerthella lenta	Main OP/low-BMD candidate	33.040	26.370	6.680	8.340	340.120	24	8.011	8	0.212	-1.197	0.533	High global integrated potential	Intermediate relative integrated functional potential
Coprobacter fastidiosus	Main OP/low-BMD candidate	44.780	28.670	16.100	43.990	311.076	12	4.713	12	0.544	0.651	0.342	Mixed high metabolic–immunogenic potential	High relative potential across metabolic/MGC and immunogenic axes
Coprococcus comes	Normal-BMD-associated comparator	38.000	29.670	8.330	21.000	324.667	16	5.333	13	0.619	-0.874	0.266	Metabolic/MGC-enriched integrated profile	High relative metabolic, CAZyme,

														KEGG and/or MGC burden
Odoribacter splanchnicus	Main OP/low-BMD candidate	46.690	31.320	15.370	17.110	263.341	15	4.350	15	0.396	0.508	0.251	Mixed high metabolic–immunogenic potential	High relative potential across metabolic/MGC and immunogenic axes
Dorea formicigenerans	Normal-BMD-associated comparator	40.400	32.810	7.590	15.800	326.245	16	4.860	13	0.460	-1.019	0.213	Metabolic/MGC-enriched integrated profile	High relative metabolic, CAZyme, KEGG and/or MGC burden
Dorea longicatena	Normal-BMD-associated comparator	39.440	29.980	9.470	17.990	292.521	16	5.049	13	0.472	-0.650	0.134	Metabolic/MGC-enriched integrated profile	High relative metabolic, CAZyme, KEGG and/or MGC burden
Dialister succinatiphilus	Main OP/low-BMD candidate	73.400	52.660	20.740	10.640	377.660	6	3.191	6	-0.412	1.562	0.123	Immunogenic-pattern-enriched integrated profile	High relative structural immunogenic signature burden
Dialister invisus	Main OP/low-BMD candidate	75.020	52.080	22.940	14.260	376.937	4	2.480	4	-0.411	1.994	0.064	Immunogenic-pattern-enriched integrated profile	High relative structural immunogenic signature burden
Barnesiella intestinihominis	Main OP/low-BMD candidate	44.520	29.400	15.120	47.880	297.774	8	3.360	8	0.085	0.459	-0.011	Intermediate integrated profile	Intermediate relative integrated functional potential
Butyricimonas virosa	Main OP/low-BMD candidate	40.210	27.440	12.770	13.310	229.014	13	3.532	13	-0.057	-0.002	-0.037	Intermediate integrated profile	Intermediate relative integrated

													functional potential
Coprococcus eutactus	Normal-BMD-associated comparator	39.810	29.460	10.350	31.450	315.685	10	3.981	9	0.016	-0.477	-0.137	Intermediate integrated profile	Intermediate relative integrated functional potential
Roseburia intestinalis	Normal-BMD-associated comparator	46.340	25.890	20.450	37.540	305.462	9	2.330	9	-0.391	1.505	-0.162	Intermediate integrated profile	Intermediate relative integrated functional potential
Bacteroides fragilis	Main OP/low-BMD candidate	35.440	25.880	9.570	31.090	235.703	12	2.609	12	-0.129	-0.630	-0.209	Intermediate integrated profile	Intermediate relative integrated functional potential
Ruminococcus torques	Normal-BMD-associated comparator	38.760	29.160	9.590	23.790	322.333	10	3.837	9	-0.093	-0.626	-0.229	Intermediate integrated profile	Intermediate relative integrated functional potential
Bacteroides uniformis	Main OP/low-BMD candidate	35.070	24.990	10.080	40.110	241.122	11	2.411	11	-0.074	-0.530	-0.238	Intermediate integrated profile	Intermediate relative integrated functional potential
Actinomyces naeslundii	Secondary OP candidate	38.700	31.420	7.280	24.520	335.249	9	3.448	8	-0.064	-1.080	-0.252	Intermediate integrated profile	Intermediate relative integrated functional potential
Faecalibacterium prausnitzii	Contradictory/functional comparator	42.110	33.320	8.790	21.970	309.777	10	3.662	10	-0.178	-0.783	-0.270	Low integrated functional potential	Lower relative burden across integrated functional dimensions

Sutterella wadsworthensis	Main OP/low-BMD candidate	49.140	29.740	19.400	12.070	434.914	5	2.155	5	-0.583	1.299	-0.280	Low integrated functional potential	Lower relative burden across integrated functional dimensions
Parabacteroides distasonis	Main OP/low-BMD candidate	41.530	30.720	10.820	28.340	254.380	10	2.163	10	-0.304	-0.385	-0.289	Low integrated functional potential	Lower relative burden across integrated functional dimensions
Actinomyces odontolyticus	Secondary OP candidate	44.630	37.910	6.720	23.510	321.977	7	3.359	7	-0.281	-1.190	-0.351	Low integrated functional potential	Lower relative burden across integrated functional dimensions
Agathobacter rectalis	Normal-BMD-associated comparator	49.470	26.350	23.130	24.300	287.471	8	2.342	7	-0.834	2.031	-0.370	Low integrated functional potential	Lower relative burden across integrated functional dimensions
Catenibacterium mitsuokai	Secondary OP candidate	29.080	21.070	8.010	32.870	357.775	7	2.950	7	-0.470	-0.936	-0.506	Low integrated functional potential	Lower relative burden across integrated functional dimensions
Turicibacter sanguinis	Secondary OP candidate	34.930	26.900	8.030	27.700	345.243	7	2.810	6	-0.465	-0.932	-0.566	Low integrated functional potential	Lower relative burden across integrated functional dimensions
