# Supplementary material for: Genome-Resolved Functional Profiling of Osteoporosis-Associated Gut Bacteria Highlights Putative Metabolic and Immunogenic Signatures of the Gut–Bone Axis
Source: Cells. 2026 Aug 29;15(17):1573. doi: 10.3390/cells15171573 (PMC13565464; doi:10.3390/cells15171573)
Supplement: Supplementary file 1 [file cells-15-01573-s001.zip › Supplementary_Tables/Table S2.pdf]

Table S2. Curated feature-level evidence summary

Functional signature	Feature ID	Preferred name	KEGG KO	EC	Evidence tier	Genomes detected	Proteins detected	Representative description
Bile acid-related	cbh	cbh	ko:K01442	3.5.1.24	Tier 1	13	19	Choloylglycine hydrolase
Bile acid-related	ko:K01442	-	ko:K01442	3.5.1.24	Tier 1	5	10	Linear amide C-N hydrolases, choloylglycine hydrolase family
Bile acid-related	ko:K03453	-	ko:K03453	-	Tier 1	4	5	Sodium bile acid symporter family
Bile acid-related	ko:K07088	-	ko:K07088	-	Tier 1	1	2	Sodium Bile acid symporter family
Bile acid-related	Sodium Bile acid symporter family	-	-	-	Tier 1	1	1	Sodium Bile acid symporter family
Bile acid-related	pacB	pacB	ko:K01442	3.5.1.24	Tier 1	1	1	Choloylglycine hydrolase
Bile acid-related	ybaS	ybaS	ko:K00041,ko:K03453	1.1.1.58	Tier 1	1	1	Sodium Bile acid symporter family
Flagella/TLR5	Putative Flagellin, Flp1-like, domain	-	-	-	Tier 1	7	7	Putative Flagellin, Flp1-like, domain

Flagella/TLR5	fliU	fliU	ko:K18475	-	Tier 1	7	7	N-methylation of lysine residues in flagellin K00599
Flagella/TLR5	fliD	fliD	ko:K02407	-	Tier 1	4	12	Required for morphogenesis and for the elongation of the flagellar filament by facilitating polymerization of the flagellin monomers at the tip of growing filament. Forms a capping structure, which prevents flagellin sub
Flagella/TLR5	fliC	fliC	ko:K02406	-	Tier 1	3	9	Flagellin is the subunit protein which polymerizes to form the filaments of

								bacterial flagella
Flagella/TLR5	flgK	flgK	ko:K02396	-	Tier 1	3	5	TIGRFAM Flagellar hook-associated protein, FlgK
Flagella/TLR5	fliS	fliS	ko:K02422	-	Tier 1	3	5	Flagellar protein FliS
Flagella/TLR5	fliN	fliN	ko:K02417	-	Tier 1	3	4	CheC-like family
Flagella/TLR5	flg	flg	-	-	Tier 1	3	3	TIGRFAM flagellar operon protein
Flagella/TLR5	flgC	flgC	ko:K02388	-	Tier 1	3	3	Belongs to the flagella basal body rod proteins family
Flagella/TLR5	flgD	flgD	ko:K02389	-	Tier 1	3	3	Required for flagellar hook formation. May act as a scaffolding protein
Flagella/TLR5	flgE	flgE	ko:K02390	-	Tier 1	3	3	basal body rod protein
Flagella/TLR5	flgG	flgG	ko:K02390,ko:K02392	-	Tier 1	3	3	basal body rod protein
Flagella/TLR5	flgL	flgL	ko:K02397	-	Tier 1	3	3	Belongs to the bacterial flagellin family

Flagella/TLR5	flhA	flhA	ko:K02400	-	Tier 1	3	3	Required for formation of the rod structure of the flagellar apparatus. Together with FliI and FliH, may constitute the export apparatus of flagellin
Flagella/TLR5	flhB	flhB	ko:K02401,ko:K03229,ko:K04061,ko:K13820,ko:K22510	-	Tier 1	3	3	Required for formation of the rod structure in the basal body of the flagellar apparatus. Together with FliI and FliH, may constitute the export apparatus of flagellin
Flagella/TLR5	flhO	flhO	ko:K02388,ko:K02391,ko:K02392	-	Tier 1	3	3	basal body rod protein
Flagella/TLR5	fliA	fliA	ko:K02405	-	Tier 1	3	3	Sigma factors are initiation

								factors that promote the attachment of RNA polymerase to specific initiation sites and are then released
Flagella/TLR5	fliE	fliE	ko:K02408	-	Tier 1	3	3	Flagellar hook-basal body complex protein FliE
Flagella/TLR5	fliH	fliH	ko:K02411,ko:K03223	-	Tier 1	3	3	Flagellar assembly protein FliH
Flagella/TLR5	fliJ	fliJ	ko:K02413	-	Tier 1	3	3	flagellar export protein FliJ
Flagella/TLR5	fliK	fliK	ko:K02414	-	Tier 1	3	3	Flagellar hook-length control protein FliK
Flagella/TLR5	fliW	fliW	ko:K13626	-	Tier 1	3	3	Binds to the C-terminal region of flagellin, which is implicated in polymerization, and participates in the assembly

								of the flagellum
Flagella/TLR5	fliZ	fliZ	ko:K02418	-	Tier 1	3	3	Flagellar biosynthesis protein, FliO
Flagella/TLR5	ko:K02396, ko:K18827	-	ko:K02396,ko:K18827	2.1.1.294,2.7.1.181	Tier 1	3	3	bacterial-type flagellum assembly
Flagella/TLR5	Flagellar hook-length control protein FliK	-	-	-	Tier 1	2	2	Flagellar hook-length control protein FliK
Flagella/TLR5	flaG	flaG	ko:K06603	-	Tier 1	2	2	PFAM Flagellar protein FlaG protein
Flagella/TLR5	flbD	flbD	ko:K02385	-	Tier 1	2	2	Flagellar protein (FlbD)
Flagella/TLR5	flgB	flgB	ko:K02387	-	Tier 1	2	2	Structural component of flagellum, the bacterial motility apparatus. Part of the rod structure of flagellar basal body

Flagella/TLR5	flgJ	flgJ	ko:K02395,ko:K08309	-	Tier 1	2	2	Flagellar rod assembly protein muramidase FlgJ
Flagella/TLR5	flhB	flhB	ko:K02401,ko:K13820	-	Tier 1	2	2	Required for formation of the rod structure in the basal body of the flagellar apparatus. Together with FliI and FliH, may constitute the export apparatus of flagellin
Flagella/TLR5	flhB2	flhB2	ko:K04061	-	Tier 1	2	2	FlhB HrpN YscU SpaS Family
Flagella/TLR5	fliG	fliG	ko:K02410	-	Tier 1	2	2	Flagellar motor switch protein flig
Flagella/TLR5	fliI	fliI	ko:K02412,ko:K03224	3.6.3.14	Tier 1	2	2	Flagellar protein export ATPase FliI
Flagella/TLR5	fliM	fliM	ko:K02416	-	Tier 1	2	2	flagellar motor switch protein FliM

Flagella/TLR5	fliQ	fliQ	ko:K02420,ko:K03227	-	Tier 1	2	2	Flagellar biosynthetic protein FliQ
Flagella/TLR5	fliR	fliR	ko:K02421,ko:K03228,ko:K13820	-	Tier 1	2	2	flagellar biosynthetic protein FliR
Flagella/TLR5	ko:K02407	-	ko:K02407	-	Tier 1	2	2	Required for morphogenesis and for the elongation of the flagellar filament by facilitating polymerization of the flagellin monomers at the tip of growing filament. Forms a capping structure, which prevents flagellin sub
Flagella/TLR5	ko:K02415	-	ko:K02415	-	Tier 1	2	2	Controls the rotational direction of flagella during chemotaxis

Flagella/TLR5	ko:K18475	-	ko:K18475	-	Tier 1	2	2	N-methylation of lysine residues in flagellin K00599
Flagella/TLR5	pilZ	pilZ	-	-	Tier 1	2	2	Flagellar protein YcgR
Flagella/TLR5	yvyF	yvyF	-	-	Tier 1	2	2	TIGRFAM flagellar operon protein
Flagella/TLR5	ko:K09749	-	ko:K09749	-	Tier 1	1	2	Flagellar Assembly Protein A
Flagella/TLR5	Flagellar motor protein MotA	-	-	-	Tier 1	1	1	Flagellar motor protein MotA
Flagella/TLR5	flgB	flgB	ko:K02387,ko:K02388	-	Tier 1	1	1	bacterial-type flagellum-dependent cell motility
Flagella/TLR5	fliF	fliF	ko:K02409	-	Tier 1	1	1	Flagellar M-ring protein C-terminal
Flagella/TLR5	fliM	fliM	ko:K02416,ko:K02417	-	Tier 1	1	1	Flagellar motor switch protein FliM
Flagella/TLR5	ko:K09749, ko:K13924	-	ko:K09749,ko:K13924	2.1.1.80,3.1.1.61	Tier 1	1	1	Flagellar Assembly Protein A

Flagella/TLR5	motB	motB	ko:K02557	-	Tier 1	1	1	COGs COG1360 Flagellar motor protein
Flagella/TLR5	porU	porU	-	-	Tier 2	1	1	bacterial-type flagellum-dependent cell motility
Folate/one-carbon	folC	folC	ko:K11754	6.3.2.12,6.3.2.17	Tier 1	26	33	Bifunctional protein FolC
Folate/one-carbon	glyA	glyA	ko:K00600	2.1.2.1	Tier 1	26	31	Catalyzes the reversible interconversion of serine and glycine with tetrahydrofolate (THF) serving as the one-carbon carrier. This reaction serves as the major source of one-carbon groups required for the biosynthesis of
Folate/one-carbon	folD	folD	ko:K01491	1.5.1.5,3.5.4.9	Tier 1	26	27	Catalyzes the oxidation of 5,10-methylenetetra

								hydrofolate to 5,10-methenyltetrahydrofolate and then the hydrolysis of 5,10-methenyltetrahydrofolate to 10-formyltetrahydrofolate
Folate/one-carbon	thyA	thyA	ko:K00560	2.1.1.45	Tier 1	20	21	Catalyzes the reductive methylation of 2'-deoxyuridine-5'-monophosphate (dUMP) to 2'-deoxythymidine-5'-monophosphate (dTMP) while utilizing 5,10-methylenetetrahydrofolate (mTHF) as the methyl donor

								and reductant in the
Folate/one-carbon	folP	folP	ko:K00796,ko:K13941	2.5.1.15,2.7.6.3	Tier 1	19	21	Catalyzes the condensation of para-aminobenzoate (pABA) with 6-hydroxymethyl-7,8-dihydropterin diphosphate (DHPt-PP) to form 7,8-dihydropteroate (H2Pte), the immediate precursor of folate derivatives
Folate/one-carbon	purN	purN	ko:K11175,ko:K13713	2.1.2.2,6.3.2.6,6.3.4.13	Tier 1	19	19	Catalyzes the transfer of a formyl group from 10-formyltetrahydrofolate to 5-phospho-ribosyl-glycinamide (GAR),

								producing 5-phospho-ribosyl-N-formylglycinamide (FGAR) and tetrahydrofolate
Folate/one-carbon	ygfA	ygfA	ko:K01934	6.3.3.2	Tier 1	18	18	5-formyltetrahydrofolate cyclo-ligase family
Folate/one-carbon	fhs	fhs	ko:K00288,ko:K01938	1.5.1.5,3.5.4.9,6.3.4.3	Tier 1	16	16	Belongs to the formate--tetrahydrofolate ligase family
Folate/one-carbon	folK	folK	ko:K00950,ko:K13940	2.7.6.3,4.1.2.25	Tier 1	11	15	COG NOG22185 non supervised orthologous group
Folate/one-carbon	folB	folB	ko:K00796,ko:K00950,ko:K01633,ko:K13940	1.13.11.81,2.5.1.15,2.7.6.3,4.1.2.25,5.1.99.8	Tier 1	10	10	Catalyzes the conversion of 7,8-dihydroneopterin to 6-hydroxymethyl-7,8-dihydropterin

Folate/one-carbon	folE	folE	ko:K00950,ko:K01495	2.7.6.3,3.5.4.16	Tier 1	10	10	GTP cyclohydrolase 1
Folate/one-carbon	metF	metF	ko:K00297	1.5.1.20	Tier 1	10	10	Methylenetetrahydrofolate reductase
Folate/one-carbon	folE	folE	ko:K01495	3.5.4.16	Tier 1	9	9	GTP cyclohydrolase I
Folate/one-carbon	trmFO	trmFO	ko:K04094	2.1.1.74	Tier 1	9	9	Catalyzes the folate-dependent formation of 5-methyl- uridine at position 54 (M-5-U54) in all tRNAs
Folate/one-carbon	folA	folA	ko:K00287,ko:K00950	1.5.1.3,2.7.6.3	Tier 1	8	8	Key enzyme in folate metabolism. Catalyzes an essential reaction for de novo glycine and purine synthesis, and for DNA precursor synthesis

Folate/one-carbon	fhs	fhs	ko:K01938	6.3.4.3	Tier 1	7	10	Belongs to the formate--tetrahydrofolate ligase family
Folate/one-carbon	folK	folK	ko:K00796,ko:K00950,ko:K01633,ko:K13940	1.13.11.81,2.5.1.15,2.7.6.3,4.1.2.25,5.1.99.8	Tier 1	7	7	Catalyzes the conversion of 7,8-dihydroneopterin to 6-hydroxymethyl-7,8-dihydropterin
Folate/one-carbon	metF	metF	ko:K00297,ko:K21010	1.5.1.20	Tier 1	7	7	Methylenetetrahydrofolate reductase
Folate/one-carbon	purT	purT	ko:K08289	2.1.2.2	Tier 1	7	7	Involved in the de novo purine biosynthesis. Catalyzes the transfer of formate to 5-phospho-ribosyl-glycinamide (GAR), producing 5-phospho-ribosyl-N-formylglycinamide (FGAR).

Folate/one-carbon	thyX	thyX	ko:K03465	2.1.1.148	Tier 1	5	6	Formate is provided by PurU via hydrolysis of Catalyzes the reductive methylation of 2'-deoxyuridine-5'-monophosphate (dUMP) to 2'-deoxythymidine-5'-monophosphate (dTMP) while utilizing 5,10-methylenetetrahydrofolate (mTHF) as the methyl donor, and NADPH and FADH(2
Folate/one-carbon	fchA	fchA	-	-	Tier 1	5	5	COG3404 Methenyl tetrahydrofolate cyclohydrolase
Folate/one-carbon	folA	folA	ko:K00287,ko:K18589	1.5.1.3	Tier 1	5	5	Key enzyme in folate

								metabolism. Catalyzes an essential reaction for de novo glycine and purine synthesis, and for DNA precursor synthesis
Folate/one-carbon	dihydrofolate reductase family protein K00287	-	-	-	Tier 1	4	8	dihydrofolate reductase family protein K00287
Folate/one-carbon	Key enzyme in folate metabolism. Catalyzes an essential reac	-	-	-	Tier 1	4	5	Key enzyme in folate metabolism. Catalyzes an essential reaction for de novo glycine and purine synthesis, and for DNA precursor synthesis
Folate/one-carbon	folA	folA	ko:K00287	1.5.1.3	Tier 1	4	4	Key enzyme in folate metabolism.

								Catalyzes an essential reaction for de novo glycine and purine synthesis, and for DNA precursor synthesis
Folate/one-carbon	fthC	fthC	ko:K01934	6.3.3.2	Tier 1	4	4	5-formyltetrahydrofolate cyclo-ligase
Folate/one-carbon	purD	purD	ko:K01945,ko:K01955	6.3.4.13,6.3.5.5	Tier 1	4	4	Involved in the de novo purine biosynthesis. Catalyzes the transfer of formate to 5-phospho-ribosyl-glycinamide (GAR), producing 5-phospho-ribosyl-N-formylglycinamide (FGAR). Formate is provided by

Folate/one-carbon	purN	purN	ko:K11175	2.1.2.2	Tier 1	4	4	PurU via hydrolysis of Catalyzes the transfer of a formyl group from 10-formyltetrahydrofolate to 5-phospho-ribosyl-glycinamide (GAR), producing 5-phospho-ribosyl-N-formylglycinamide (FGAR) and tetrahydrofolate
Folate/one-carbon	folA	folA	ko:K00287,ko:K18589,ko:K18590	1.5.1.3	Tier 1	3	3	Key enzyme in folate metabolism. Catalyzes an essential reaction for de novo glycine and purine synthesis, and for DNA

								precursor synthesis
Folate/one-carbon	metE	metE	ko:K00549	2.1.1.14	Tier 1	3	3	Catalyzes the transfer of a methyl group from 5-methyltetrahydrofolate to homocysteine resulting in methionine formation
Folate/one-carbon	metH	metH	ko:K00544,ko:K00548	2.1.1.13,2.1.1.5	Tier 1	3	3	5-methyltetrahydrofolate--homocysteine methyltransferase
Folate/one-carbon	Involved in the de novo purine biosynthesis. Catalyzes the t	-	-	-	Tier 1	2	2	Involved in the de novo purine biosynthesis. Catalyzes the transfer of formate to 5-phospho-ribosyl-glycinamide (GAR), producing 5-phospho-

								ribosyl-N-formylglycinamide (FGAR). Formate is provided by PurU via hydrolysis of
Folate/one-carbon	folA	folA	ko:K00097,ko:K00287,ko:K18589,ko:K18590	1.1.1.262,1.5.1.3	Tier 1	2	2	Key enzyme in folate metabolism. Catalyzes an essential reaction for de novo glycine and purine synthesis, and for DNA precursor synthesis
Folate/one-carbon	folD4	folD4	-	-	Tier 1	2	2	Zn-dependent hydrolases of the beta-lactamase fold
Folate/one-carbon	folK	folK	ko:K00796,ko:K00950,ko:K01495,ko:K01633,ko:K13940,ko:K17488	1.13.11.81,2.5.1.15,2.7.6.3,3.5.4.16,3.5.4.39,4.1.2.25,5.1.99.8	Tier 1	2	2	Catalyzes the conversion of 7,8-dihydroneopterin to 6-hydroxymethyl

								-7,8-dihydropterin
Folate/one-carbon	ko:K17810	-	ko:K17810	6.3.1.12	Tier 1	2	2	Involved in the de novo purine biosynthesis. Catalyzes the transfer of formate to 5-phospho-ribosyl-glycinamide (GAR), producing 5-phospho-ribosyl-N-formylglycinamide (FGAR). Formate is provided by PurU via hydrolysis of
Folate/one-carbon	purU	purU	ko:K00974,ko:K01433	2.7.7.72,3.5.1.10	Tier 1	2	2	Catalyzes the hydrolysis of 10-formyltetrahydrofolate (formyl-FH4) to formate and tetrahydrofolate (FH4)

Folate/one-carbon	purU	purU	ko:K01433	3.5.1.10	Tier 1	2	2	Catalyzes the hydrolysis of 10-formyltetrahydrofolate (formyl-FH4) to formate and tetrahydrofolate (FH4)
Folate/one-carbon	fchA	fchA	ko:K01746	4.3.1.4	Tier 1	1	1	Methenyltetrahydrofolate cyclohydrolase
Folate/one-carbon	fmt	fmt	ko:K00604,ko:K17810	2.1.2.9,6.3.1.12	Tier 1	1	1	Involved in the de novo purine biosynthesis. Catalyzes the transfer of formate to 5-phospho-ribosyl-glycinamide (GAR), producing 5-phospho-ribosyl-N-formylglycinamide (FGAR). Formate is provided by

Folate/one-carbon	folA	folA	ko:K00287,ko:K00560,ko:K05896,ko:K17364	1.5.1.3,1.5.1.47,2.1.1.45	Tier 1	1	1	PurU via hydrolysis of Key enzyme in folate metabolism. Catalyzes an essential reaction for de novo glycine and purine synthesis, and for DNA precursor synthesis
Folate/one-carbon	folB	folB	ko:K00950,ko:K01091,ko:K01633,ko:K13940	1.13.11.81,2.7.6.3,3.1.3.18,4.1.2.25,5.1.99.8	Tier 1	1	1	Psort location Cytoplasmic, score
Folate/one-carbon	folB	folB	ko:K01633	1.13.11.81,4.1.2.25,5.1.99.8	Tier 1	1	1	Dihydroneopterin aldolase
Folate/one-carbon	folK	folK	ko:K00796,ko:K00950,ko:K01495,ko:K13940,ko:K13941	2.5.1.15,2.7.6.3,3.5.4.16,4.1.2.25	Tier 1	1	1	2-amino-4-hydroxy-6-hydroxymethyldihydropteridine diphosphokinase
Folate/one-carbon	folP	folP	ko:K00796,ko:K00950,ko:K01495,ko:K13940,ko:K13941	2.5.1.15,2.7.6.3,3.5.4.16,4.1.2.25	Tier 1	1	1	dihydropteroate synthase

Folate/one-carbon	folP	folP	ko:K00796,ko:K00950,ko:K01633	1.13.11.81,2.5.1.15,2.7.6.3,4.1.2.25,5.1.99.8	Tier 1	1	1	Catalyzes the condensation of para-aminobenzoate (pABA) with 6-hydroxymethyl-7,8-dihydropterin diphosphate (DHPt-PP) to form 7,8-dihydropteroate (H2Pte), the immediate precursor of folate derivatives
Folate/one-carbon	gcvT	gcvT	ko:K00600,ko:K00605,ko:K18847	2.1.2.1,2.1.2.10,2.2.1.8	Tier 1	1	1	Aminomethyltransferase folate-binding domain
Folate/one-carbon	ko:K01905, ko:K11175, ko:K22224	-	ko:K01905,ko:K11175,ko:K22224	2.1.2.2,6.2.1.13	Tier 1	1	1	Catalyzes the transfer of a formyl group from 10-formyltetrahydrofolate to 5-phospho-ribosyl-

								glycinamide (GAR), producing 5-phospho-ribosyl-N-formylglycinamide (FGAR) and tetrahydrofolate
Folate/one-carbon	ko:K03465	-	ko:K03465	2.1.1.148	Tier 1	1	1	Catalyzes the reductive methylation of 2'-deoxyuridine-5'-monophosphate (dUMP) to 2'-deoxythymidine-5'-monophosphate (dTMP) while utilizing 5,10-methylenetetrahydrofolate (mTHF) as the methyl donor, and NADPH and FADH(2

LPS/lipid A	kdsC	kdsC	ko:K03270	3.1.3.45	Tier 1	13	13	3-deoxy-D-manno-octulosonate 8-phosphate phosphatase, YrbI family
LPS/lipid A	lpxA	lpxA	ko:K00677	2.3.1.129	Tier 1	12	20	Involved in the biosynthesis of lipid A, a phosphorylated glycolipid that anchors the lipopolysaccharide to the outer membrane of the cell
LPS/lipid A	kdsB	kdsB	ko:K00979	2.7.7.38	Tier 1	12	13	Activates KDO (a required 8-carbon sugar) for incorporation into bacterial lipopolysaccharide in Gram-negative bacteria
LPS/lipid A	kdsA	kdsA	ko:K01627	2.5.1.55	Tier 1	12	12	Belongs to the KdsA family
LPS/lipid A	lpxB	lpxB	ko:K00748	2.4.1.182	Tier 1	12	12	Condensation of UDP-2,3-

								diacylglucosamine and 2,3-diacylglucosamine-1-phosphate to form lipid A disaccharide, a precursor of lipid A, a phosphorylated glycolipid that anchors the lipopolysaccharide to the outer membrane
LPS/lipid A	lpxD	lpxD	ko:K02536	2.3.1.191	Tier 1	12	12	Catalyzes the N-acylation of UDP-3-O-acylglucosamine using 3-hydroxyacyl-ACP as the acyl donor. Is involved in the biosynthesis of lipid A, a phosphorylated glycolipid that anchors the lipopolysacchar

LPS/lipid A	lptC	lptC	ko:K09774,ko:K11719	-	Tier 1	11	11	ide to the outer mem Lipopolysaccharide-assembly, LptC-related
LPS/lipid A	lpxK	lpxK	ko:K00912	2.7.1.130	Tier 1	10	10	Transfers the gamma-phosphate of ATP to the 4'-position of a tetraacyldisaccharide 1-phosphate intermediate (termed DS-1-P) to form tetraacyldisaccharide 1,4'-bis-phosphate (lipid IVA)
LPS/lipid A	kdsD	kdsD	ko:K06041	5.3.1.13	Tier 1	9	9	sugar phosphate isomerase involved in capsule formation
LPS/lipid A	waaY	waaY	ko:K02850,ko:K03688	-	Tier 1	8	9	unusual protein kinase

LPS/lipid A	lpxH	lpxH	ko:K03269	3.6.1.54	Tier 1	8	8	Psort location Cytoplasmic, score
LPS/lipid A	waaM	waaM	ko:K02517	2.3.1.241	Tier 1	7	8	Lipid A biosynthesis (KDO)2-(Lauroyl)-lipid IVA acyltransferase
LPS/lipid A	fabZ	fabZ	ko:K02372,ko:K02535,ko:K13599,ko:K16363	3.5.1.108,4.2.1.59	Tier 1	7	7	Catalyzes the hydrolysis of UDP-3-O-myristoyl-N-acetylglucosamine to form UDP-3-O-myristoylglucosamine and acetate, the committed step in lipid A biosynthesis
LPS/lipid A	waaA	waaA	ko:K02527,ko:K03439	2.1.1.33,2.4.99.12,2.4.99.13,2.4.99.14,2.4.99.15	Tier 1	7	7	Psort location Cytoplasmic, score 8.96
LPS/lipid A	htrB	htrB	ko:K02517	2.3.1.241	Tier 1	5	9	Lipid A biosynthesis
