# Supplementary material for: Genome-Resolved Functional Profiling of Osteoporosis-Associated Gut Bacteria Highlights Putative Metabolic and Immunogenic Signatures of the Gut–Bone Axis
Source: Cells. 2026 Aug 29;15(17):1573. doi: 10.3390/cells15171573 (PMC13565464; doi:10.3390/cells15171573)
Supplement: Supplementary file 1 [file cells-15-01573-s001.zip › Supplementary_Tables/Table S3.pdf]

**Table S3.** Relationship between the primary candidate annotation set and the strict high-confidence curation subset by functional signature

| Functional signature | Primary candidate hits | Strict high-confidence hits | Not retained in strict subset | Strict-subset retention (%) | Primary genomes detected | Strict-subset genomes detected |
|---|---|---|---|---|---|---|
| Peptidoglycan/cell wall | 520 | 516 | 4 | 99.23 | 26 | 26 |
| Vitamin B12/cobalamin | 690 | 493 | 197 | 71.45 | 26 | 26 |
| Folate/one-carbon | 553 | 367 | 186 | 66.37 | 26 | 26 |
| LPS/lipid A | 267 | 258 | 9 | 96.63 | 21 | 20 |
| SCFA: propionate | 215 | 215 | 0 | 100.00 | 26 | 26 |
| Flagella/TLR5 | 185 | 143 | 42 | 77.30 | 19 | 12 |
| Tryptophan/indole | 133 | 133 | 0 | 100.00 | 26 | 26 |
| Vitamin K/menaquinone | 128 | 128 | 0 | 100.00 | 19 | 19 |
| Polyamines | 118 | 118 | 0 | 100.00 | 25 | 25 |
| SCFA: acetate | 86 | 48 | 38 | 55.81 | 25 | 21 |
| Bile acid-related | 60 | 39 | 21 | 65.00 | 23 | 17 |
| SCFA: butyrate | 382 | 39 | 343 | 10.21 | 26 | 10 |
| **Total** | 3337 | 2497 | 840 | 74.83 | 26 unique genomes | 26 unique genomes |

**Abbreviations**: MGC, metabolic gene cluster. The percentage was calculated as strict high-confidence hits divided by primary candidate hits x 100. Genome counts indicate the number of genomes with at least one hit in the corresponding category. The total row summarizes hit counts and indicates the total number of unique genomes represented across the analysis.
