# Supplementary material for: Genome-Resolved Functional Profiling of Osteoporosis-Associated Gut Bacteria Highlights Putative Metabolic and Immunogenic Signatures of the Gut–Bone Axis
Source: Cells. 2026 Aug 29;15(17):1573. doi: 10.3390/cells15171573 (PMC13565464; doi:10.3390/cells15171573)
Supplement: Supplementary file 1 [file cells-15-01573-s001.zip › Supplementary_Tables/Table S4.pdf]

Table S4 A. Genome-by-signature count and presence/absence matrices for curated gut–bone axis functional signatures

Bacterium	Butyrate	Acetate	Propionate	Vitamin K/ menaquinone	Vitamin B12/ cobalamin	Folate/ one-carbon	Tryptophan/ indole	Bile acid-related	Polyamines	LPS/ lipid A	Flagella/ TLR5	Peptido-glycan	Total curated hits
Parabacteroides distasonis	12	4	11	9	34	50	9	8	5	27	1	22	192
Roseburia intestinalis	20	2	3	0	36	18	9	3	9	0	55	24	179
Agathobacter rectalis	18	2	4	0	34	14	7	2	9	3	58	18	169
Bacteroides fragilis	14	4	10	10	36	35	7	1	2	18	1	25	163
Odoribacter splanchnicus	15	6	13	11	30	18	8	3	4	26	6	21	161
Bacteroides uniformis	13	4	7	7	40	29	8	4	2	21	4	21	160
Oscillibacter valericigenes	25	2	5	2	25	29	6	1	5	0	36	24	160
Butyricimonas virosa	18	5	14	11	22	17	4	6	4	26	3	18	148
Megamonas funiformis	14	6	16	4	31	20	5	2	7	18	0	17	140
Dialister succinatiphilus	12	2	12	2	33	22	5	1	10	19	1	19	138

Megamonas hypermegale	14	5	14	5	26	20	6	2	9	17	0	17	135
Dorea formicigenerans	15	5	4	6	45	24	4	1	4	1	2	22	133
Dorea longicatena	16	7	3	0	41	21	2	2	3	3	2	25	125
Dialister invisus	12	1	11	1	32	16	3	0	8	18	2	17	121
Faecalibacterium prausnitzii	18	2	6	1	38	18	2	0	6	0	0	24	115
Coprococcus comes	21	3	5	0	36	17	1	3	3	1	2	22	114
Coprobacter fastidiosus	10	2	9	11	3	25	7	4	2	21	0	20	114
Sutterella wadsworthensis	13	0	11	3	15	17	6	2	2	24	0	21	114
Barnesiella intestinihominis	8	3	10	11	5	24	2	4	3	18	2	16	106
Ruminococcus torques	14	2	2	0	34	16	6	0	2	2	2	21	101
Actinomyces naeslundii	13	4	8	14	14	19	6	1	3	1	3	15	101
Coprococcus eutactus	19	3	4	0	13	21	9	1	4	0	3	23	100
Eggerthella lenta	9	2	21	5	25	12	1	1	3	1	1	18	99

Actinomyces odontolyticus	15	4	8	14	10	19	8	1	0	1	1	12	93
Turicibacter sanguinis	14	4	1	1	19	16	1	6	5	0	0	20	87
Catenibacterium mitsuokai	10	2	3	0	13	16	1	1	4	1	0	18	69

Cells show the number of curated candidate protein hits per bacterial genome and functional signature.

Table S4B. Genome-by-signature presence/absence matrix

Bacterium	Butyrate	Acetate	Propionate	Vitamin K/ menaquinone	Vitamin B12/ cobalamin	Folate/ one-carbon	Tryptophan/ indole	Bile acid-related	Polyamines	LPS/ lipid A	Flagella/ TLR5	Peptido-glycan	Total curated hits
Parabacteroides distasonis	+	+	+	+	+	+	+	+	+	+	+	+	192
Roseburia intestinalis	+	+	+	-	+	+	+	+	+	-	+	+	179
Agathobacter rectalis	+	+	+	-	+	+	+	+	+	+	+	+	169
Bacteroides fragilis	+	+	+	+	+	+	+	+	+	+	+	+	163
Odoribacter splanchnicus	+	+	+	+	+	+	+	+	+	+	+	+	161
Bacteroides uniformis	+	+	+	+	+	+	+	+	+	+	+	+	160
Oscillibacter valericigenes	+	+	+	+	+	+	+	+	+	-	+	+	160
Butyricimonas virosa	+	+	+	+	+	+	+	+	+	+	+	+	148
Megamonas funiformis	+	+	+	+	+	+	+	+	+	+	-	+	140

Dialister succinatiphilus	+	+	+	+	+	+	+	+	+	+	+	+	138
Megamonas hypermegale	+	+	+	+	+	+	+	+	+	+	-	+	135
Dorea formicigenerans	+	+	+	+	+	+	+	+	+	+	+	+	133
Dorea longicatena	+	+	+	-	+	+	+	+	+	+	+	+	125
Dialister invisus	+	+	+	+	+	+	+	-	+	+	+	+	121
Faecalibacterium prausnitzii	+	+	+	+	+	+	+	-	+	-	-	+	115
Coprococcus comes	+	+	+	-	+	+	+	+	+	+	+	+	114
Coprobacter fastidiosus	+	+	+	+	+	+	+	+	+	+	-	+	114
Sutterella wadsworthensis	+	-	+	+	+	+	+	+	+	+	-	+	114
Barnesiella intestinihominis	+	+	+	+	+	+	+	+	+	+	+	+	106
Ruminococcus torques	+	+	+	-	+	+	+	-	+	+	+	+	101
Actinomyces naeslundii	+	+	+	+	+	+	+	+	+	+	+	+	101

Coprococcus eutactus	+	+	+	-	+	+	+	+	+	-	+	+	100
Eggerthella lenta	+	+	+	+	+	+	+	+	+	+	+	+	99
Actinomyces odontolyticus	+	+	+	+	+	+	+	+	-	+	+	+	93
Turicibacter sanguinis	+	+	+	+	+	+	+	+	+	-	-	+	87
Catenibacterium mitsuokai	+	+	+	-	+	+	+	+	+	+	-	+	69

A plus sign indicates at least one curated candidate hit in the corresponding genome-signature pair; a minus sign indicates no candidate hit detected under the current curation rules.
