# Supplementary material for: Genome-Resolved Functional Profiling of Osteoporosis-Associated Gut Bacteria Highlights Putative Metabolic and Immunogenic Signatures of the Gut–Bone Axis
Source: Cells. 2026 Aug 29;15(17):1573. doi: 10.3390/cells15171573 (PMC13565464; doi:10.3390/cells15171573)
Supplement: Supplementary file 1 [file cells-15-01573-s001.zip › Supplementary_Tables/Table S5.pdf]

Table S5. eggNOG annotation summary by bacterial genome.

Selected organism | assembly accession	proteins_ annotated	unique_COG_ categories	unique_KEGG_ KO	unique_KEGG_ Pathways	unique_KEGG_ Modules	unique_ EC	unique_ CAZy	unique_ PFAMs	unique_ GOs
Parabacteroides distasonis | GCF_024758965.1	4623	63	1810	430	272	885	41	2359	2673
Bacteroides fragilis | GCF_040687795.1	4599	61	1758	430	269	861	38	2398	2656
Bacteroides uniformis | GCF_018291785.1	4562	57	1733	426	262	852	47	2342	2668
Oscillibacter valericigenes | GCF_000283575.1	4058	61	1769	432	285	729	19	2139	2503
Roseburia intestinalis | GCF_900537995.1	3863	58	1782	426	279	784	33	2235	2539
Butyricimonas virosa | GCF_025148635.1	3681	57	1573	426	247	754	24	2017	2470
Odoribacter splanchnicus | GCF_000190535.1	3448	53	1664	428	257	790	29	2123	2579
Agathobacter rectalis | GCF_036419255.1	3416	63	1684	396	256	734	28	2119	2530
Dorea formicigenerans | GCF_051626395.1	3292	54	1665	392	269	727	21	1953	2353
Dorea longicatena | GCF_023008345.1	3169	53	1603	404	252	704	20	1937	2514
Allocoprococcus comes | GCF_016904155.1	3000	51	1610	394	272	696	19	1955	2510
Eggerthella lenta | GCF_021378705.1	2996	59	1611	422	261	704	13	1879	2695
Faecalibacterium prausnitzii | GCF_049532955.1	2731	53	1463	388	244	654	20	1820	2312
Actinomyces naeslundii | GCF_023170065.1	2610	57	1515	420	251	731	21	1802	3032

Mediterraneibacter torques | GCF_044591705.1	2606	50	1482	396	244	648	22	1887	2313
Coprobacter fastidiosus | GCF_030296935.1	2546	56	1525	418	240	748	44	1953	2559
Coprococcus eutactus | GCF_020735705.1	2512	47	1468	388	228	651	25	1879	2349
Turicibacter sanguinis | GCF_013046825.1	2491	53	1581	400	254	681	19	1871	2583
Barnesiella intestinihominis | GCF_046603765.1	2381	55	1421	416	229	679	41	1945	2499
Catenibacterium mitsuokai | GCF_025148785.1	2373	49	1386	404	227	637	27	1759	2235
Sutterella wadsworthensis | GCF_016724805.1	2320	52	1633	412	271	668	16	1695	3821
Megamonas funiformis | GCF_010669225.1	2306	50	1664	382	252	769	24	1859	2610
Megamonas hypermegale | GCF_963931125.1	2266	48	1648	394	259	759	25	1845	2604
Schaalia odontolytica | GCF_055396835.1	2084	54	1288	390	227	598	19	1621	2777
Dialister succinatiphilus | GCF_928721695.1	1880	47	1353	362	217	626	13	1585	2357
Dialister invisus | GCF_000160055.1	1613	47	1196	330	193	554	15	1491	2283

Counts reflect unique annotation terms recovered from the eggNOG-based output used for downstream curation. Genome labels follow the current NCBI taxonomy and assembly accessions reported in Table S1. Historical or literature-based candidate names are retained in Table S1 to document the evidence base for species selection. Annotation counts are presented as descriptive genome-level annotation coverage and should not be interpreted as normalized measures of functional enrichment across genomes.
