# Supplementary material for: Genome-Resolved Functional Profiling of Osteoporosis-Associated Gut Bacteria Highlights Putative Metabolic and Immunogenic Signatures of the Gut–Bone Axis
Source: Cells. 2026 Aug 29;15(17):1573. doi: 10.3390/cells15171573 (PMC13565464; doi:10.3390/cells15171573)
Supplement: Supplementary file 1 [file cells-15-01573-s001.zip › Supplementary_Tables/Table S6.pdf]

Table S6 A. Normalized gut-bone functional burden by bacterial genome

Bacterium	Analysis role	Annotated proteins	Total curated hits	Total hits per 1,000 proteins	Metabolic hits	Immunogenic hits	Metabolic hits per 1,000 proteins	Immunogenic hits per 1,000 proteins	Metabolic:immunogenic ratio	Functional profile
Dialister invisus	Main OP/low-BMD candidate	1613	121	75.02	84	37	52.08	22.94	2.27	Mixed high-burden profile
Dialister succinatiphilus	Main OP/low-BMD candidate	1880	138	73.40	99	39	52.66	20.74	2.54	Mixed high-burden profile
Megamonas funiformis	Normal-BMD-associated comparator	2306	140	60.71	105	35	45.53	15.18	3.00	Metabolic-enriched profile
Megamonas hypermegale	Normal-BMD-associated comparator	2266	135	59.58	101	34	44.57	15.00	2.97	Metabolic-enriched profile
Agathobacter rectalis	Normal-BMD-associated comparator	3416	169	49.47	90	79	26.35	23.13	1.14	Immunogenic-enriched profile
Sutterella wadsworthensis	Main OP/low-BMD candidate	2320	114	49.14	69	45	29.74	19.40	1.53	Immunogenic-enriched profile
Odoribacter splanchnicus	Main OP/low-BMD candidate	3448	161	46.69	108	53	31.32	15.37	2.04	Immunogenic-enriched profile
Roseburia intestinalis	Normal-BMD-associated comparator	3863	179	46.34	100	79	25.89	20.45	1.27	Immunogenic-enriched profile

Coprobacter fastidiosus	Main OP/low-BMD candidate	2546	114	44.78	73	41	28.67	16.10	1.78	Immunogenic-enriched profile
Actinomyces odontolyticus	Secondary OP candidate	2084	93	44.63	79	14	37.91	6.72	5.64	Metabolic-enriched profile
Barnesiella intestinihominis	Main OP/low-BMD candidate	2381	106	44.52	70	36	29.40	15.12	1.94	Lower/intermediate functional burden
Faecalibacterium prausnitzii	Contradictory/functional comparator	2731	115	42.11	91	24	33.32	8.79	3.79	Metabolic-enriched profile
Parabacteroides distasonis	Main OP/low-BMD candidate	4623	192	41.53	142	50	30.72	10.82	2.84	Balanced moderate-high profile
Dorea formicigenerans	Normal-BMD-associated comparator	3292	133	40.40	108	25	32.81	7.59	4.32	Metabolic-enriched profile
Butyricimonas virosa	Main OP/low-BMD candidate	3681	148	40.21	101	47	27.44	12.77	2.15	Lower/intermediate functional burden
Coprococcus eutactus	Normal-BMD-associated comparator	2512	100	39.81	74	26	29.46	10.35	2.85	Lower/intermediate functional burden
Dorea longicatena	Normal-BMD-associated comparator	3169	125	39.44	95	30	29.98	9.47	3.17	Lower/intermediate functional burden
Oscillibacter valericigenes	Main OP/low-BMD candidate	4058	160	39.43	100	60	24.64	14.79	1.67	Lower/intermediate functional burden

Ruminococcus torques	Normal-BMD-associated comparator	2606	101	38.76	76	25	29.16	9.59	3.04	Lower/intermediate functional burden
Actinomyces naeslundii	Secondary OP candidate	2610	101	38.70	82	19	31.42	7.28	4.32	Lower/intermediate functional burden
Coprococcus comes	Normal-BMD-associated comparator	3000	114	38.00	89	25	29.67	8.33	3.56	Lower/intermediate functional burden
Bacteroides fragilis	Main OP/low-BMD candidate	4599	163	35.44	119	44	25.88	9.57	2.70	Lower/intermediate functional burden
Bacteroides uniformis	Main OP/low-BMD candidate	4562	160	35.07	114	46	24.99	10.08	2.48	Lower/intermediate functional burden
Turicibacter sanguinis	Secondary OP candidate	2491	87	34.93	67	20	26.90	8.03	3.35	Lower/intermediate functional burden
Eggerthella lenta	Main OP/low-BMD candidate	2996	99	33.04	79	20	26.37	6.68	3.95	Lower/intermediate functional burden
Catenibacterium mitsuokai	Secondary OP candidate	2373	69	29.08	50	19	21.07	8.01	2.63	Lower/intermediate functional burden

Values were calculated as curated candidate protein hits divided by total eggNOG-annotated proteins and multiplied by 1,000. Metabolic signatures included SCFA-related signatures, bile acid-related functions, folate/one-carbon metabolism, polyamines, tryptophan/indole, vitamin B12/cobalamin, and vitamin K/menaquinone. Immunogenic signatures included LPS/lipid A, flagella/TLR5, and peptidoglycan/cell wall.

Table S6 B. Summary of functional profile classes by analysis role

Analysis role	Functional profile	Bacteria, n
Contradictory/functional comparator	Metabolic-enriched profile	1
Main OP/low-BMD candidate	Lower/intermediate functional burden	6
Main OP/low-BMD candidate	Immunogenic-enriched profile	3
Main OP/low-BMD candidate	Mixed high-burden profile	2
Main OP/low-BMD candidate	Balanced moderate-high profile	1
Normal-BMD-associated comparator	Lower/intermediate functional burden	4
Normal-BMD-associated comparator	Metabolic-enriched profile	3
Normal-BMD-associated comparator	Immunogenic-enriched profile	2
Secondary OP candidate	Lower/intermediate functional burden	3
Secondary OP candidate	Metabolic-enriched profile	1

Classes are descriptive comparative in silico profiles and should not be interpreted as experimental phenotypes

**Bacterium**	**Annotated proteins**	**Primary hits**	**Strict hits**	**Primary burden**	**Strict burden**	**Primary rank**	**Strict rank**	**Primary metabolic**	**Strict metabolic**	**Primary immunogenic**	**Strict immunogenic**	**Primary profile**	**Strict profile**	**Concordant**
Dialister invisus	1613	121	100	75.02	62.00	1	1	52.08	40.92	22.94	21.08	Mixed high-burden profile	Mixed high-burden profile	Yes
Dialister succinatiphilus	1880	138	112	73.40	59.57	2	2	52.66	39.89	20.74	19.68	Mixed high-burden profile	Mixed high-burden profile	Yes
Megamonas hypermegale	2266	135	105	59.58	46.34	4	3	44.57	31.77	15.00	14.56	Metabolic-enriched profile	Metabolic-enriched profile	Yes
Megamonas funiformis	2306	140	106	60.71	45.97	3	4	45.53	31.22	15.18	14.74	Metabolic-enriched profile	Metabolic-enriched profile	Yes
Odoribacter splanchnicus	3448	161	136	46.69	39.44	7	5	31.32	24.65	15.37	14.79	Immunogenic-enriched profile	Mixed high-burden profile	**No**
Agathobacter rectalis	3416	169	133	49.47	38.93	5	6	26.35	18.44	23.13	20.49	Immunogenic-enriched profile	Immunogenic-enriched profile	Yes
Sutterella wadsworthensis	2320	114	85	49.14	36.64	6	7	29.74	18.10	19.40	18.53	Immunogenic-enriched profile	Immunogenic-enriched profile	Yes
Coprobacter fastidiosus	2546	114	91	44.78	35.74	9	8	28.67	19.64	16.10	16.10	Immunogenic-enriched profile	Immunogenic-enriched profile	Yes

Table S6C. Sensitivity analysis comparing primary candidate-set and strict high-confidence genome-level functional burdens and classifications.

Barnesiella intestinihominis	2381	106	84	44.52	35.28	11	9	29.40	21.00	15.12	14.28	Lower/intermediate functional burden	Balanced moderate-high profile	**No**
Roseburia intestinalis	3863	179	131	46.34	33.91	8	10	25.89	15.53	20.45	18.38	Immunogenic-enriched profile	Immunogenic-enriched profile	Yes
Butyricimonas virosa	3681	148	122	40.21	33.14	15	11	27.44	20.92	12.77	12.22	Lower/intermediate functional burden	Balanced moderate-high profile	**No**
Actinomyces odontolyticus	2084	93	64	44.63	30.71	10	12	37.91	24.95	6.72	5.76	Metabolic-enriched profile	Metabolic-enriched profile	Yes
Parabacteroides distasonis	4623	192	139	41.53	30.07	13	13	30.72	19.47	10.82	10.60	Balanced moderate-high profile	Balanced moderate-high profile	Yes
Faecalibacterium prausnitzii	2731	115	81	42.11	29.66	12	14	33.32	20.87	8.79	8.79	Metabolic-enriched profile	Lower/intermediate functional burden	**No**
Dorea formicigenerans	3292	133	96	40.40	29.16	14	15	32.81	21.57	7.59	7.59	Metabolic-enriched profile	Metabolic-enriched profile	Yes
Ruminococcus torques	2606	101	75	38.76	28.78	19	16	29.16	19.19	9.59	9.59	Lower/intermediate functional burden	Lower/intermediate functional burden	Yes
Coprococcus eutactus	2512	100	72	39.81	28.66	16	17	29.46	18.71	10.35	9.95	Lower/intermediate functional burden	Lower/intermediate functional burden	Yes
Coprococcus comes	3000	114	81	38.00	27.00	21	18	29.67	18.67	8.33	8.33	Lower/intermediate functional burden	Lower/intermediate functional burden	Yes
Bacteroides fragilis	4599	163	122	35.44	26.53	22	19	25.88	17.18	9.57	9.35	Lower/intermediate functional burden	Lower/intermediate functional burden	Yes

Bacteroides uniformis	4562	160	120	35.07	26.30	23	20	24.99	16.88	10.08	9.43	Lower/intermediate functional burden	Lower/intermediate functional burden	Yes
Dorea longicatena	3169	125	83	39.44	26.19	17	21	29.98	16.72	9.47	9.47	Lower/intermediate functional burden	Lower/intermediate functional burden	Yes
Oscillibacter valericigenes	4058	160	106	39.43	26.12	18	22	24.64	13.31	14.79	12.81	Lower/intermediate functional burden	Lower/intermediate functional burden	Yes
Actinomyces naeslundii	2610	101	68	38.70	26.05	20	23	31.42	20.69	7.28	5.36	Lower/intermediate functional burden	Lower/intermediate functional burden	Yes
Eggerthella lenta	2996	99	77	33.04	25.70	25	24	26.37	19.36	6.68	6.34	Lower/intermediate functional burden	Lower/intermediate functional burden	Yes
Turicibacter sanguinis	2491	87	62	34.93	24.89	24	25	26.90	16.86	8.03	8.03	Lower/intermediate functional burden	Lower/intermediate functional burden	Yes
Catenibacterium mitsuokai	2373	69	46	29.08	19.38	26	26	21.07	11.80	8.01	7.59	Lower/intermediate functional burden	Lower/intermediate functional burden	Yes

Note. Primary burden corresponds to the 3,337-hit candidate set; strict burden corresponds to the 2,497-hit high-confidence subset. Burdens are expressed as hits per 1,000 annotated proteins. Functional profiles were reassigned using the same metabolic-immunogenic classification framework, with median and third-quartile thresholds recalculated within the strict subset. Ranking concordance between primary and strict normalized burdens was high (Spearman $\rho = 0.958$, $p < 0.001$), and 22/26 genomes (84.6%) retained the same functional-profile classification. The four highest-ranked genomes were preserved in both analyses, with only the relative order of the two Megamonas species changing. **Abbreviations:** burden, normalized candidate protein hits per 1,000 annotated proteins; primary, primary candidate-hit universe; strict, strict high-confidence subset.
