# Supplementary material for: Genome-Resolved Functional Profiling of Osteoporosis-Associated Gut Bacteria Highlights Putative Metabolic and Immunogenic Signatures of the Gut–Bone Axis
Source: Cells. 2026 Aug 29;15(17):1573. doi: 10.3390/cells15171573 (PMC13565464; doi:10.3390/cells15171573)
Supplement: Supplementary file 1 [file cells-15-01573-s001.zip › Supplementary_Tables/Table S7.pdf]

Table S7 A. CAZyme repertoire by bacterial genome

Bacterium	Analysis role	Annotated proteins	Total CAZy hits	Unique CAZy families	GH hits	GT hits	CE hits	CBM hits	PL hits	AA hits	Functional profile
Barnesiella intestinihominis	Main OP/low-BMD candidate	2381	114	41	84	26	2	1	1	0	Lower/intermediate functional burden
Coprobacter fastidiosus	Main OP/low-BMD candidate	2546	112	44	69	36	1	4	2	0	Immunogenic-enriched profile
Bacteroides uniformis	Main OP/low-BMD candidate	4562	183	47	122	52	5	3	1	0	Lower/intermediate functional burden
Roseburia intestinalis	Normal-BMD-associated comparator	3863	145	33	87	50	2	6	0	0	Immunogenic-enriched profile
Catenibacterium mitsuokai	Secondary OP candidate	2373	78	27	37	33	0	5	2	1	Lower/intermediate functional burden
Coprococcus eutactus	Normal-BMD-associated comparator	2512	79	25	46	26	0	5	2	0	Lower/intermediate functional burden
Bacteroides fragilis	Main OP/low-BMD candidate	4599	143	38	90	48	1	3	1	0	Lower/intermediate functional burden
Parabacteroides distasonis	Main OP/low-BMD candidate	4623	131	41	72	55	2	2	0	0	Balanced moderate-high profile

Turicibacter sanguinis	Secondary OP candidate	2491	69	19	36	28	0	5	0	0	Lower/intermediate functional burden
Megamonas hypermegale	Normal-BMD-associated comparator	2266	59	25	22	34	0	3	0	0	Metabolic-enriched profile
Actinomyces naeslundii	Secondary OP candidate	2610	64	21	33	24	0	7	0	0	Lower/intermediate functional burden
Agathobacter rectalis	Normal-BMD-associated comparator	3416	83	28	49	29	0	5	0	0	Immunogenic-enriched profile
Ruminococcus torques	Normal-BMD-associated comparator	2606	62	22	33	27	0	2	0	0	Lower/intermediate functional burden
Actinomyces odontolyticus	Secondary OP candidate	2084	49	19	21	23	0	5	0	0	Metabolic-enriched profile
Faecalibacterium prausnitzii	Contradictory/functional comparator	2731	60	20	26	25	0	5	4	0	Metabolic-enriched profile
Megamonas funiformis	Normal-BMD-associated comparator	2306	50	24	22	25	0	3	0	0	Metabolic-enriched profile
Coprococcus comes	Normal-BMD-associated comparator	3000	63	19	32	27	0	4	0	0	Lower/intermediate functional burden
Dorea longicatena	Normal-BMD-associated comparator	3169	57	20	26	28	0	3	0	0	Lower/intermediate functional burden

Odoribacter splanchnicus	Main OP/low-BMD candidate	3448	59	29	33	24	1	1	0	0	Immunogenic-enriched profile
Dorea formicigenerans	Normal-BMD-associated comparator	3292	52	21	20	29	0	3	0	0	Metabolic-enriched profile
Dialister invisus	Main OP/low-BMD candidate	1613	23	15	5	16	1	0	1	0	Mixed high-burden profile
Butyricimonas virosa	Main OP/low-BMD candidate	3681	49	24	19	27	2	1	0	0	Lower/intermediate functional burden
Sutterella wadsworthensis	Main OP/low-BMD candidate	2320	28	16	9	18	1	0	0	0	Immunogenic-enriched profile
Dialister succinatiphilus	Main OP/low-BMD candidate	1880	20	13	2	16	2	0	0	0	Mixed high-burden profile
Oscillibacter valericigenes	Main OP/low-BMD candidate	4058	39	19	18	16	0	5	0	0	Lower/intermediate functional burden
Eggerthella lenta	Main OP/low-BMD candidate	2996	25	13	5	18	0	2	0	0	Lower/intermediate functional burden

Abbreviations: CAZy, Carbohydrate-Active enZYmes database; CAZy hits, number of annotated carbohydrate-active enzyme hits; unique CAZy families, number of distinct CAZy families detected per genome; GH, glycoside hydrolases; GT, glycosyltransferases; CE, carbohydrate esterases; CBM, carbohydrate-binding modules; PL, polysaccharide lyases; AA, auxiliary activities.

**Table S7 B.** Normalized CAZyme class matrix								
**Bacterium**	**Analysis role**	**Annotated proteins**	**GH**	**GT**	**CE**	**CBM**	**PL**	**AA**
Actinomyces naeslundii	Secondary OP candidate	2610	12.64	9.20	0.00	2.68	0.00	0.00
Actinomyces odontolyticus	Secondary OP candidate	2084	10.08	11.04	0.00	2.40	0.00	0.00
Agathobacter rectalis	Normal-BMD-associated comparator	3416	14.34	8.49	0.00	1.46	0.00	0.00
Bacteroides fragilis	Main OP/low-BMD candidate	4599	19.57	10.44	0.22	0.65	0.22	0.00
Bacteroides uniformis	Main OP/low-BMD candidate	4562	26.74	11.40	1.10	0.66	0.22	0.00
Barnesiella intestinihominis	Main OP/low-BMD candidate	2381	35.28	10.92	0.84	0.42	0.42	0.00
Butyricimonas virosa	Main OP/low-BMD candidate	3681	5.16	7.33	0.54	0.27	0.00	0.00
Catenibacterium mitsuokai	Secondary OP candidate	2373	15.59	13.91	0.00	2.11	0.84	0.42
Coprobacter fastidiosus	Main OP/low-BMD candidate	2546	27.10	14.14	0.39	1.57	0.79	0.00

Coprococcus comes	Normal-BMD-associated comparator	3000	10.67	9.00	0.00	1.33	0.00	0.00
Coprococcus eutactus	Normal-BMD-associated comparator	2512	18.31	10.35	0.00	1.99	0.80	0.00
Dialister invisus	Main OP/low-BMD candidate	1613	3.10	9.92	0.62	0.00	0.62	0.00
Dialister succinatiphilus	Main OP/low-BMD candidate	1880	1.06	8.51	1.06	0.00	0.00	0.00
Dorea formicigenerans	Normal-BMD-associated comparator	3292	6.08	8.81	0.00	0.91	0.00	0.00
Dorea longicatena	Normal-BMD-associated comparator	3169	8.20	8.84	0.00	0.95	0.00	0.00
Eggerthella lenta	Main OP/low-BMD candidate	2996	1.67	6.01	0.00	0.67	0.00	0.00
Faecalibacterium prausnitzii	Contradictory/functional comparator	2731	9.52	9.15	0.00	1.83	1.46	0.00
Megamonas funiformis	Normal-BMD-associated comparator	2306	9.54	10.84	0.00	1.30	0.00	0.00
Megamonas hypermegale	Normal-BMD-associated comparator	2266	9.71	15.00	0.00	1.32	0.00	0.00

Odoribacter splanchnicus	Main OP/low-BMD candidate	3448	9.57	6.96	0.29	0.29	0.00	0.00
Oscillibacter valericigenes	Main OP/low-BMD candidate	4058	4.44	3.94	0.00	1.23	0.00	0.00
Parabacteroides distasonis	Main OP/low-BMD candidate	4623	15.57	11.90	0.43	0.43	0.00	0.00
Roseburia intestinalis	Normal-BMD-associated comparator	3863	22.52	12.94	0.52	1.55	0.00	0.00
Ruminococcus torques	Normal-BMD-associated comparator	2606	12.66	10.36	0.00	0.77	0.00	0.00
Sutterella wadsworthensis	Main OP/low-BMD candidate	2320	3.88	7.76	0.43	0.00	0.00	0.00
Turicibacter sanguinis	Secondary OP candidate	2491	14.45	11.24	0.00	2.01	0.00	0.00

Abbreviations: GH, glycoside hydrolases; GT, glycosyltransferases; CE, carbohydrate esterases; CBM, carbohydrate-binding modules; PL, polysaccharide lyases; AA, auxiliary activities.
