# Supplementary material for: Genome-Resolved Functional Profiling of Osteoporosis-Associated Gut Bacteria Highlights Putative Metabolic and Immunogenic Signatures of the Gut–Bone Axis
Source: Cells. 2026 Aug 29;15(17):1573. doi: 10.3390/cells15171573 (PMC13565464; doi:10.3390/cells15171573)
Supplement: Supplementary file 1 [file cells-15-01573-s001.zip › Supplementary_Tables/Table S8.pdf]

Table S8 A. Global summary of selected KEGG bone-relevant pathways

kegg_pathway	pathway_name	total_raw_hits	genomes_present	mean_hits_per_1000	median_hits_per_1000	max_hits_per_1000
ko01230	Biosynthesis of amino acids	3058	26	41.05	41.81	58.69
ko02010	ABC transporters	2370	26	33.01	34.35	68.2
ko01200	Carbon metabolism	2276	26	30.14	29.41	42.37
ko02020	Two-component system	1771	26	22.79	18.51	73.28
ko02024	Quorum sensing	1380	26	19.04	19.54	32.24
ko00520	Amino sugar/nucleotide sugar metabolism	1315	26	16.84	17.27	29.92
ko00010	Glycolysis/gluconeogenesis	977	26	13.17	12.72	25.28
ko00620	Pyruvate metabolism	915	26	12.2	11.9	25.43
ko00270	Cysteine and methionine metabolism	816	26	11.01	10.74	16.49
ko00052	Galactose metabolism	815	26	10.67	10.72	18.87
ko00260	Glycine, serine and threonine metabolism	757	26	10.27	10.64	15.96
ko00051	Fructose and mannose metabolism	708	26	9.6	8.05	25.15
ko00550	Peptidoglycan biosynthesis	664	26	9.01	8.76	13.44
ko00030	Pentose phosphate pathway	633	26	8.53	8.42	16.77
ko00860	Porphyrin metabolism	594	26	8.35	7.39	25.42
ko03070	Bacterial secretion system	578	26	7.69	6.99	14.74
ko00650	Butanoate metabolism	562	26	7.41	6.13	18.97
ko00640	Propanoate metabolism	555	26	7.24	6.75	12.57
ko00020	Citrate cycle/TCA cycle	534	26	7.07	6.29	19.83

ko00790	Folate biosynthesis	523	26	7.17	6.53	11.21
ko00061	Fatty acid biosynthesis	383	26	5.04	4.88	8.19
ko00040	Pentose/glucuronate interconversions	383	26	4.97	4.49	13.68
ko00900	Terpenoid backbone biosynthesis	381	26	5.16	5.46	6.82
ko00280	Valine, leucine and isoleucine degradation	281	26	3.64	3.56	7.07
ko02060	Phosphotransferase system	273	23	4.14	1.44	23.18
ko00130	Ubiquinone/terpenoid-quinone biosynthesis	189	25	2.48	2.4	5.28
ko00071	Fatty acid degradation	180	26	2.39	2.32	4.44
ko02040	Flagellar assembly	155	24	1.71	0.51	12.3
ko00380	Tryptophan metabolism	63	25	0.84	0.77	3.0
ko00121	Secondary bile acid biosynthesis	36	15	0.43	0.39	1.61
ko00120	Primary bile acid biosynthesis	31	13	0.37	0.11	1.61

Table S8 B. Normalized selected KEGG pathway burden by genome

species	analysis_role	proteins_annotated	total_selected_kegg_hits	normalized_selected_kegg_burden
Megamonas hypermegale	Normal-BMD-associated comparator	2266	1006	443.95
Sutterella wadsworthensis	Main OP/low-BMD candidate	2320	1009	434.91
Megamonas funiformis	Normal-BMD-associated comparator	2306	1001	434.08
Dialister succinatiphilus	Main OP/low-BMD candidate	1880	710	377.66
Dialister invisus	Main OP/low-BMD candidate	1613	608	376.94
Catenibacterium mitsuokai	Secondary OP candidate	2373	849	357.77
Turicibacter sanguinis	Secondary OP candidate	2491	860	345.24
Eggerthella lenta	Main OP/low-BMD candidate	2996	1019	340.12
Actinomyces naeslundii	Secondary OP candidate	2610	875	335.25

Dorea formicigenerans	Normal-BMD-associated comparator	3292	1074	326.25
Oscillibacter valericigenes	Main OP/low-BMD candidate	4058	1320	325.28
Coprococcus comes	Normal-BMD-associated comparator	3000	974	324.67
Ruminococcus torques	Normal-BMD-associated comparator	2606	840	322.33
Actinomyces odontolyticus	Secondary OP candidate	2084	671	321.98
Coprococcus eutactus	Normal-BMD-associated comparator	2512	793	315.68
Coprobacter fastidiosus	Main OP/low-BMD candidate	2546	792	311.08
Faecalibacterium prausnitzii	Contradictory/functional comparator	2731	846	309.78
Roseburia intestinalis	Normal-BMD-associated comparator	3863	1180	305.46
Barnesiella intestinihominis	Main OP/low-BMD candidate	2381	709	297.77

Dorea longicatena	Normal-BMD-associated comparator	3169	927	292.52
Agathobacter rectalis	Normal-BMD-associated comparator	3416	982	287.47
Odoribacter splanchnicus	Main OP/low-BMD candidate	3448	908	263.34
Parabacteroides distasonis	Main OP/low-BMD candidate	4623	1176	254.38
Bacteroides uniformis	Main OP/low-BMD candidate	4562	1100	241.12
Bacteroides fragilis	Main OP/low-BMD candidate	4599	1084	235.7
Butyricimonas virosa	Main OP/low-BMD candidate	3681	843	229.01

Abbreviations: KEGG, Kyoto Encyclopedia of Genes and Genomes; OP, osteoporosis; BMD, bone mineral density.
