# Supplementary material for: Genome-Resolved Functional Profiling of Osteoporosis-Associated Gut Bacteria Highlights Putative Metabolic and Immunogenic Signatures of the Gut–Bone Axis
Source: Cells. 2026 Aug 29;15(17):1573. doi: 10.3390/cells15171573 (PMC13565464; doi:10.3390/cells15171573)
Supplement: Supplementary file 1 [file cells-15-01573-s001.zip › Supplementary_Tables/Table S9.pdf]

Table S9. gutSMASH-predicted metabolic gene clusters/protoclusters by bacterial genome.

Bacterium	Analysis role	Genome accession	Record ID	Protocluster number	gutSMASH product	Product category	Location	CDS count	Definition domains
Actinomyces naeslundii	Secondary OP candidate	GCF_023170065.1	NZ_AP025697.1	1	Fumarate2succinate	Fermentation / energy metabolism	[246321:269436]	11	FAD_binding_2; Fer2_3; SucD_FumR; Succ_DH_flav_C
Actinomyces naeslundii	Secondary OP candidate	GCF_023170065.1	NZ_AP025697.1	8	Fumarate2succinate	Fermentation / energy metabolism	[2047314:2070804]	9	FAD_binding_2; Fer2_3; Fer4_7; SucD_FumR; Succ_DH_flav_C
Actinomyces naeslundii	Secondary OP candidate	GCF_023170065.1	NZ_AP025697.1	2	TPP_AA_metabolism	Amino-acid metabolism	[483675:507019]	6	ALS_ss_C; IlvC; TPP_enzyme_C
Actinomyces naeslundii	Secondary OP candidate	GCF_023170065.1	NZ_AP025697.1	3	Arginine2_Hcarbonate	Amino-acid metabolism	[1110698:1133997]	8	AA_kinase; Amidinotransf; OTCace; OTCace_N
Actinomyces naeslundii	Secondary OP candidate	GCF_023170065.1	NZ_AP025697.1	4	Pyruvate2acetate-formate	Fermentation / energy	[1215468:1238804]	7	PFL_acetate2; Radical_SAM

						metabolism			
Actinomyces naeslundii	Secondary OP candidate	GCF_023170065.1	NZ_AP025697.1	5	Putrescine2spermidine	Polyamine metabolism	[1296880:1319830]	10	AdoMet_dc; Spermine_synth
Actinomyces naeslundii	Secondary OP candidate	GCF_023170065.1	NZ_AP025697.1	6	Nitrate_reductase	Redox / nitrogen metabolism	[1822559:1849586]	10	Fer4_11; Molybdopterin; Molydop_binding; Nitr_red_bet_C; Nitrate_red_del; Nitrate_red_gam
Actinomyces naeslundii	Secondary OP candidate	GCF_023170065.1	NZ_AP025697.1	7	NADH_dehydrogenase_I	Fermentation / energy metabolism	[1991066:2026975]	22	Complex1_30kDa; Complex1_49kDa; Fe-2S_thioredx; Fer2_4; Fer4_7; NADH-G_4Fe-4S_3; NADH_4Fe-4S; NADHdh; Proton_antipo_M; Proton_antipo_N; SLBB
Actinomyces naeslundii	Secondary OP candidate	GCF_023170065.1	NZ_AP025697.1	9	Others_HGD_unassigned	Aromatic / phenolic	[2445107:2470384]	3	BcrAD_BadFG; HGD-D

						metabolism			
Actinomyces odontolyticus	Secondary OP candidate	GCF_055396835.1	NZ_AP041216.1	1	Nitrate_reductase	Redox / nitrogen metabolism	[258476:285265]	9	Fer4_11; Molybdopterin; Molydop_binding; Nitr_red_bet_C; Nitrate_red_del; Nitrate_red_gam
Actinomyces odontolyticus	Secondary OP candidate	GCF_055396835.1	NZ_AP041216.1	2	Others_HGD_unassigned	Aromatic / phenolic metabolism	[279571:304146]	5	BcrAD_BadFG; HGD-D
Actinomyces odontolyticus	Secondary OP candidate	GCF_055396835.1	NZ_AP041216.1	3	NADH_dehydrogenase_I	Fermentation / energy metabolism	[517112:552768]	21	Complex1_30kDa; Complex1_49kDa; Fe-2S_thioredx; Fer2_4; Fer4_7; NADH-G_4Fe-4S_3; NADH_4Fe-4S; NADHdh; Oxidored_q3; Oxidored_q6; Proton_antipo_M;

								Proton_antipo_N; SLBB
Actinomyces odontolyticus	Secondary OP candidate	GCF_055396835.1	NZ_AP041216.1	4	TPP_AA_metabolism	Amino-acid metabolism	[993352:1016659]	3	ALS_ss_C; IlvC; TPP_enzyme_C
Actinomyces odontolyticus	Secondary OP candidate	GCF_055396835.1	NZ_AP041216.1	5	Respiratory_glycerol	Other / unassigned	[1139029:1163260]	8	DAO; FAD_binding_2; Fer2_BFD; Fer4_8
Actinomyces odontolyticus	Secondary OP candidate	GCF_055396835.1	NZ_AP041216.1	6	Pyruvate2acetate-formate	Fermentation / energy metabolism	[1661555:1685051]	8	PFL_acetate2; Radical_SAM
Actinomyces odontolyticus	Secondary OP candidate	GCF_055396835.1	NZ_AP041216.1	7	Fumarate2succinate	Fermentation / energy metabolism	[2140980:2164430]	8	FAD_binding_2; Fer2_3; SucD_FumR; Succ_DH_flav_C
Agathobacter rectalis	Normal-BMD-associated comparator	GCF_036419255.1	NZ_CP143947.1	1	acetate2butyrate	Fermentation / energy metabolism	[625976:651448]	8	Acyl-CoA_dh_1; ETF; ETF_alpha; HCDH; HCDH_N; Thiolase_C; Thiolase_N

Agathobacter rectalis	Normal-BMD-associated comparator	GCF_036419255.1	NZ_CP143947.1	2	Others_HGD_unassigned	Aromatic / phenolic metabolism	[934741:956879]	6	BcrAD_BadFG; HGD-D
Agathobacter rectalis	Normal-BMD-associated comparator	GCF_036419255.1	NZ_CP143947.1	3	Others_HGD_unassigned	Aromatic / phenolic metabolism	[1439968:1464219]	9	BcrAD_BadFG; HGD-D
Agathobacter rectalis	Normal-BMD-associated comparator	GCF_036419255.1	NZ_CP143947.1	4	succinate2propionate	Fermentation / energy metabolism	[1468999:1494267]	13	Biotin_lipoyl; CT_Vparvula; Complex1_51K; Fer4; Fer4_9; HMGL-like; NQR2_RnfD_RnfE; OAD_beta; OAD_gamma; PYC_OADA; RnfC_N; SLBB
Agathobacter rectalis	Normal-BMD-associated comparator	GCF_036419255.1	NZ_CP143947.1	5	Rnf_complex	Fermentation / energy metabolism	[1481589:1506631]	14	Complex1_51K; FMN_bind; FeS; Fer4; Fer4_9; HMGL-like; NQR2_RnfD_RnfE; OAD_beta;

									PYC_OADA; Rnf-Nqr; RnfC_N; SLBB
Agathobacter rectalis	Normal-BMD-associated comparator	GCF_036419255.1	NZ_CP143947.1	6	Adenylylsulfate_reductase	Sulfur metabolism	[2626910:2648915]	11	FAD_binding_2; Fer4_9
Agathobacter rectalis	Normal-BMD-associated comparator	GCF_036419255.1	NZ_CP143947.1	7	xanthine_dehydrogenase	Redox / nitrogen metabolism	[3265321:3289092]	10	Ald_Xan_dh_C; FAD_binding_5; Fer2; Fer2_2; MoCoBD_1; MoCoBD_2
Agathobacter rectalis	Normal-BMD-associated comparator	GCF_036419255.1	NZ_CP143947.1	8	Putrescine2spermidine	Polyamine metabolism	[3420802:3444171]	7	Orn_DAP_Arg_deC; Sacchrp_dh_C; Spermine_synt_N; Spermine_synth
Bacteroides fragilis	Main OP/low-BMD candidate	GCF_040687795.1	NZ_CP131534.1	1	Rnf_complex	Fermentation / energy metabolism	[678813:703914]	10	Complex1_51K; FMN_bind; FeS; Fer4; NQR2_RnfD_RnfE; Rnf-Nqr; RnfC_N; SLBB
Bacteroides fragilis	Main OP/low-BMD candidate	GCF_040687795.1	NZ_CP131534.1	2	succinate2propionate	Fermentation / energy metabolism	[1318867:1343506]	12	Biotin_lipoyl; CT_Btheta; Glyoxalase_4; OAD_beta; OAD_gamma

Bacteroides fragilis	Main OP/low-BMD candidate	GCF_040687795.1	NZ_CP131534.1	3	PFOR_II_pathway	Fermentation / energy metabolism	[1384156:1407711]	7	EKR; Fer4_16; PFOR_II; POR; POR_N; TPP_enzyme_C
Bacteroides fragilis	Main OP/low-BMD candidate	GCF_040687795.1	NZ_CP131534.1	4	TPP_AA_metabolism	Amino-acid metabolism	[1951197:1977798]	4	ALS_ss_C; ILVD_EDD; IlvC; TPP_enzyme_C
Bacteroides fragilis	Main OP/low-BMD candidate	GCF_040687795.1	NZ_CP131534.1	5	putative_2-oxoglutarate_ferredoxin_oxidoreductase	Redox / nitrogen metabolism	[2899699:2922564]	2	PFOR_II; POR; POR_N; TPP_enzyme_C
Bacteroides fragilis	Main OP/low-BMD candidate	GCF_040687795.1	NZ_CP131534.1	6	Fumarate2succinate	Fermentation / energy metabolism	[3059868:3083327]	3	FAD_binding_2; Fer2_3; Fer4_7; SucD_FumR; Succ_DH_flav_C
Bacteroides fragilis	Main OP/low-BMD candidate	GCF_040687795.1	NZ_CP131534.1	7	NADH_dehydrogenase_I	Fermentation / energy metabolism	[4137005:4166942]	13	Complex1_30kDa; Complex1_49kDa; Fer4_7; NADHdh; Oxidored_q2; Oxidored_q3; Oxidored_q4; Oxidored_q6;

									Proton_antipo_M; Proton_antipo_N
Bacteroides fragilis	Main OP/low-BMD candidate	GCF_040687795.1	NZ_CP131534.1	8	TPP_fatty_acids	Lipid / fatty-acid metabolism	[4825591:4851467]	8	ECH_1; HBT; TPP_enzyme_C
Bacteroides fragilis	Main OP/low-BMD candidate	GCF_040687795.1	NZ_CP131534.1	9	Pyruvate2acetate-formate	Fermentation / energy metabolism	[4854158:4877117]	6	PFL_acetate1; Radical_SAM
Bacteroides fragilis	Main OP/low-BMD candidate	GCF_040687795.1	NZ_CP131534.1	10	porA	Fermentation / energy metabolism	[5129940:5152865]	10	Fer4_7; PFOR_II; POR; POR_N; TPP_enzyme_C
Bacteroides fragilis	Main OP/low-BMD candidate	GCF_040687795.1	NZ_CP131534.1	11	Sulfate2PAPS	Sulfur metabolism	[5152253:5175243]	4	APS_kinase; GTP_EFTU; PAPS_reduct
Bacteroides fragilis	Main OP/low-BMD candidate	GCF_040687795.1	NZ_CP131534.1	12	Phenylpyruvate_ferredoxin_oxidoreductase	Fermentation / energy metabolism	[5181656:5203837]	4	POR; POR_N; TPP_enzyme_C

Bacteroides uniformis	Main OP/low-BMD candidate	GCF_018291785.1	NZ_CP072239.1	1	TPP_AA_metabolism	Amino-acid metabolism	[209646:235728]	7	ALS_ss_C; ILVD_EDD; IlvC; TPP_enzyme_C
Bacteroides uniformis	Main OP/low-BMD candidate	GCF_018291785.1	NZ_CP072239.1	2	putative_2-oxoglutarate_ferredoxin_oxidoreductase	Redox / nitrogen metabolism	[1136611:1159551]	4	PFOR_II; POR; POR_N; TPP_enzyme_C
Bacteroides uniformis	Main OP/low-BMD candidate	GCF_018291785.1	NZ_CP072239.1	3	Fumarate2succinate	Fermentation / energy metabolism	[1253499:1276970]	6	FAD_binding_2; Fer2_3; Fer4_7; SucD_FumR; Succ_DH_flav_C
Bacteroides uniformis	Main OP/low-BMD candidate	GCF_018291785.1	NZ_CP072239.1	4	NADH_dehydrogenase_I	Fermentation / energy metabolism	[2292521:2322746]	11	Complex1_30kDa; Complex1_49kDa; Fer4_7; NADHdh; Oxidored_q2; Oxidored_q3; Oxidored_q4; Oxidored_q6; Proton_antipo_M; Proton_antipo_N
Bacteroides uniformis	Main OP/low-BMD candidate	GCF_018291785.1	NZ_CP072239.1	5	PFOR_II_pathway	Fermentation / energy	[2738269:2761827]	4	EKR; Fer4_16; PFOR_II; POR;

						metabolism			POR_N; TPP_enzyme_C
Bacteroides uniformis	Main OP/low-BMD candidate	GCF_018291785.1	NZ_CP072239.1	6	succinate2propionate	Fermentation / energy metabolism	[3041503:3066212]	8	Biotin_lipoyl; CT_Btheta; Glyoxalase_4; OAD_beta; OAD_gamma
Bacteroides uniformis	Main OP/low-BMD candidate	GCF_018291785.1	NZ_CP072239.1	7	porA	Fermentation / energy metabolism	[3140523:3163367]	8	Fer4_7; PFOR_II; POR; POR_N; TPP_enzyme_C
Bacteroides uniformis	Main OP/low-BMD candidate	GCF_018291785.1	NZ_CP072239.1	8	Sulfate2PAPS	Sulfur metabolism	[3167192:3190606]	6	APS_kinase; GTP_EFTU; PAPS_reduct
Bacteroides uniformis	Main OP/low-BMD candidate	GCF_018291785.1	NZ_CP072239.1	9	Phenylpyruvate_ferredoxin_oxidoreductase	Fermentation / energy metabolism	[3190015:3212194]	5	POR; POR_N; TPP_enzyme_C
Bacteroides uniformis	Main OP/low-BMD candidate	GCF_018291785.1	NZ_CP072239.1	10	Rnf_complex	Fermentation / energy metabolism	[3857140:3882375]	8	Complex1_51K; FMN_bind; FeS; Fer4; NQR2_RnfD_RnfE; Rnf-Nqr; RnfC_N; SLBB

Bacteroides uniformis	Main OP/low-BMD candidate	GCF_018291785.1	NZ_CP072239.1	11	Pyruvate2acetate-formate	Fermentation / energy metabolism	[4626848:4649804]	4	PFL_acetate1; Radical_SAM
Barnesiella intestinihominis	Main OP/low-BMD candidate	GCF_046603765.1	NZ_CP176624.1	1	Fumarate2succinate	Fermentation / energy metabolism	[917840:941388]	6	FAD_binding_2; Fer2_3; SucD_FumR; Succ_DH_flav_C
Barnesiella intestinihominis	Main OP/low-BMD candidate	GCF_046603765.1	NZ_CP176624.1	2	Others_HGD_unassigned	Aromatic / phenolic metabolism	[1340580:1364777]	1	BcrAD_BadFG; HGD-D
Barnesiella intestinihominis	Main OP/low-BMD candidate	GCF_046603765.1	NZ_CP176624.1	3	PFOR_II_pathway	Fermentation / energy metabolism	[1691069:1714633]	5	EKR; Fer4_16; PFOR_II; POR; POR_N; TPP_enzyme_C
Barnesiella intestinihominis	Main OP/low-BMD candidate	GCF_046603765.1	NZ_CP176624.1	4	succinate2propionate	Fermentation / energy metabolism	[1991963:2016490]	8	Biotin_lipoyl; CT_Btheta; Glyoxalase_4; OAD_beta; OAD_gamma

Barnesiella intestinihominis	Main OP/low-BMD candidate	GCF_046603765.1	NZ_CP176624.1	5	Rnf_complex	Fermentation / energy metabolism	[2686908:2712307]	14	Complex1_51K; FMN_bind; FeS; Fer4; Fer4_7; Fer4_9; NQR2_RnfD_RnfE; PFOR_II; POR; POR_N; Rnf-Nqr; RnfC_N; SLBB; TPP_enzyme_C
Barnesiella intestinihominis	Main OP/low-BMD candidate	GCF_046603765.1	NZ_CP176624.1	6	porA	Fermentation / energy metabolism	[2692079:2714772]	13	Complex1_51K; FMN_bind; FeS; Fer4; Fer4_7; Fer4_9; NQR2_RnfD_RnfE; PFOR_II; POR; POR_N; Rnf-Nqr; RnfC_N; SLBB; TPP_enzyme_C
Barnesiella intestinihominis	Main OP/low-BMD candidate	GCF_046603765.1	NZ_CP176624.1	7	Glycine_cleavage	Fermentation / energy metabolism	[2719061:2743376]	9	Aminotran_5; GCV_H; GCV_T; GCV_T_C; GDC-P
Barnesiella intestinihominis	Main OP/low-BMD candidate	GCF_046603765.1	NZ_CP176624.1	8	TPP_AA_metabolism	Amino-acid	[3213035:3238997]	7	ALS_ss_C; ILVD_EDD; IlvC; TPP_enzyme_C

						metabolism			
Butyricimonas virosa	Main OP/low-BMD candidate	GCF_025148635.1	NZ_CP102269.1	1	Rnf_complex	Fermentation / energy metabolism	[235582:260851]	11	Complex1_51K; FMN_bind; FeS; Fer4; Fer4_9; NQR2_RnfD_RnfE; Rnf-Nqr; RnfC_N; SLBB
Butyricimonas virosa	Main OP/low-BMD candidate	GCF_025148635.1	NZ_CP102269.1	2	Phenylpyruvate_ferredoxin_oxidoreductase	Fermentation / energy metabolism	[262264:284492]	2	POR; POR_N; TPP_enzyme_C
Butyricimonas virosa	Main OP/low-BMD candidate	GCF_025148635.1	NZ_CP102269.1	3	porA	Fermentation / energy metabolism	[519489:542221]	5	Fer4_7; PFOR_II; POR; POR_N; TPP_enzyme_C
Butyricimonas virosa	Main OP/low-BMD candidate	GCF_025148635.1	NZ_CP102269.1	4	Lysine_degradation	Amino-acid metabolism	[765430:796866]	15	AcetylCoA_hyd_C; Acyl-CoA_dh_1; B12-binding; BKACE; CoA_trans; HBT; LAM_C; Lys-AminoMut_A; OAM_dimer;

Butyricimonas virosa	Main OP/low-BMD candidate	GCF_025148635.1	NZ_CP102269.1	5	fatty_acids-unassigned	Lipid / fatty-acid metabolism	[766137:799254]	17	Radical_SAM; Thiolase_C; Thiolase_N AcetylCoA_hyd_C; Acyl-CoA_dh_1; B12-binding; BKACE; CoA_trans; HBT; LAM_C; Lys-AminoMut_A; OAM_dimer; Radical_SAM; Thiolase_C; Thiolase_N
Butyricimonas virosa	Main OP/low-BMD candidate	GCF_025148635.1	NZ_CP102269.1	6	aminobutyrate2Butyrate	Fermentation / energy metabolism	[1003701:1027910]	6	AcetylCoA_hyd_C; AcetylCoA_hydro; Fe-ADH; HpaB; HpaB_N
Butyricimonas virosa	Main OP/low-BMD candidate	GCF_025148635.1	NZ_CP102269.1	7	Arginine2putrescine	Amino-acid metabolism	[1660103:1685878]	3	Arginase; Orn_Arg_deC_N

Butyricimonas virosa	Main OP/low-BMD candidate	GCF_025148635.1	NZ_CP102269.1	8	Fumarate2succinate	Fermentation / energy metabolism	[2533787:2557295]	7	FAD_binding_2; Fer2_3; SucD_FumR; Succ_DH_flav_C
Butyricimonas virosa	Main OP/low-BMD candidate	GCF_025148635.1	NZ_CP102269.1	9	Respiratory_glycerol	Other / unassigned	[2632578:2656687]	6	DAO; FAD_binding_2; Fer2_BFD; Fer4_8
Butyricimonas virosa	Main OP/low-BMD candidate	GCF_025148635.1	NZ_CP102269.1	10	OD_fatty_acids	Lipid / fatty-acid metabolism	[2744953:2777432]	4	AcetylCoA_hyd_C; AcetylCoA_hydro; EKR; Fer4_16; PFOR_II; POR; POR_N; TPP_enzyme_C
Butyricimonas virosa	Main OP/low-BMD candidate	GCF_025148635.1	NZ_CP102269.1	11	PFOR_II_pathway	Fermentation / energy metabolism	[2753895:2777432]	2	AcetylCoA_hyd_C; AcetylCoA_hydro; EKR; Fer4_16; PFOR_II; POR; POR_N; TPP_enzyme_C
Butyricimonas virosa	Main OP/low-BMD candidate	GCF_025148635.1	NZ_CP102269.1	12	succinate2propionate	Fermentation / energy metabolism	[3309212:3333292]	6	Biotin_lipoyl; CT_Btheta; Glyoxalase_4;

Butyricimonas virosa	Main OP/low-BMD candidate	GCF_025148635.1	NZ_CP102269.1	13	NADH_dehydrogenase_I	Fermentation / energy metabolism	[4579795:4610706]	12	OAD_beta; OAD_gamma Complex1_30kDa; Complex1_49kDa; Fer4_7; NADHdh; Oxidored_q2; Oxidored_q3; Oxidored_q4; Oxidored_q6; Proton_antipo_M; Proton_antipo_N
Catenibacterium mitsuokai	Secondary OP candidate	GCF_025148785.1	NZ_CP102271.1	1	xanthine_dehydrogenase	Redox / nitrogen metabolism	[26180:49717]	9	Ald_Xan_dh_C; FAD_binding_5; Fer2; Fer2_2; MoCoBD_1; MoCoBD_2
Catenibacterium mitsuokai	Secondary OP candidate	GCF_025148785.1	NZ_CP102271.1	2	TPP_AA_metabolism	Amino-acid metabolism	[541938:569816]	9	ALS_ss_C; ILVD_EDD; IlvC; TPP_enzyme_C
Catenibacterium mitsuokai	Secondary OP candidate	GCF_025148785.1	NZ_CP102271.1	3	Putrescine2spermidine	Polyamine metabolism	[685744:708984]	13	Orn_DAP_Arg_deC; Sacchrp_dh_C; Spermine_synt_N; Spermine_synth

Catenibacterium mitsuokai	Secondary OP candidate	GCF_025148785.1	NZ_CP102271.1	4	GR_fatty_acids	Lipid / fatty-acid metabolism	[1609717:1638959]	14	FA_synthesis; Gly_8-9_TMA; PFL-like
Catenibacterium mitsuokai	Secondary OP candidate	GCF_025148785.1	NZ_CP102271.1	5	Rnf_complex	Fermentation / energy metabolism	[1648593:1673521]	9	Complex1_51K; FMN_bind; FeS; Fer4; Fer4_9; NQR2_RnfD_RnfE; Rnf-Nqr; RnfC_N; SLBB
Catenibacterium mitsuokai	Secondary OP candidate	GCF_025148785.1	NZ_CP102271.1	6	Pyruvate2acetate-formate	Fermentation / energy metabolism	[2051047:2074095]	5	PFL_acetate2; Radical_SAM
Catenibacterium mitsuokai	Secondary OP candidate	GCF_025148785.1	NZ_CP102271.1	7	Others_HGD_unassigned	Aromatic / phenolic metabolism	[2543645:2567872]	7	BcrAD_BadFG; HGD-D
Coprobacter fastidiosus	Main OP/low-BMD candidate	GCF_030296935.1	NZ_AP028032.1	1	Sulfate2PAPS	Sulfur metabolism	[57853:81194]	4	APS_kinase; GTP_EFTU; PAPS_reduct
Coprobacter fastidiosus	Main OP/low-BMD candidate	GCF_030296935.1	NZ_AP028032.1	2	Others_HGD_unassigned	Aromatic / phenolic	[278851:309032]	5	ACP_syn_III; ACP_syn_III_C;

						metabolism			BcrAD_BadFG; HGD-D
Coprobacter fastidiosus	Main OP/low-BMD candidate	GCF_030296935.1	NZ_AP028032.1	3	Phenylpyruvate_ferredoxin_oxidoreductase	Fermentation / energy metabolism	[375937:398121]	7	POR; POR_N; TPP_enzyme_C
Coprobacter fastidiosus	Main OP/low-BMD candidate	GCF_030296935.1	NZ_AP028032.1	4	porA	Fermentation / energy metabolism	[891865:914632]	5	Fer4_7; Ldh_1_C; Ldh_1_N; PFOR_II; POR; POR_N; TPP_enzyme_C
Coprobacter fastidiosus	Main OP/low-BMD candidate	GCF_030296935.1	NZ_AP028032.1	5	OD_lactate_related	Other / unassigned	[892136:917224]	5	Fer4_7; Ldh_1_C; Ldh_1_N; PFOR_II; POR; POR_N; TPP_enzyme_C
Coprobacter fastidiosus	Main OP/low-BMD candidate	GCF_030296935.1	NZ_AP028032.1	6	Rnf_complex	Fermentation / energy metabolism	[1220867:1245892]	8	Complex1_51K; FMN_bind; FeS; Fer4; NQR2_RnfD_RnfE; Rnf-Nqr; RnfC_N; SLBB
Coprobacter fastidiosus	Main OP/low-BMD candidate	GCF_030296935.1	NZ_AP028032.1	7	putative_2-oxoglutarate_ferredoxin_oxidoreductase	Redox / nitrogen metabolism	[2194838:2217712]	7	PFOR_II; POR; POR_N; TPP_enzyme_C

Coprobacter fastidiosus	Main OP/low-BMD candidate	GCF_030296935.1	NZ_AP028032.1	8	Fumarate2succinate	Fermentation / energy metabolism	[2386083:2409524]	3	FAD_binding_2; Fer2_3; SucD_FumR; Succ_DH_flav_C
Coprobacter fastidiosus	Main OP/low-BMD candidate	GCF_030296935.1	NZ_AP028032.1	10	PFOR_II_pathway	Fermentation / energy metabolism	[3030009:3053558]	4	EKR; ELFV_dehydrog; Fer4_16; PALP; PFOR_II; POR; POR_N; TPP_enzyme_C
Coprobacter fastidiosus	Main OP/low-BMD candidate	GCF_030296935.1	NZ_AP028032.1	9	OD_AA_metabolism	Amino-acid metabolism	[3023747:3064504]	8	CPSase_L_D2; EKR; ELFV_dehydrog; Fer4_16; PALP; PFOR_II; POR; POR_N; TPP_enzyme_C
Coprobacter fastidiosus	Main OP/low-BMD candidate	GCF_030296935.1	NZ_AP028032.1	11	TPP_AA_metabolism	Amino-acid metabolism	[3123676:3149660]	6	ALS_ss_C; ILVD_EDD; IlvC; TPP_enzyme_C
Coprobacter fastidiosus	Main OP/low-BMD candidate	GCF_030296935.1	NZ_AP028032.1	12	succinate2propionate	Fermentation / energy	[3188982:3213493]	10	Biotin_lipoyl; CT_Btheta; Glyoxalase_4;

						metabolism			OAD_beta; OAD_gamma
Coprococcus comes	Normal-BMD-associated comparator	GCF_016904155.1	NZ_CP070062.1	1	succinate2propionate	Fermentation / energy metabolism	[0:22101]	12	Biotin_lipoyl; CT_Vparvula; HMGL-like; OAD_beta; OAD_gamma; PYC_OADA
Coprococcus comes	Normal-BMD-associated comparator	GCF_016904155.1	NZ_CP070062.1	2	Others_HGD_unassigned	Aromatic / phenolic metabolism	[100094:123675]	3	BcrAD_BadFG; HGD-D
Coprococcus comes	Normal-BMD-associated comparator	GCF_016904155.1	NZ_CP070062.1	7	Others_HGD_unassigned	Aromatic / phenolic metabolism	[1232912:1249773]	8	BcrAD_BadFG; HGD-D
Coprococcus comes	Normal-BMD-associated comparator	GCF_016904155.1	NZ_CP070062.1	8	Others_HGD_unassigned	Aromatic / phenolic metabolism	[1684453:1707183]	4	BcrAD_BadFG; HGD-D
Coprococcus comes	Normal-BMD-associated comparator	GCF_016904155.1	NZ_CP070062.1	10	Others_HGD_unassigned	Aromatic / phenolic metabolism	[2678303:2710526]	10	Carboxyl_trans; HGD-D

Coprococcus comes	Normal-BMD-associated comparator	GCF_016904155.1	NZ_CP070062.1	3	Fumarate2succinate	Fermentation / energy metabolism	[446621:468867]	3	FAD_binding_2; Fer2_3; Succ_DH_flav_C
Coprococcus comes	Normal-BMD-associated comparator	GCF_016904155.1	NZ_CP070062.1	4	Glycine_reductase	Amino-acid metabolism	[522577:550157]	10	ACP_syn_III; FA_synthesis; GRDA; GRDB; Glycine_reductase
Coprococcus comes	Normal-BMD-associated comparator	GCF_016904155.1	NZ_CP070062.1	5	xanthine_dehydrogenase	Redox / nitrogen metabolism	[598135:621669]	9	Ald_Xan_dh_C; FAD_binding_5; Fer2; Fer2_2; MoCoBD_1; MoCoBD_2
Coprococcus comes	Normal-BMD-associated comparator	GCF_016904155.1	NZ_CP070062.1	6	Rnf_complex	Fermentation / energy metabolism	[627659:652672]	9	Complex1_51K; FMN_bind; FeS; Fer4; NQR2_RnfD_RnfE; Rnf-Nqr; RnfC_N; SLBB
Coprococcus comes	Normal-BMD-associated comparator	GCF_016904155.1	NZ_CP070062.1	9	proline2aminovalerate	Amino-acid metabolism	[2543890:2571330]	7	GRDB; Pro_racemase; Proline_reductase

Coprococcus comes	Normal-BMD-associated comparator	GCF_016904155.1	NZ_CP070062.1	11	PFOR_II_pathway	Fermentation / energy metabolism	[2880017:2903557]	3	EKR; Fer4_16; PFOR_II; POR; POR_N; TPP_enzyme_C
Coprococcus comes	Normal-BMD-associated comparator	GCF_016904155.1	NZ_CP070062.1	12	Glycine_cleavage	Fermentation / energy metabolism	[2894342:2918683]	8	Aminotran_5; GCV_H; GCV_T; GCV_T_C; GDC-P
Coprococcus comes	Normal-BMD-associated comparator	GCF_016904155.1	NZ_CP070062.1	13	Pyruvate2acetate-formate	Fermentation / energy metabolism	[2952561:2975739]	9	Butyrate_kinase; EC1_2-faecali; Gly_radical; HCDH; HCDH_N; PFL_acetate2; Radical_SAM; Thiolase_C; Thiolase_N
Coprococcus comes	Normal-BMD-associated comparator	GCF_016904155.1	NZ_CP070062.1	14	GR_fatty_acids	Lipid / fatty-acid metabolism	[2953486:2985967]	12	Acyl-CoA_dh_1; Butyrate_kinase; EC1_2-faecali; ETF; ETF_alpha; Gly_radical; HCDH; HCDH_N; PFL_acetate2;

Coprococcus comes	Normal-BMD-associated comparator	GCF_016904155.1	NZ_CP070062.1	15	acetate2butyrate	Fermentation / energy metabolism	[2957985:2987833]	12	Radical_SAM; Thiolase_C; Thiolase_N Acyl-CoA_dh_1; Butyrate_kinase; EC1_2-faecali; ETF; ETF_alpha; Gly_radical; HCDH; HCDH_N; PFL_acetate2; Radical_SAM; Thiolase_C; Thiolase_N
Coprococcus comes	Normal-BMD-associated comparator	GCF_016904155.1	NZ_CP070062.1	16	TPP_AA_metabolism	Amino-acid metabolism	[3170110:3195471]	7	ALS_ss_C; Homoserine_dh; TPP_enzyme_C
Coprococcus eutactus	Normal-BMD-associated comparator	GCF_020735705.1	NZ_CP085930.1	1	succinate2propionate	Fermentation / energy metabolism	[141180:166690]	12	Biotin_lipoyl; CT_Vparvula; HMGL-like; OAD_beta; OAD_gamma; PYC_OADA

Coprococcus eutactus	Normal-BMD-associated comparator	GCF_020735705.1	NZ_CP085930.1	2	Others_HGD_unassigned	Aromatic / phenolic metabolism	[184771:208992]	3	BcrAD_BadFG; HGD-D
Coprococcus eutactus	Normal-BMD-associated comparator	GCF_020735705.1	NZ_CP085930.1	3	xanthine_dehydrogenase	Redox / nitrogen metabolism	[323012:346581]	7	Ald_Xan_dh_C; FAD_binding_5; Fer2; Fer2_2; MoCoBD_1; MoCoBD_2
Coprococcus eutactus	Normal-BMD-associated comparator	GCF_020735705.1	NZ_CP085930.1	4	Phenylpyruvate_ferredoxin_oxidoreductase	Fermentation / energy metabolism	[827732:848229]	5	POR; POR_N; TPP_enzyme_C
Coprococcus eutactus	Normal-BMD-associated comparator	GCF_020735705.1	NZ_CP085930.1	7	Phenylpyruvate_ferredoxin_oxidoreductase	Fermentation / energy metabolism	[1705243:1727602]	7	OKR_DC_1_7_arginine; OKR_DC_1_C; POR; POR_N; TPP_enzyme_C
Coprococcus eutactus	Normal-BMD-associated comparator	GCF_020735705.1	NZ_CP085930.1	5	Adenylylsulfate_reductase	Sulfur metabolism	[1142377:1164451]	12	FAD_binding_2; Fer4_9
Coprococcus eutactus	Normal-BMD-associated comparator	GCF_020735705.1	NZ_CP085930.1	6	OD_AA_metabolism	Amino-acid metabolism	[1696733:1727602]	7	OKR_DC_1_7_arginine; OKR_DC_1_C; POR; POR_N; TPP_enzyme_C

Coprococcus eutactus	Normal-BMD-associated comparator	GCF_020735705.1	NZ_CP085930.1	8	Rnf_complex	Fermentation / energy metabolism	[2772874:2798485]	8	Complex1_51K; FMN_bind; FeS; Fer4; NQR2_RnfD_RnfE; Rnf-Nqr; RnfC_N; SLBB
Coprococcus eutactus	Normal-BMD-associated comparator	GCF_020735705.1	NZ_CP085930.1	9	Pyruvate2acetate-formate	Fermentation / energy metabolism	[2943176:2966506]	5	PFL_acetate2; Radical_SAM
Coprococcus eutactus	Normal-BMD-associated comparator	GCF_020735705.1	NZ_CP085930.1	10	acetate2butyrate	Fermentation / energy metabolism	[3048089:3074367]	10	Acyl-CoA_dh_1; EC1_2-faecali; ETF; ETF_alpha; HCDH; HCDH_N; Thiolase_C; Thiolase_N
Dialister invisus	Main OP/low-BMD candidate	GCF_000160055.1	NZ_GG698602.1	1	Fumarate2succinate	Fermentation / energy metabolism	[870949:893290]	2	FAD_binding_2; Fer2_3; Succ_DH_flav_C
Dialister invisus	Main OP/low-BMD candidate	GCF_000160055.1	NZ_GG698602.1	2	succinate2propionate	Fermentation / energy metabolism	[956833:992044]	13	AcetylCoA_hyd_C; AcetylCoA_hydro; B12-binding; Biotin_lipoyl;

									CT_Vparvula; Glyoxalase_4; MM_CoA_mutase; MeaB
Dialister invisus	Main OP/low-BMD candidate	GCF_000160055.1	NZ_GG698602.1	3	Others_HGD_unassigned	Aromatic / phenolic metabolism	[1588613:1612909]	4	BcrAD_BadFG; HGD-D
Dialister invisus	Main OP/low-BMD candidate	GCF_000160055.1	NZ_GG698602.1	4	PFOR_II_pathway	Fermentation / energy metabolism	[1761518:1785079]	4	EKR; Fer4_16; PFOR_II; POR; POR_N; TPP_enzyme_C
Dialister succinatiphilus	Main OP/low-BMD candidate	GCF_928721695.1	NZ_OV788641.1	1	OD_fatty_acids	Lipid / fatty-acid metabolism	[165240:191114]	5	HBT; HBT_2; PFOR_II; POR; POR_N
Dialister succinatiphilus	Main OP/low-BMD candidate	GCF_928721695.1	NZ_OV788641.1	2	succinate2propionate	Fermentation / energy metabolism	[375083:410047]	13	AcetylCoA_hyd_C; AcetylCoA_hydro; B12-binding; Biotin_lipoyl; CT_Vparvula; Glyoxalase_4;

									MM_CoA_mutase; MeaB
Dialister succinatiphilus	Main OP/low-BMD candidate	GCF_928721695.1	NZ_OV788641.1	3	putative_2-oxoglutarate_ferredoxin_oxidoreductase	Redox / nitrogen metabolism	[465544:488162]	2	PFOR_II; POR; POR_N; TPP_enzyme_C
Dialister succinatiphilus	Main OP/low-BMD candidate	GCF_928721695.1	NZ_OV788641.1	4	Ech_complex	Other / unassigned	[1115116:1139921]	6	Complex1_30kDa; Complex1_49kDa; NADHdh; Oxidored_q6; Proton_antipo_M; Proton_antipo_N
Dialister succinatiphilus	Main OP/low-BMD candidate	GCF_928721695.1	NZ_OV788641.1	5	Others_HGD_unassigned	Aromatic / phenolic metabolism	[1240817:1265107]	4	BcrAD_BadFG; HGD-D
Dialister succinatiphilus	Main OP/low-BMD candidate	GCF_928721695.1	NZ_OV788641.1	6	Putrescine2spermidine	Polyamine metabolism	[1275522:1296380]	6	Spermine_synt_N; Spermine_synth
Dorea formicigenerans	Normal-BMD-associated comparator	GCF_051626395.1	NZ_CP145334.1	1	uric_acid2SCFA	Other / unassigned	[675132:723165]	23	Ald_Xan_dh_C; Amidohydro_1; FAD_binding_5; Fer2; Fer2_2; MoCoBD_1;

									MoCoBD_2; PALP; Peptidase_M20; Xan_ur_permease
Dorea formicigenerans	Normal-BMD-associated comparator	GCF_051626395.1	NZ_CP145334.1	2	xanthine_dehydrogenase	Redox / nitrogen metabolism	[691919:712797]	12	Ald_Xan_dh_C; Amidohydro_1; FAD_binding_5; Fer2; Fer2_2; MoCoBD_1; MoCoBD_2; PALP; Peptidase_M20; Xan_ur_permease
Dorea formicigenerans	Normal-BMD-associated comparator	GCF_051626395.1	NZ_CP145334.1	10	xanthine_dehydrogenase	Redox / nitrogen metabolism	[2408922:2432504]	6	Ald_Xan_dh_C; FAD_binding_5; Fer2; Fer2_2; MoCoBD_1; MoCoBD_2
Dorea formicigenerans	Normal-BMD-associated comparator	GCF_051626395.1	NZ_CP145334.1	3	Glycine_cleavage	Fermentation / energy metabolism	[744375:768669]	9	Aminotran_5; GCV_H; GCV_T; GCV_T_C; GDC-P
Dorea formicigenerans	Normal-BMD-associated comparator	GCF_051626395.1	NZ_CP145334.1	4	Fumarate2succinate	Fermentation / energy metabolism	[808878:831133]	5	FAD_binding_2; Fer2_3; Succ_DH_flav_C

Dorea formicigenerans	Normal-BMD-associated comparator	GCF_051626395.1	NZ_CP145334.1	5	TPP_fatty_acids	Lipid / fatty-acid metabolism	[1823099:1850008]	11	ECH_1; HBT; TPP_enzyme_C
Dorea formicigenerans	Normal-BMD-associated comparator	GCF_051626395.1	NZ_CP145334.1	6	PFOR_II_pathway	Fermentation / energy metabolism	[1937559:1961099]	5	EKR; Fer4_16; PFOR_II; POR; POR_N; TPP_enzyme_C
Dorea formicigenerans	Normal-BMD-associated comparator	GCF_051626395.1	NZ_CP145334.1	7	Phenylpyruvate_ferredoxin_oxidoreductase	Fermentation / energy metabolism	[2161352:2183004]	8	POR; POR_N; TPP_enzyme_C
Dorea formicigenerans	Normal-BMD-associated comparator	GCF_051626395.1	NZ_CP145334.1	8	Phenylpyruvate_ferredoxin_oxidoreductase	Fermentation / energy metabolism	[2261364:2283688]	9	POR; POR_N; TPP_enzyme_C
Dorea formicigenerans	Normal-BMD-associated comparator	GCF_051626395.1	NZ_CP145334.1	9	Glycine_reductase	Amino-acid metabolism	[2369649:2397033]	12	ACP_syn_III; FA_synthesis; GRDA; GRDB; Glycine_reductase
Dorea formicigenerans	Normal-BMD-associated comparator	GCF_051626395.1	NZ_CP145334.1	11	Others_HGD_unassigned	Aromatic / phenolic	[2545151:2579351]	9	BcrAD_BadFG; ETF; ETF_alpha; HGD-D

						metabolism			
Dorea formicigenerans	Normal-BMD-associated comparator	GCF_051626395.1	NZ_CP145334.1	16	Others_HGD_unassigned	Aromatic / phenolic metabolism	[3477496:3507507]	13	BcrAD_BadFG; HGD-D; Hacid_dh_C
Dorea formicigenerans	Normal-BMD-associated comparator	GCF_051626395.1	NZ_CP145334.1	12	Pyruvate2acetate-formate	Fermentation / energy metabolism	[2637564:2660676]	7	PFL_acetate2; Radical_SAM
Dorea formicigenerans	Normal-BMD-associated comparator	GCF_051626395.1	NZ_CP145334.1	13	Rnf_complex	Fermentation / energy metabolism	[2748978:2773990]	14	Biotin_lipoyl; Complex1_51K; FMN_bind; FeS; Fer4; HMGL-like; NQR2_RnfD_RnfE; OAD_beta; OAD_gamma; PYC_OADA; Rnf-Nqr; RnfC_N; SLBB
Dorea formicigenerans	Normal-BMD-associated comparator	GCF_051626395.1	NZ_CP145334.1	14	succinate2propionate	Fermentation / energy metabolism	[2759215:2786672]	15	Biotin_lipoyl; CT_Vparvula; Complex1_51K; FMN_bind; Fer4;

									HMGL-like; NQR2_RnfD_RnfE; OAD_beta; OAD_gamma; PYC_OADA; Rnf-Nqr; RnfC_N; SLBB
Dorea formicigenerans	Normal-BMD-associated comparator	GCF_051626395.1	NZ_CP145334.1	15	TPP_AA_metabolism	Amino-acid metabolism	[2963242:2993473]	9	ALS_ss_C; Homoserine_dh; TPP_enzyme_C
Dorea longicatena	Normal-BMD-associated comparator	GCF_023008345.1	NZ_CP094679.1	1	succinate2propionate	Fermentation / energy metabolism	[95437:126278]	16	Biotin_lipoyl; CT_Vparvula; Complex1_51K; FMN_bind; Fer4; HMGL-like; NQR2_RnfD_RnfE; OAD_beta; OAD_gamma; PYC_OADA; Rnf-Nqr; RnfC_N; SLBB
Dorea longicatena	Normal-BMD-associated comparator	GCF_023008345.1	NZ_CP094679.1	2	Rnf_complex	Fermentation / energy	[111826:136840]	14	Biotin_lipoyl; Complex1_51K; FMN_bind; FeS; Fer4;

						metabolism			Fer4_9; HMGL-like; NQR2_RnfD_RnfE; OAD_beta; OAD_gamma; PYC_OADA; Rnf-Nqr; RnfC_N; SLBB
Dorea longicatena	Normal-BMD-associated comparator	GCF_023008345.1	NZ_CP094679.1	3	Others_HGD_unassigned	Aromatic / phenolic metabolism	[422605:444844]	4	BcrAD_BadFG; HGD-D
Dorea longicatena	Normal-BMD-associated comparator	GCF_023008345.1	NZ_CP094679.1	10	Others_HGD_unassigned	Aromatic / phenolic metabolism	[1355323:1388670]	10	BcrAD_BadFG; FGGY_C; HGD-D
Dorea longicatena	Normal-BMD-associated comparator	GCF_023008345.1	NZ_CP094679.1	14	Others_HGD_unassigned	Aromatic / phenolic metabolism	[2851456:2874412]	6	BcrAD_BadFG; HGD-D
Dorea longicatena	Normal-BMD-associated comparator	GCF_023008345.1	NZ_CP094679.1	4	Pyruvate2acetate-formate	Fermentation / energy metabolism	[467261:490423]	7	PFL_acetate2; Radical_SAM

Dorea longicatena	Normal-BMD-associated comparator	GCF_023008345.1	NZ_CP094679.1	5	xanthine_dehydrogenase	Redox / nitrogen metabolism	[565664:589233]	6	Ald_Xan_dh_C; FAD_binding_5; Fer2; Fer2_2; MoCoBD_1; MoCoBD_2
Dorea longicatena	Normal-BMD-associated comparator	GCF_023008345.1	NZ_CP094679.1	7	Flavoenzyme_AA_peptides_catabolism	Other / unassigned	[667365:698528]	8	ALS_ss_C; Homoserine_dh; TPP_enzyme_C
Dorea longicatena	Normal-BMD-associated comparator	GCF_023008345.1	NZ_CP094679.1	6	TPP_AA_metabolism	Amino-acid metabolism	[667365:698528]	8	ALS_ss_C; Homoserine_dh; TPP_enzyme_C
Dorea longicatena	Normal-BMD-associated comparator	GCF_023008345.1	NZ_CP094679.1	8	Glycine_reductase	Amino-acid metabolism	[1079206:1106586]	12	ACP_syn_III; FA_synthesis; GRDA; GRDB; Glycine_reductase
Dorea longicatena	Normal-BMD-associated comparator	GCF_023008345.1	NZ_CP094679.1	9	PFOR_II_pathway	Fermentation / energy metabolism	[1097752:1121295]	3	EKR; Fer4_16; PFOR_II; POR; POR_N; TPP_enzyme_C
Dorea longicatena	Normal-BMD-associated comparator	GCF_023008345.1	NZ_CP094679.1	11	proline2aminovalerate	Amino-acid metabolism	[1453235:1480828]	12	GRDB; Pro_racemase; Proline_reductase

Dorea longicatena	Normal-BMD-associated comparator	GCF_023008345.1	NZ_CP094679.1	12	gallic_acid_met	Aromatic / phenolic metabolism	[1972561:1994662]	8	Flavoprotein; gallic_acid
Dorea longicatena	Normal-BMD-associated comparator	GCF_023008345.1	NZ_CP094679.1	13	Phenylpyruvate_ferredoxin_oxidoreductase	Fermentation / energy metabolism	[2462696:2481821]	7	POR; POR_N; TPP_enzyme_C
Dorea longicatena	Normal-BMD-associated comparator	GCF_023008345.1	NZ_CP094679.1	15	Phenylpyruvate_ferredoxin_oxidoreductase	Fermentation / energy metabolism	[3343313:3365631]	6	POR; POR_N; TPP_enzyme_C
Dorea longicatena	Normal-BMD-associated comparator	GCF_023008345.1	NZ_CP094679.1	16	Glycine_cleavage	Fermentation / energy metabolism	[3422856:3442572]	11	Aminotran_5; GCV_H; GCV_T; GCV_T_C; GDC-P
Eggerthella lenta	Main OP/low-BMD candidate	GCF_021378705.1	NZ_CP089335.1	1	Phenylpyruvate_ferredoxin_oxidoreductase	Fermentation / energy metabolism	[62264:84431]	4	POR; POR_N; TPP_enzyme_C
Eggerthella lenta	Main OP/low-BMD candidate	GCF_021378705.1	NZ_CP089335.1	17	Phenylpyruvate_ferredoxin_oxidoreductase	Fermentation / energy	[2087374:2109734]	13	Complex1_30kDa; Complex1_49kDa; Oxidored_q6; POR;

						metabolism		POR_N; TPP_enzyme_C
Eggerthella lenta	Main OP/low-BMD candidate	GCF_021378705.1	NZ_CP089335.1	2	Molybdopterin_dependent_oxidoreductase	Redox / nitrogen metabolism	[141548:164992]	6	DmsC; Fer4_11; Fer4_4; Molybdop_Fe4S4; Molybdopterin; Molydop_binding; Nitrate_red_del; NrfD
Eggerthella lenta	Main OP/low-BMD candidate	GCF_021378705.1	NZ_CP089335.1	3	Molybdopterin_dependent_oxidoreductase	Redox / nitrogen metabolism	[337942:362015]	6	Fer4_11; Fer4_4; Molybdop_Fe4S4; Molybdopterin; Molydop_binding; NrfD
Eggerthella lenta	Main OP/low-BMD candidate	GCF_021378705.1	NZ_CP089335.1	4	Molybdopterin_dependent_oxidoreductase	Redox / nitrogen metabolism	[383045:411991]	13	Fer4_11; Fer4_4; Molydop_binding; NrfD
Eggerthella lenta	Main OP/low-BMD candidate	GCF_021378705.1	NZ_CP089335.1	6	Molybdopterin_dependent_oxidoreductase	Redox / nitrogen metabolism	[840209:864836]	10	DmsC; Fer4_11; Fer4_4; Molybdop_Fe4S4; Molybdopterin; Molydop_binding; Nitrate_red_del

Eggerthella lenta	Main OP/low-BMD candidate	GCF_021378705.1	NZ_CP089335.1	7	Molybdopterin_dependent_oxidoreductase	Redox / nitrogen metabolism	[862977:923280]	27	DmsC; Fer4_11; Fer4_4; Molybdop_Fe4S4; Molybdopterin; Molydop_binding; Nitrate_red_del; NrfD
Eggerthella lenta	Main OP/low-BMD candidate	GCF_021378705.1	NZ_CP089335.1	8	Molybdopterin_dependent_oxidoreductase	Redox / nitrogen metabolism	[922650:1015710]	38	DmsC; Fer4_11; Fer4_4; Molybdop_Fe4S4; Molybdopterin; Molydop_binding; Nitrate_red_del
Eggerthella lenta	Main OP/low-BMD candidate	GCF_021378705.1	NZ_CP089335.1	9	Molybdopterin_dependent_oxidoreductase	Redox / nitrogen metabolism	[1103477:1141207]	13	DmsC; Fer4_11; Fer4_4; Molybdop_Fe4S4; Molybdopterin; Molydop_binding; Nitrate_red_del
Eggerthella lenta	Main OP/low-BMD candidate	GCF_021378705.1	NZ_CP089335.1	13	Molybdopterin_dependent_oxidoreductase	Redox / nitrogen metabolism	[1744078:1770118]	14	ACD1_car; ALS_ss_C; Acyl-CoA_dh_1; Fer4_11; Fer4_4; HBT; ILVD_EDD; IlvC;

									Molybdopterin; Molydop_binding; NrfD; TPP_enzyme_C; baiH
Eggerthella lenta	Main OP/low-BMD candidate	GCF_021378705.1	NZ_CP089335.1	16	Molybdopterin_dependent_oxidoreductase	Redox / nitrogen metabolism	[2046597:2071412]	6	DmsC; Fer4_11; Fer4_4; Molybdop_Fe4S4; Molybdopterin; Molydop_binding; Nitrate_red_del; NrfD
Eggerthella lenta	Main OP/low-BMD candidate	GCF_021378705.1	NZ_CP089335.1	19	Molybdopterin_dependent_oxidoreductase	Redox / nitrogen metabolism	[2487822:2512189]	9	DmsC; Fer4_11; Fer4_4; Molybdopterin; Molydop_binding; Nitrate_red_del
Eggerthella lenta	Main OP/low-BMD candidate	GCF_021378705.1	NZ_CP089335.1	22	Molybdopterin_dependent_oxidoreductase	Redox / nitrogen metabolism	[3302912:3326172]	12	DmsC; Fer4_11; Fer4_4; Molybdop_Fe4S4; Molybdopterin; Molydop_binding
Eggerthella lenta	Main OP/low-BMD candidate	GCF_021378705.1	NZ_CP089335.1	24	Molybdopterin_dependent_oxidoreductase	Redox / nitrogen	[3573113:3601494]	8	Fer4_11; Fer4_4; Molybdop_Fe4S4; Molydop_binding

						metabolism			
Eggerthella lenta	Main OP/low-BMD candidate	GCF_021378705.1	NZ_CP089335.1	5	Flavoenzyme_lipids_catabolism	Lipid / fatty-acid metabolism	[760027:787783]	11	adh_short_C2; baiH
Eggerthella lenta	Main OP/low-BMD candidate	GCF_021378705.1	NZ_CP089335.1	14	Flavoenzyme_lipids_catabolism	Lipid / fatty-acid metabolism	[1753463:1779713]	16	ACD1_car; Acyl-CoA_dh_1; Fer4_11; Fer4_4; HBT; Molybdopterin; Molydop_binding; NrfD; baiH
Eggerthella lenta	Main OP/low-BMD candidate	GCF_021378705.1	NZ_CP089335.1	23	Flavoenzyme_lipids_catabolism	Lipid / fatty-acid metabolism	[3406576:3442609]	15	ACD1_car; ACP_syn_III_C; ECH_1; baiH
Eggerthella lenta	Main OP/low-BMD candidate	GCF_021378705.1	NZ_CP089335.1	10	Rnf_complex	Fermentation / energy metabolism	[1195555:1220586]	10	Complex1_51K; FMN_bind; FeS; Fer4_9; NQR2_RnfD_RnfE; Rnf-Nqr; RnfC_N; SLBB

Eggerthella lenta	Main OP/low-BMD candidate	GCF_021378705.1	NZ_CP089335.1	11	Arginine2_Hcarbonate	Amino-acid metabolism	[1659744:1681719]	5	AA_kinase; Amidinotransf; OTCace; OTCace_N
Eggerthella lenta	Main OP/low-BMD candidate	GCF_021378705.1	NZ_CP089335.1	21	Arginine2_Hcarbonate	Amino-acid metabolism	[3199411:3227628]	8	AA_kinase; Amidinotransf; OTCace; OTCace_N
Eggerthella lenta	Main OP/low-BMD candidate	GCF_021378705.1	NZ_CP089335.1	12	TPP_AA_metabolism	Amino-acid metabolism	[1735913:1761014]	9	ALS_ss_C; Fer4_11; Fer4_4; ILVD_EDD; IlvC; Molybdopterin; Molydop_binding; NrfD; TPP_enzyme_C
Eggerthella lenta	Main OP/low-BMD candidate	GCF_021378705.1	NZ_CP089335.1	15	Others_HGD_unassigned	Aromatic / phenolic metabolism	[1953447:1977021]	9	BcrAD_BadFG; HGD-D
Eggerthella lenta	Main OP/low-BMD candidate	GCF_021378705.1	NZ_CP089335.1	20	Others_HGD_unassigned	Aromatic / phenolic metabolism	[3172340:3197032]	3	BcrAD_BadFG; HGD-D
Eggerthella lenta	Main OP/low-BMD candidate	GCF_021378705.1	NZ_CP089335.1	18	Ech_complex	Other / unassigned	[2097187:2122276]	12	Complex1_30kDa; Complex1_49kDa;

								NADHdh; Oxidored_q6; POR; POR_N; Proton_antipo_M; Proton_antipo_N; TPP_enzyme_C
Faecalibacterium prausnitzii	Contradictory/functional comparator	GCF_049532955.1	NZ_CP186692.1	1	Others_HGD_unassigned	Aromatic / phenolic metabolism	[263248:287650]	5	BcrAD_BadFG; HGD-D
Faecalibacterium prausnitzii	Contradictory/functional comparator	GCF_049532955.1	NZ_CP186692.1	2	Putrescine2spermidine	Polyamine metabolism	[468594:492866]	8	Orn_DAP_Arg_deC; Sacchrp_dh_C; Spermine_synt_N; Spermine_synth
Faecalibacterium prausnitzii	Contradictory/functional comparator	GCF_049532955.1	NZ_CP186692.1	3	Pyruvate2acetate-formate	Fermentation / energy metabolism	[690296:713285]	5	PFL_acetate2; Radical_SAM
Faecalibacterium prausnitzii	Contradictory/functional comparator	GCF_049532955.1	NZ_CP186692.1	4	OD_AA_metabolism	Amino-acid metabolism	[774406:812677]	12	ALS_ss_C; Cys_Met_Meta_PP; ILVD_EDD; IlvC; PALP; POR_N

Faecalibacterium prausnitzii	Contradictory/functional comparator	GCF_049532955.1	NZ_CP186692.1	5	Fumarate2succinate	Fermentation / energy metabolism	[1482257:1504509]	2	FAD_binding_2; Fer2_3; Succ_DH_flav_C
Faecalibacterium prausnitzii	Contradictory/functional comparator	GCF_049532955.1	NZ_CP186692.1	6	Rnf_complex	Fermentation / energy metabolism	[1588479:1613511]	11	FMN_bind; FeS; Fer4; NQR2_RnfD_RnfE; Rnf-Nqr; RnfC_N
Faecalibacterium prausnitzii	Contradictory/functional comparator	GCF_049532955.1	NZ_CP186692.1	7	gallic_acid_met	Aromatic / phenolic metabolism	[1894172:1916241]	3	Flavoprotein; gallic_acid
Faecalibacterium prausnitzii	Contradictory/functional comparator	GCF_049532955.1	NZ_CP186692.1	8	Bilirubin_reductase	Other / unassigned	[2623068:2644704]	7	BilR_short; Flavodoxin_3
Faecalibacterium prausnitzii	Contradictory/functional comparator	GCF_049532955.1	NZ_CP186692.1	9	acetate2butyrate	Fermentation / energy metabolism	[2773696:2799996]	17	Acyl-CoA_dh_1; Ald_Xan_dh_C; EC1_2-faecali; ETF; ETF_alpha; FAD_binding_5; Fer2; Fer2_2; HCDH; HCDH_N; MoCoBD_1; MoCoBD_2;

Faecalibacterium prausnitzii	Contradictory/functional comparator	GCF_049532955.1	NZ_CP186692.1	10	xanthine_dehydrogenase	Redox / nitrogen metabolism	[2785286:2808838]	10	Thiolase_C; Thiolase_N Acyl-CoA_dh_1; Ald_Xan_dh_C; ETF; ETF_alpha; FAD_binding_5; Fer2; Fer2_2; HCDH; HCDH_N; MoCoBD_1; MoCoBD_2
Megamonas funiformis	Normal-BMD-associated comparator	GCF_010669225.1	NZ_CP048627.1	1	Glycine_cleavage	Fermentation / energy metabolism	[170806:195091]	9	Aminotran_5; GCV_H; GCV_T; GCV_T_C; GDC-P
Megamonas funiformis	Normal-BMD-associated comparator	GCF_010669225.1	NZ_CP048627.1	2	Others_HGD_unassigned	Aromatic / phenolic metabolism	[427754:451978]	7	BcrAD_BadFG; HGD-D
Megamonas funiformis	Normal-BMD-associated comparator	GCF_010669225.1	NZ_CP048627.1	3	GR_fatty_acids	Lipid / fatty-acid metabolism	[613862:649950]	9	ACP_syn_III_C; FA_synthesis; Gly_8-9_TMA; HBT; Ketoacyl-synt_C;

									PFL-like; Thiolase_N; adh_short_C2
Megamonas funiformis	Normal-BMD-associated comparator	GCF_010669225.1	NZ_CP048627.1	5	Arginine2putrescine	Amino-acid metabolism	[740342:764341]	11	AdoMet_dc; Arginase; OKR_DC_1_7_arginine; Orn_DAP_Arg_deC; Sacchrp_dh_C; Spermine_synt_N; Spermine_synth
Megamonas funiformis	Normal-BMD-associated comparator	GCF_010669225.1	NZ_CP048627.1	4	Putrescine2spermidine	Polyamine metabolism	[740342:766833]	11	AdoMet_dc; Arginase; OKR_DC_1_7_arginine; Orn_DAP_Arg_deC; Sacchrp_dh_C; Spermine_synt_N; Spermine_synth
Megamonas funiformis	Normal-BMD-associated comparator	GCF_010669225.1	NZ_CP048627.1	6	PFOR_II_pathway	Fermentation / energy metabolism	[1020278:1043851]	12	EKR; Fer4_16; PFOR_II; POR; POR_N; TPP_enzyme_C
Megamonas funiformis	Normal-BMD-associated comparator	GCF_010669225.1	NZ_CP048627.1	7	xanthine_dehydrogenase	Redox / nitrogen	[1693739:1717301]	6	Ald_Xan_dh_C; FAD_binding_5; Fer2;

						metabolism			Fer2_2; MoCoBD_1; MoCoBD_2
Megamonas funiformis	Normal-BMD-associated comparator	GCF_010669225.1	NZ_CP048627.1	8	TPP_AA_metabolism	Amino-acid metabolism	[1770835:1805352]	12	ALS_ss_C; Homoserine_dh; IlvC; TPP_enzyme_C
Megamonas funiformis	Normal-BMD-associated comparator	GCF_010669225.1	NZ_CP048627.1	9	Fumarate2succinate	Fermentation / energy metabolism	[1809219:1831774]	7	FAD_binding_2; Fer2_3; Succ_DH_flav_C
Megamonas funiformis	Normal-BMD-associated comparator	GCF_010669225.1	NZ_CP048627.1	10	succinate2propionate	Fermentation / energy metabolism	[1931077:1958397]	9	AcetylCoA_hyd_C; AcetylCoA_hydro; B12-binding; Biotin_lipoyl; CT_Vparvula; Glyoxalase_4; MM_CoA_mutase; MeaB
Megamonas funiformis	Normal-BMD-associated comparator	GCF_010669225.1	NZ_CP048627.1	11	porA	Fermentation / energy metabolism	[2470054:2492739]	12	Fer4_7; PFOR_II; POR; POR_N; TPP_enzyme_C

Megamonas hypermegale	Normal-BMD-associated comparator	GCF_963931125.1	NZ_OZ006974.1	1	xanthine_dehydrogenase	Redox / nitrogen metabolism	[62388:85920]	6	Ald_Xan_dh_C; FAD_binding_5; Fer2; Fer2_2; MoCoBD_1; MoCoBD_2
Megamonas hypermegale	Normal-BMD-associated comparator	GCF_963931125.1	NZ_OZ006974.1	2	PFOR_II_pathway	Fermentation / energy metabolism	[169818:193343]	8	EKR; Fer4_16; PFOR_II; POR; POR_N; TPP_enzyme_C
Megamonas hypermegale	Normal-BMD-associated comparator	GCF_963931125.1	NZ_OZ006974.1	3	Putrescine2spermidine	Polyamine metabolism	[287662:315811]	14	AdoMet_dc; Arginase; OKR_DC_1_7_arginine; Orn_DAP_Arg_deC; Sacchrp_dh_C; Spermine_synt_N; Spermine_synth
Megamonas hypermegale	Normal-BMD-associated comparator	GCF_963931125.1	NZ_OZ006974.1	4	Arginine2putrescine	Amino-acid metabolism	[291834:315811]	13	AdoMet_dc; Arginase; OKR_DC_1_7_arginine; Orn_DAP_Arg_deC; Sacchrp_dh_C; Spermine_synt_N; Spermine_synth

Megamonas hypermegale	Normal-BMD-associated comparator	GCF_963931125.1	NZ_OZ006974.1	5	fatty_acids-unassigned	Lipid / fatty-acid metabolism	[431301:462680]	10	HBT; Thiolase_C; Thiolase_N; ketoacyl-synt
Megamonas hypermegale	Normal-BMD-associated comparator	GCF_963931125.1	NZ_OZ006974.1	6	Others_HGD_unassigned	Aromatic / phenolic metabolism	[541770:565991]	4	BcrAD_BadFG; HGD-D
Megamonas hypermegale	Normal-BMD-associated comparator	GCF_963931125.1	NZ_OZ006974.1	7	Glycine_cleavage	Fermentation / energy metabolism	[869010:893426]	9	Aminotran_5; GCV_H; GCV_T; GCV_T_C; GDC-P
Megamonas hypermegale	Normal-BMD-associated comparator	GCF_963931125.1	NZ_OZ006974.1	8	porA	Fermentation / energy metabolism	[1124458:1147143]	6	Fer4_7; PFOR_II; POR; POR_N; TPP_enzyme_C
Megamonas hypermegale	Normal-BMD-associated comparator	GCF_963931125.1	NZ_OZ006974.1	9	succinate2propionate	Fermentation / energy metabolism	[1716283:1743626]	9	AcetylCoA_hyd_C; AcetylCoA_hydro; B12-binding; Biotin_lipoyl; CT_Vparvula; Glyoxalase_4;

									MM_CoA_mutase; MeaB
Megamonas hypermegale	Normal-BMD-associated comparator	GCF_963931125.1	NZ_OZ006974.1	10	Pyruvate2acetate-formate	Fermentation / energy metabolism	[1783109:1806174]	5	PFL_acetate2; Radical_SAM
Megamonas hypermegale	Normal-BMD-associated comparator	GCF_963931125.1	NZ_OZ006974.1	11	TPP_AA_metabolism	Amino-acid metabolism	[1939365:1973627]	14	ALS_ss_C; Homoserine_dh; IlvC; TPP_enzyme_C
Megamonas hypermegale	Normal-BMD-associated comparator	GCF_963931125.1	NZ_OZ006974.1	12	Fumarate2succinate	Fermentation / energy metabolism	[2036459:2059014]	3	FAD_binding_2; Fer2_3; Succ_DH_flav_C
Odoribacter splanchnicus	Main OP/low-BMD candidate	GCF_000190535.1	NC_015160.1	1	PFOR_II_pathway	Fermentation / energy metabolism	[195294:218831]	6	EKR; Fer4_16; PFOR_II; POR; POR_N; TPP_enzyme_C
Odoribacter splanchnicus	Main OP/low-BMD candidate	GCF_000190535.1	NC_015160.1	2	Sulfate2PAPS	Sulfur metabolism	[653494:676566]	6	APS_kinase; GTP_EFTU; PAPS_reduct
Odoribacter splanchnicus	Main OP/low-BMD candidate	GCF_000190535.1	NC_015160.1	3	succinate2propionate	Fermentation / energy	[1240502:1264666]	10	Biotin_lipoyl; CT_Btheta;

						metabolism			Glyoxalase_4; OAD_beta; OAD_gamma
Odoribacter splanchnicus	Main OP/low-BMD candidate	GCF_000190535.1	NC_015160.1	4	Rnf_complex	Fermentation / energy metabolism	[1639827:1665170]	11	Complex1_51K; FMN_bind; FeS; Fer4; Fer4_9; NQR2_RnfD_RnfE; Rnf-Nqr; RnfC_N; SLBB
Odoribacter splanchnicus	Main OP/low-BMD candidate	GCF_000190535.1	NC_015160.1	5	NADH_dehydrogenase_I	Fermentation / energy metabolism	[1708005:1738016]	14	Complex1_30kDa; Complex1_49kDa; Fer4_7; NADHdh; Oxidored_q2; Oxidored_q3; Oxidored_q4; Oxidored_q6; Proton_antipo_M; Proton_antipo_N
Odoribacter splanchnicus	Main OP/low-BMD candidate	GCF_000190535.1	NC_015160.1	6	Putrescine2spermidine	Polyamine metabolism	[1927645:1949996]	5	Orn_DAP_Arg_deC; Sacchrp_dh_C

Odoribacter splanchnicus	Main OP/low-BMD candidate	GCF_000190535.1	NC_015160.1	7	aminobutyrate2Butyrate	Fermentation / energy metabolism	[2445768:2470007]	10	AcetylCoA_hyd_C; AcetylCoA_hydro; Fe-ADH; HpaB; HpaB_N
Odoribacter splanchnicus	Main OP/low-BMD candidate	GCF_000190535.1	NC_015160.1	8	Phenylpyruvate_ferredoxin_oxidoreductase	Fermentation / energy metabolism	[2555837:2578060]	5	POR; POR_N; TPP_enzyme_C
Odoribacter splanchnicus	Main OP/low-BMD candidate	GCF_000190535.1	NC_015160.1	9	putative_2-oxoglutarate_ferredoxin_oxidoreductase	Redox / nitrogen metabolism	[2747386:2770305]	2	PFOR_II; POR; POR_N; TPP_enzyme_C
Odoribacter splanchnicus	Main OP/low-BMD candidate	GCF_000190535.1	NC_015160.1	10	TPP_AA_metabolism	Amino-acid metabolism	[3441811:3473413]	8	ALS_ss_C; ILVD_EDD; IlvC; PALP; TPP_enzyme_C; Thr_dehydrat_C
Odoribacter splanchnicus	Main OP/low-BMD candidate	GCF_000190535.1	NC_015160.1	11	Lysine_degradation	Amino-acid metabolism	[3754860:3784825]	12	Acyl-CoA_dh_1; B12-binding; BKACE; CoA_trans; EC1_2-faecali; ETF; ETF_alpha; HBT; HCDH; HCDH_N;

									LAM_C; Lys-AminoMut_A; OAM_dimer; Radical_SAM
Odoribacter splanchnicus	Main OP/low-BMD candidate	GCF_000190535.1	NC_015160.1	12	acetate2butyrate	Fermentation / energy metabolism	[3764913:3789611]	11	Acyl-CoA_dh_1; B12-binding; BKACE; CoA_trans; EC1_2-faecali; ETF; ETF_alpha; HBT; HCDH; HCDH_N; LAM_C; Lys-AminoMut_A; OAM_dimer; Radical_SAM
Odoribacter splanchnicus	Main OP/low-BMD candidate	GCF_000190535.1	NC_015160.1	13	porA	Fermentation / energy metabolism	[3796767:3819513]	5	Fer4_7; PFOR_II; POR; POR_N; TPP_enzyme_C
Odoribacter splanchnicus	Main OP/low-BMD candidate	GCF_000190535.1	NC_015160.1	14	Fumarate2succinate	Fermentation / energy metabolism	[3834406:3857959]	9	FAD_binding_2; Fer2_3; SucD_FumR; Succ_DH_flav_C

Odoribacter splanchnicus	Main OP/low-BMD candidate	GCF_000190535.1	NC_015160.1	15	Respiratory_glycerol	Other / unassigned	[3936761:3966715]	10	DAO; FAD_binding_2; Fer2_BFD; Fer4_8
Oscillibacter valericigenes	Main OP/low-BMD candidate	GCF_000283575.1	NC_016048.1	1	Rnf_complex	Fermentation / energy metabolism	[50428:75602]	9	Complex1_51K; FMN_bind; FeS; Fer4; Fer4_9; NQR2_RnfD_RnfE; Rnf-Nqr; RnfC_N; SLBB
Oscillibacter valericigenes	Main OP/low-BMD candidate	GCF_000283575.1	NC_016048.1	2	OD_fatty_acids	Lipid / fatty-acid metabolism	[85307:114837]	8	AcetylCoA_hyd_C; EKR; Fer4_16; HpaB; PFOR_II; POR; POR_N; TPP_enzyme_C
Oscillibacter valericigenes	Main OP/low-BMD candidate	GCF_000283575.1	NC_016048.1	4	OD_fatty_acids	Lipid / fatty-acid metabolism	[334648:360990]	7	Acyl-CoA_dh_1; EKR; Fer4_16; HpaB; PFOR_II; POR; POR_N; TPP_enzyme_C
Oscillibacter valericigenes	Main OP/low-BMD candidate	GCF_000283575.1	NC_016048.1	6	OD_fatty_acids	Lipid / fatty-acid metabolism	[430876:466681]	11	PFOR_II; POR; POR_N; adh_short_C2

Oscillibacter valericigenes	Main OP/low-BMD candidate	GCF_000283575.1	NC_016048.1	18	OD_fatty_acids	Lipid / fatty-acid metabolism	[2702044:2729163]	9	Acyl-CoA_dh_1; EKR; Fer4_16; HpaB; PFOR_II; POR; POR_N; TPP_enzyme_C
Oscillibacter valericigenes	Main OP/low-BMD candidate	GCF_000283575.1	NC_016048.1	3	PFOR_II_pathway	Fermentation / energy metabolism	[90296:111499]	6	AcetylCoA_hyd_C; EKR; Fer4_16; HpaB; PFOR_II; POR; POR_N; TPP_enzyme_C
Oscillibacter valericigenes	Main OP/low-BMD candidate	GCF_000283575.1	NC_016048.1	5	PFOR_II_pathway	Fermentation / energy metabolism	[334648:356534]	7	Acyl-CoA_dh_1; EKR; Fer4_16; HpaB; PFOR_II; POR; POR_N; TPP_enzyme_C
Oscillibacter valericigenes	Main OP/low-BMD candidate	GCF_000283575.1	NC_016048.1	19	PFOR_II_pathway	Fermentation / energy metabolism	[2705872:2727513]	9	Acyl-CoA_dh_1; EKR; Fer4_16; HpaB; PFOR_II; POR; POR_N; TPP_enzyme_C
Oscillibacter valericigenes	Main OP/low-BMD candidate	GCF_000283575.1	NC_016048.1	21	PFOR_II_pathway	Fermentation / energy	[3318687:3342194]	3	EKR; Fer4_16; PFOR_II; POR;

						metabolism			POR_N; TPP_enzyme_C
Oscillibacter valericigenes	Main OP/low-BMD candidate	GCF_000283575.1	NC_016048.1	7	glutamate2butyric	Other / unassigned	[1004820:1036565]	13	BcrAD_BadFG; Biotin_lipoyl; Carboxyl_trans; CoA_trans; CoA_trans_6_Glutaconate; HG2B1; HG2B2; Hacid_dh; Hacid_dh_C; OAD_beta; OAD_gamma
Oscillibacter valericigenes	Main OP/low-BMD candidate	GCF_000283575.1	NC_016048.1	15	Others_HGD_unassigned	Aromatic / phenolic metabolism	[1907104:1930658]	6	BcrAD_BadFG; HGD-D
Oscillibacter valericigenes	Main OP/low-BMD candidate	GCF_000283575.1	NC_016048.1	8	GR_fatty_acids	Lipid / fatty-acid metabolism	[1067711:1095260]	12	PFL-like; adh_short_C2
Oscillibacter valericigenes	Main OP/low-BMD candidate	GCF_000283575.1	NC_016048.1	9	xanthine_dehydrogenase	Redox / nitrogen	[1126838:1149645]	6	Ald_Xan_dh_C; FAD_binding_5; Fer2;

						metabolism			Fer2_2; MoCoBD_1; MoCoBD_2
Oscillibacter valericigenes	Main OP/low-BMD candidate	GCF_000283575.1	NC_016048.1	13	xanthine_dehydrogenase	Redox / nitrogen metabolism	[1643517:1670883]	5	Ald_Xan_dh_C; FAD_binding_5; Fer2; Fer2_2; MoCoBD_1; MoCoBD_2
Oscillibacter valericigenes	Main OP/low-BMD candidate	GCF_000283575.1	NC_016048.1	11	Arginine2putrescine	Amino-acid metabolism	[1194795:1218702]	9	AdoMet_dc; Arginase; OKR_DC_1_7_arginine; Orn_DAP_Arg_deC; Sacchrp_dh_C; Spermine_synt_N; Spermine_synth
Oscillibacter valericigenes	Main OP/low-BMD candidate	GCF_000283575.1	NC_016048.1	10	Putrescine2spermidine	Polyamine metabolism	[1194795:1221049]	9	AdoMet_dc; Arginase; OKR_DC_1_7_arginine; Orn_DAP_Arg_deC; Sacchrp_dh_C; Spermine_synt_N; Spermine_synth
Oscillibacter valericigenes	Main OP/low-BMD candidate	GCF_000283575.1	NC_016048.1	12	acetate2butyrate	Fermentation / energy	[1544602:1578885]	12	Acyl-CoA_dh_1; EC1_2-faecali; ETF;

						metabolism			ETF_alpha; HCDH; HCDH_N
Oscillibacter valericigenes	Main OP/low-BMD candidate	GCF_000283575.1	NC_016048.1	14	GR_eut-pdu-related	Other / unassigned	[1673108:1708505]	17	Aldedh; EutN_CcmL; Gly_radical; PFL-like; PTAC; PduV-EutP
Oscillibacter valericigenes	Main OP/low-BMD candidate	GCF_000283575.1	NC_016048.1	16	gallic_acid_met	Aromatic / phenolic metabolism	[2184096:2205974]	7	Flavoprotein; gallic_acid
Oscillibacter valericigenes	Main OP/low-BMD candidate	GCF_000283575.1	NC_016048.1	17	Leucine_reduction	Other / unassigned	[2281426:2317404]	11	BcrAD_BadFG; CoA_transf_3; HCDH; Hacid_dh_C; HleuS2; Lactoyl-CoA1; Thiolase_C; Thiolase_N
Oscillibacter valericigenes	Main OP/low-BMD candidate	GCF_000283575.1	NC_016048.1	20	Flavoenzyme_lipids_catabolism	Lipid / fatty-acid metabolism	[3121535:3150833]	11	Acyl-CoA_dh_1; CoA_trans; HpaB; baiH
Oscillibacter valericigenes	Main OP/low-BMD candidate	GCF_000283575.1	NC_016048.1	22	porA	Fermentation / energy metabolism	[3577194:3599786]	7	Fer4_7; PFOR_II; POR; POR_N; TPP_enzyme_C

Oscillibacter valericigenes	Main OP/low-BMD candidate	GCF_000283575.1	NC_016048.1	23	caffeate_respiration	Other / unassigned	[3824507:3853495]	11	Acyl-CoA_dh_1; Acyl-CoA_dh_M; CoA_trans_2_CAF; HpaB; HpaB_N
Parabacteroides distasonis	Main OP/low-BMD candidate	GCF_024758965.1	NZ_CP103099.1	1	TPP_AA_metabolism	Amino-acid metabolism	[365937:391905]	6	ALS_ss_C; ILVD_EDD; IlvC; TPP_enzyme_C
Parabacteroides distasonis	Main OP/low-BMD candidate	GCF_024758965.1	NZ_CP103099.1	2	Fumarate2succinate	Fermentation / energy metabolism	[1856612:1880083]	4	FAD_binding_2; Fer2_3; SucD_FumR; Succ_DH_flav_C
Parabacteroides distasonis	Main OP/low-BMD candidate	GCF_024758965.1	NZ_CP103099.1	3	Pyruvate2acetate-formate	Fermentation / energy metabolism	[2062293:2085270]	4	PFL_acetate2; Radical_SAM
Parabacteroides distasonis	Main OP/low-BMD candidate	GCF_024758965.1	NZ_CP103099.1	4	succinate2propionate	Fermentation / energy metabolism	[2984965:3009494]	10	Biotin_lipoyl; CT_Btheta; Glyoxalase_4; OAD_beta; OAD_gamma

Parabacteroides distasonis	Main OP/low-BMD candidate	GCF_024758965.1	NZ_CP103099.1	5	porA	Fermentation / energy metabolism	[3586001:3608837]	5	Fer4_7; PFOR_II; POR; POR_N; TPP_enzyme_C
Parabacteroides distasonis	Main OP/low-BMD candidate	GCF_024758965.1	NZ_CP103099.1	6	TPP_fatty_acids	Lipid / fatty-acid metabolism	[3900537:3925395]	8	ECH_1; HBT; TPP_enzyme_C
Parabacteroides distasonis	Main OP/low-BMD candidate	GCF_024758965.1	NZ_CP103099.1	7	Sulfate2PAPS	Sulfur metabolism	[4365110:4388094]	4	APS_kinase; GTP_EFTU; PAPS_reduct
Parabacteroides distasonis	Main OP/low-BMD candidate	GCF_024758965.1	NZ_CP103099.1	8	Rnf_complex	Fermentation / energy metabolism	[5203366:5228427]	8	Complex1_51K; FMN_bind; FeS; Fer4; NQR2_RnfD_RnfE; Rnf-Nqr; RnfC_N; SLBB
Parabacteroides distasonis	Main OP/low-BMD candidate	GCF_024758965.1	NZ_CP103099.1	10	Phenylpyruvate_ferredoxin_oxidoreductase	Fermentation / energy metabolism	[5452868:5475048]	5	CoA_binding_2; POR; POR_N; Succ_CoA_lig; TPP_enzyme_C
Parabacteroides distasonis	Main OP/low-BMD candidate	GCF_024758965.1	NZ_CP103099.1	9	OD_unknown	Other / unassigned	[5452868:5479308]	8	CoA_binding_2; POR; POR_N;

									Succ_CoA_lig; TPP_enzyme_C
Roseburia intestinalis	Normal-BMD-associated comparator	GCF_900537995.1	NZ_LR027880.1	1	porA	Fermentation / energy metabolism	[54787:78257]	7	Fer4_7; PFOR_II; POR; POR_N; TPP_enzyme_C
Roseburia intestinalis	Normal-BMD-associated comparator	GCF_900537995.1	NZ_LR027880.1	2	acetate2butyrate	Fermentation / energy metabolism	[642854:668098]	8	Acyl-CoA_dh_1; ETF; ETF_alpha; HCDH; HCDH_N; Thiolase_C; Thiolase_N
Roseburia intestinalis	Normal-BMD-associated comparator	GCF_900537995.1	NZ_LR027880.1	3	xanthine_dehydrogenase	Redox / nitrogen metabolism	[2115617:2139281]	5	Ald_Xan_dh_C; FAD_binding_5; Fer2; Fer2_2; MoCoBD_1; MoCoBD_2
Roseburia intestinalis	Normal-BMD-associated comparator	GCF_900537995.1	NZ_LR027880.1	4	Rnf_complex	Fermentation / energy metabolism	[2259536:2286728]	11	Complex1_51K; FMN_bind; FeS; Fer4; Fer4_9; NQR2_RnfD_RnfE; Rnf-Nqr; RnfC_N; SLBB

Roseburia intestinalis	Normal-BMD-associated comparator	GCF_900537995.1	NZ_LR027880.1	5	succinate2propionate	Fermentation / energy metabolism	[2278781:2304034]	12	Biotin_lipoyl; CT_Vparvula; HMGL-like; OAD_beta; OAD_gamma; PYC_OADA
Roseburia intestinalis	Normal-BMD-associated comparator	GCF_900537995.1	NZ_LR027880.1	6	Putrescine2spermidine	Polyamine metabolism	[3070868:3094310]	8	Orn_DAP_Arg_deC; Sacchrp_dh_C; Spermine_synt_N; Spermine_synth
Roseburia intestinalis	Normal-BMD-associated comparator	GCF_900537995.1	NZ_LR027880.1	7	Others_HGD_unassigned	Aromatic / phenolic metabolism	[3145613:3169501]	4	BcrAD_BadFG; HGD-D
Roseburia intestinalis	Normal-BMD-associated comparator	GCF_900537995.1	NZ_LR027880.1	8	Pyruvate2acetate-formate	Fermentation / energy metabolism	[3615205:3638200]	6	PFL_acetate2; Radical_SAM
Roseburia intestinalis	Normal-BMD-associated comparator	GCF_900537995.1	NZ_LR027880.1	9	Adenylylsulfate_reductase	Sulfur metabolism	[3688597:3710624]	10	FAD_binding_2; Fer4_9
Ruminococcus torques	Normal-BMD-associated comparator	GCF_044591705.1	NZ_CP172594.1	1	Others_HGD_unassigned	Aromatic / phenolic	[750103:774360]	5	BcrAD_BadFG; HGD-D

						metabolism			
Ruminococcus torques	Normal-BMD-associated comparator	GCF_044591705.1	NZ_CP172594.1	2	Rnf_complex	Fermentation / energy metabolism	[966864:991424]	13	Complex1_51K; FMN_bind; FeS; Fer4; HMGL-like; NQR2_RnfD_RnfE; OAD_beta; PYC_OADA; Rnf-Nqr; RnfC_N; SLBB
Ruminococcus torques	Normal-BMD-associated comparator	GCF_044591705.1	NZ_CP172594.1	5	Rnf_complex	Fermentation / energy metabolism	[1424415:1451014]	10	FeS; Fer4; SLBB
Ruminococcus torques	Normal-BMD-associated comparator	GCF_044591705.1	NZ_CP172594.1	3	succinate2propionate	Fermentation / energy metabolism	[978838:1010890]	16	Biotin_lipoyl; CT_Vparvula; Complex1_51K; FMN_bind; HMGL-like; NQR2_RnfD_RnfE; OAD_beta; OAD_gamma; PYC_OADA; RnfC_N; SLBB

Ruminococcus torques	Normal-BMD-associated comparator	GCF_044591705.1	NZ_CP172594.1	4	Phenylpyruvate_ferredoxin_oxidoreductase	Fermentation / energy metabolism	[1160247:1182569]	8	POR; POR_N; TPP_enzyme_C
Ruminococcus torques	Normal-BMD-associated comparator	GCF_044591705.1	NZ_CP172594.1	6	PFOR_II_pathway	Fermentation / energy metabolism	[1789737:1813283]	2	EKR; Fer4_16; PFOR_II; POR; POR_N; TPP_enzyme_C
Ruminococcus torques	Normal-BMD-associated comparator	GCF_044591705.1	NZ_CP172594.1	7	xanthine_dehydrogenase	Redox / nitrogen metabolism	[2125061:2148686]	6	Ald_Xan_dh_C; FAD_binding_5; Fer2; Fer2_2; MoCoBD_1; MoCoBD_2
Ruminococcus torques	Normal-BMD-associated comparator	GCF_044591705.1	NZ_CP172594.1	8	TPP_AA_metabolism	Amino-acid metabolism	[2173586:2201844]	7	ALS_ss_C; Homoserine_dh; TPP_enzyme_C
Ruminococcus torques	Normal-BMD-associated comparator	GCF_044591705.1	NZ_CP172594.1	9	Pyruvate2acetate-formate	Fermentation / energy metabolism	[2246914:2270126]	8	PFL_acetate2; Radical_SAM
Ruminococcus torques	Normal-BMD-associated comparator	GCF_044591705.1	NZ_CP172594.1	10	Flavoenzyme_sugar_catabolism	Other / unassigned	[2529068:2553811]	6	Glyco_hydro_1; PTS_EIIC; baiH

Sutterella wadsworthensis	Main OP/low-BMD candidate	GCF_016724805.1	NZ_CP068055.1	1	Fumarate2succinate	Fermentation / energy metabolism	[176078:198935]	9	FAD_binding_2; Fer2_3; Succ_DH_flav_C
Sutterella wadsworthensis	Main OP/low-BMD candidate	GCF_016724805.1	NZ_CP068055.1	2	Pyruvate2acetate-formate	Fermentation / energy metabolism	[491076:514106]	8	PFL_acetate2; Radical_SAM
Sutterella wadsworthensis	Main OP/low-BMD candidate	GCF_016724805.1	NZ_CP068055.1	3	Others_HGD_unassigned	Aromatic / phenolic metabolism	[734764:759141]	9	BcrAD_BadFG; HGD-D
Sutterella wadsworthensis	Main OP/low-BMD candidate	GCF_016724805.1	NZ_CP068055.1	4	Molybdopterin_dependent_oxidoreductase	Redox / nitrogen metabolism	[1359796:1383913]	6	Fer4_11; Fer4_4; Molybdopterin; Molydop_binding; NrfD
Sutterella wadsworthensis	Main OP/low-BMD candidate	GCF_016724805.1	NZ_CP068055.1	5	Arginine2putrescine	Amino-acid metabolism	[2139566:2167827]	8	OTCace; OTCace_N; Orn_Arg_deC_N
Turicibacter sanguinis	Secondary OP candidate	GCF_013046825.1	NZ_CP053187.1	1	Others_HGD_unassigned	Aromatic / phenolic	[430006:454299]	7	BcrAD_BadFG; HGD-D

						metabolism			
Turicibacter sanguinis	Secondary OP candidate	GCF_013046825.1	NZ_CP053187.1	2	Pyruvate2acetate-formate	Fermentation / energy metabolism	[889926:912914]	5	PFL_acetate2; Radical_SAM
Turicibacter sanguinis	Secondary OP candidate	GCF_013046825.1	NZ_CP053187.1	3	Rnf_complex	Fermentation / energy metabolism	[1921696:1947518]	8	Complex1_51K; FeS; Fer4; SLBB
Turicibacter sanguinis	Secondary OP candidate	GCF_013046825.1	NZ_CP053187.1	7	Rnf_complex	Fermentation / energy metabolism	[2848810:2873507]	8	Complex1_51K; FMN_bind; FeS; NQR2_RnfD_RnfE; Rnf-Nqr; RnfC_N; SLBB
Turicibacter sanguinis	Secondary OP candidate	GCF_013046825.1	NZ_CP053187.1	4	Anaerobic_sulfite_redutase	Other / unassigned	[2237380:2260166]	10	DHODB_Fe-S_bind; Fer4_22; NAD_binding_1; NIR_SIR; NIR_SIR_ferr
Turicibacter sanguinis	Secondary OP candidate	GCF_013046825.1	NZ_CP053187.1	5	Arginine2putrescine	Amino-acid	[2598450:2622161]	4	AdoMet_dc; Arginase; OKR_DC_1_7_argini

						metabolism			ne; Spermine_synt_N; Spermine_synth
Turicibacter sanguinis	Secondary OP candidate	GCF_013046825.1	NZ_CP053187.1	6	Putrescine2spermidine	Polyamine metabolism	[2599336:2620649]	4	AdoMet_dc; Arginase; OKR_DC_1_7_arginine; Spermine_synt_N; Spermine_synth
